# A suppressor of a *wtf* poison-antidote meiotic driver acts via mimicry of the driver’s antidote

**DOI:** 10.1371/journal.pgen.1007836

**Published:** 2018-11-26

**Authors:** María Angélica Bravo Núñez, Jeffrey J. Lange, Sarah E. Zanders

**Affiliations:** 1 Stowers Institute for Medical Research, Kansas City, MO, United States of America; 2 Department of Molecular and Integrative Physiology, University of Kansas Medical Center, Kansas City, KS, United States of America; University of Rochester, UNITED STATES

## Abstract

Meiotic drivers are selfish alleles that subvert gametogenesis to increase their transmission into progeny. Drivers impose a fitness cost, putting pressure on the genome to evolve suppressors. Here we investigate the *wtf* gene family from *Schizosaccharomyces pombe*, previously shown to contain meiotic drivers in wild isolates. We discovered that *wtf13* found in lab stocks is a meiotic driver. *wtf13* kills spores that do not inherit it by generating both a diffusible poison and a spore-specific antidote. Additionally, we demonstrate that *wtf13* is suppressed by another *wtf* gene, *wtf18-2*, that arose spontaneously in the lab and makes only an antidote. Wtf18-2 does not act indiscriminately to prevent spore destruction. Instead, it rescues only the spores that inherit *wtf18-2*. In this way, *wtf18-2* selfishly gains a transmission advantage of its own while dampening the drive of *wtf13*. This establishes a novel paradigm for meiotic drive suppressors and provides insight into the mechanisms and evolution of drive systems.

## Author summary

Killer meiotic drivers are selfish genes that cause infertility by destroying the gametes (e.g. sperm) that do not inherit them. This allows them to be transmitted into up to 100% of the surviving gametes. Because of the significant fitness cost that these killer meiotic drivers impose to the organism, suppressors of drive are expected to evolve. We and others recently discovered that members of the *wtf* gene family in natural isolates of fission yeast are killer meiotic drivers. These genes generate a poison to kill gametes, but rescue those that inherit the driver with an antidote. In this work, we discovered that an additional *wtf* gene, *wtf13* found in intensely studied lab strains is also a meiotic driver. This driver is only capable of driving in the absence of another *wtf* gene, *wtf18-2*, which works by mimicking the antidote of *wtf13*. By protecting only the spores that inherit the gene, *wtf18-2* selfishly gains a transmission advantage of its own, establishing a novel paradigm for drive suppressors.

## Introduction

A founding principle of genetics is that the two alleles carried by a diploid are transmitted at Mendelian (equal) frequencies into the gametes [1]. The mechanisms of meiotic chromosome segregation and gametogenesis are often assumed to be unbiased and to therefore guarantee Mendelian transmission. These processes, however, are not perfect and are vulnerable to exploitation by ‘selfish’ genetic parasites that can bias their own transmission to the next generation, often at the expense of the rest of the genome [2]. These selfish alleles are known as meiotic drivers. Although the term was initially used to describe alleles that could bias the meiotic divisions in their favor, it soon expanded to include alleles that act after the meiotic divisions to destroy the gametes that do not inherit them [2, 3].

Meiotic drivers have been observed across eukaryotes from plants to humans [4–6]. While the true prevalence of meiotic drivers is unknown (and perhaps underappreciated), the detection of meiotic drive has accelerated in recent years, as next generation sequencing enabled the detection of drivers not linked to obvious phenotypes [7–11].

Meiotic drive typically compromises organismal fitness [12–15]. This is because Mendelian transmission facilitates natural selection of the best adapted alleles by ensuring each allele has a fair chance to be tested. Additionally, meiotic drivers can inadvertently cause deleterious alleles that are linked to drivers to be maintained or spread in a population. For example, several drive loci are genetically linked to recessive mutations that cause sterility or even lethality [13, 16–18]. One class of meiotic drivers, the killer meiotic drivers, can even cause infertility directly. These drivers act by actively destroying meiotic products that do not inherit the drive allele from a heterozygous parent [6, 19].

Genomic loci that are unlinked from drive alleles can suffer direct and indirect fitness costs imposed by drivers. This puts meiotic drivers in genetic conflict with the rest of the genome, especially alleles that are driven against and transmitted to less than half of the functional gametes. Alleles that are unlinked from the driver that suppress drive can therefore be favored by natural selection, which in turn, selects for drive alleles that evade suppression [4, 12, 20]. The evolutionary dynamic between meiotic drivers and their suppressors is akin to that observed between viruses and immune factors: both sides are predicted to rapidly evolve in an ongoing molecular arms race [21, 22]. This race between drivers and suppressors is predicted to shape the evolution of fundamental aspects of eukaryotic cells including chromosome structure and segregation [23–25].

A handful of meiotic drive alleles have been identified, and the phenotypic effects of many more have been observed. Very little, however, is known about the molecular mechanisms by which they function [4–6, 9, 13, 26–33]. Additionally, while the actions of drive suppressors have been detected in many cases, only three suppressors of drive have been identified [4, 6, 9, 11, 13, 26–37].

The *wtf* gene family of fission yeast offers an excellent opportunity to study the mechanisms and evolution of meiotic drive genes [38, 39]. In the reference strain of *S*. *pombe* (*strain L972*, denoted here as *Sp*), there are 25 *wtf* genes, including several pseudogenes [40]. Four different *wtf* genes were recently identified as meiotic drivers in two different *S*. *pombe* group isolates, *S*. *kambucha* (denoted here as *Sk*) and CBS5557 [7, 8]. These genes all act to kill spores (yeast meiotic products) that do not inherit them such that >70% of the viable spores generated by *wtf+/-* heterozygotes are *wtf+* [7, 8].

**Fig 1:**
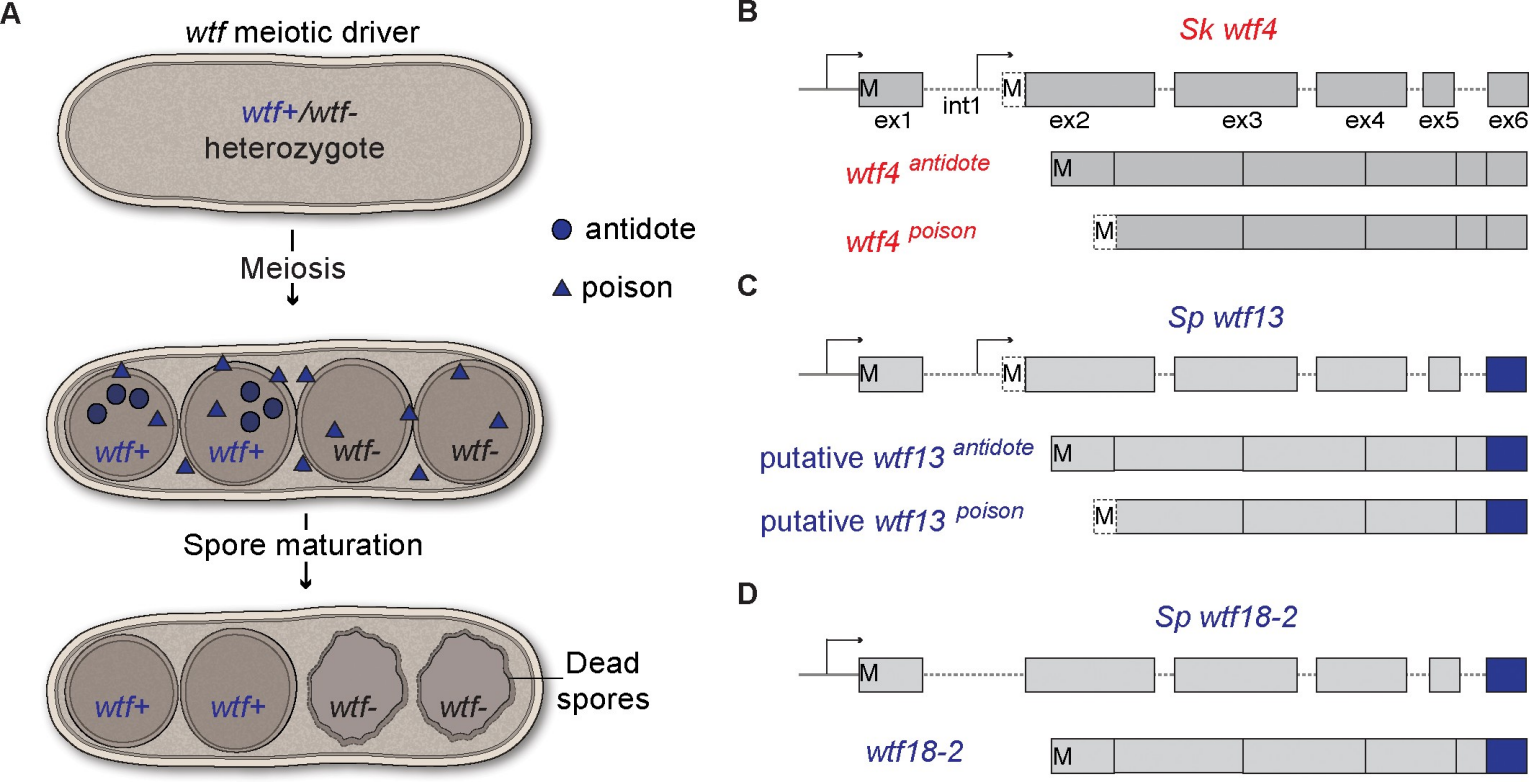
Wtf poison and antidote meiotic drive. (A) Model for how *wtf* meiotic drivers enhance their transmission into functional spores when heterozygous (*wtf*+/*wtf*-). *wtf* meiotic drivers generate both a poison and an antidote. The poison spreads throughout the ascus (sac that holds the spores). The antidote is specifically enriched within the spores that inherit the driver allele. Spores that inherit the *wtf*+ allele are thus overrepresented in the viable spore population. (B) *Sk wtf4* uses alternate transcriptional and translational start sites to generate two transcripts. The short transcript encodes a poison from five exons and the longer transcript encodes an antidote to the poison in six exons [7]. (C) Analogous to *Sk wtf4*, *Sp wtf13* makes two transcripts [41]. (D) *Sp wtf18-2* generates one transcript with six exons. To emphasize the high amino acid identity shared by exon six of *Sp wtf13* and *Sp wtf18-2*, those exons are depicted in blue. M’s represent the translational start sites.

At least one such gene, *wtf4* from *S*. *kambucha*, causes meiotic drive by a poison and antidote mechanism (Fig 1A). The *wtf4* gene has two alternative transcriptional start sites (Fig 1B). The two transcripts are in the same reading frame and are largely overlapping, but they encode distinct poison and antidote proteins. The Wtf4^poison^ protein is trans-acting and can kill all spores generated by diploids encoding the locus. However, from the four spores produced after meiosis, the two spores that inherit the *wtf4* gene generally survive because they are protected by the cis-acting Wtf4^antidote^ protein (Fig 1A) [7]. Other driving *wtf* genes are predicted to act via an analogous poison-antidote mechanism [8, 38].

Not all intact *wtf* genes, however, appear to encode two transcripts. Many *wtf* genes, including twelve in the reference genome, encode only one transcript [8, 39, 41]. In addition, all three tested intact *wtf* genes predicted to have one transcript failed to cause drive [7]. These results led us to predict that at least some of these *wtf* genes encode only antidotes and that they could act as suppressors of *wtf* drive genes [7, 38].

Here, we show that *Sp wtf13* is a poison-antidote meiotic driver. In addition, we show that *Sp wtf18-2* encodes a protein that suppresses *Sp wtf13* via molecular mimicry of the *Sp* Wtf13^antidote^ protein. Importantly, *Sp wtf18-2* only rescues gametes that inherit it from destruction by the *Sp* Wtf13^poison^ protein. In other words, *wtf18-2* benefits from the infertility caused by *wtf13*. This represents a novel type of drive suppressor in that it parasitizes the driver to gain a non-Mendelian transmission advantage of its own.

## Results

### *S*. *pombe wtf13* is an autonomous poison-antidote meiotic drive gene

Our previous work suggested that a complex network of meiotic drivers and suppressors of drive are distributed across both *Sp* and *Sk* chromosome 3’s. This inference stems from the phenotypes of a series of distinct *Sk/Sp* hybrid diploids. Each of these diploids is heterozygous for one pure *Sk* copy and one independently-derived recombinant (*Sk/Sp* mosaic) copy of chromosome 3. In some of these diploids, the mosaic chromosome was transmitted to more than 50% (73–97%) of the viable haploid spores. This phenotype suggested the existence of an *Sp* drive allele within the *Sp*-derived portion of those mosaic chromosomes (S1 Fig) [7, 18].

To identify the hypothesized *Sp* driver, we focused on a candidate region (between positions 1,397,380 and 1,810,659) shared by all the driving mosaic chromosomes (S1 Fig). We specifically searched this region for a *wtf* gene with a sequence similar to that of a known *wtf* drive gene. We found that this region contains the *Sp wtf13* gene that shares 78% amino acid identity with the *Sk wtf4* drive gene (S2A Fig). In addition, *Sp wtf13* has two types of transcripts: a long transcript that includes six exons and a shorter five exon transcript that begins within what is intron 1 of the long transcript (Fig 1C, S3A Fig) [41]. The long and short transcripts of *Sp wtf13* are reminiscent of those that encode the antidote and poison proteins, respectively, of *Sk wtf4* (Fig 1B and 1C). We therefore predicted that *Sp wtf13* is a meiotic driver and that the long transcript encodes an antidote, while the short transcript encodes a poison.

We decided to test the phenotype of *Sp wtf13* in an *Sk* genetic background, as the effects of drive are often uncovered in divergent backgrounds. *Sk* encodes a *wtf13* allele that is only 83% identical to the *Sp* allele (S2B Fig). This divergence is much higher than expected given the near sequence identity of the *Sk* and *Sp* genomes (>99% identity) [18, 42]. The *Sk wtf13* allele, however, does not appear to be a meiotic driver as it lacks an in-frame start codon near the start of exon 2 that is shared by all known drivers (S2B Fig, right).

**Fig 2:**
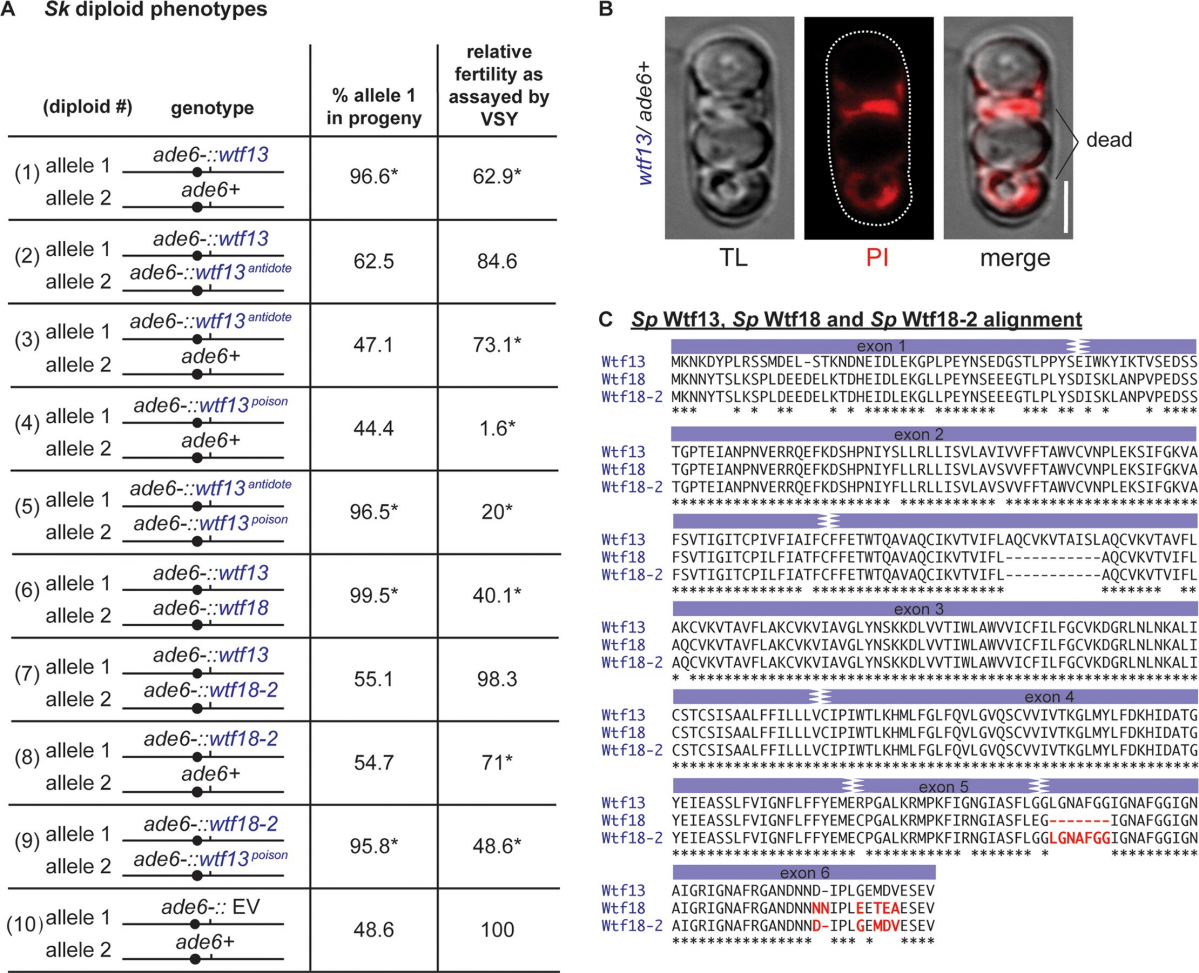
*Sp wtf13* is a meiotic driver that is suppressed by *Sp wtf18-2*. (A) Allele transmission and fertility of *Sk* diploids with the indicated constructs integrated at the *ade6* marker locus. Alleles were followed using drug resistance markers (*kanMX4* or *hphMX6*). Diploids 1–9 were compared to a wild-type empty-vector (EV) control (diploid 10) to detect biased allele transmission and differences in fertility as measured by viable spore yield. * indicates p-value of < 0.05 (G-test [allele transmission] and Wilcoxon test [fertility]). Fertility was normalized to the empty-vector control and reported as percent. More than 200 viable haploid spores were genotyped for each cross. Spores that inherited both markers and were thus geneticin^R^ Ade+, hygromycin^R^ Ade+ or hygromycin^R^ geneticin^R^ were excluded from the analyses. Raw data can be found in S1 and S2 Tables. (B) Transmitted light (TL) and fluorescent images from a tetrad produced by a *wtf13*/*ade6*+ heterozygous diploid (diploid 1) stained with propidium iodide (PI). Dead cells that lose membrane integrity stain with PI, but viable cells exclude the dye. 86% of stained tetrads exhibited this pattern (n = 58, S4 Fig). The scale bar represents 3μm. (C) Amino acid sequence alignment of *Sp* Wtf13 (long isoform), *Sp* Wtf18 and *Sp* Wtf18-2. In red are the amino acid differences between Wtf18 and Wtf18-2. Those red residues are all identical between *Sp* Wtf13 and *Sp* Wtf18-2.

To test *Sp wtf13*, we integrated the gene linked to a drug resistance marker into the *ade6* marker locus on *Sk* chromosome 3. This generated a strain that exhibits adenine auxotrophy (*ade6-*) and resistance to a drug (geneticin or hygromycin B). We then mated this haploid strain to a wild-type *Sk* haploid to generate hemizygous *Sp wtf13*/*ade6+* diploids. We then induced this diploid to sporulate (make meiotic products) and assayed its phenotype by following the *ade6* and drug resistance markers (Fig 2A, diploid 1). We found that the fertility (as measured by the viable spore yield assay [43]) of the *Sp wtf13/ade6+* diploids was significantly less than that of the empty vector/*ade6+* control (Fig 2A, diploid 1 and 10, respectively, S2 Table).

Killer meiotic drivers are expected to kill the two spores that lack the driver in each tetrad (four products of a single meiosis). To determine the pattern of spore death in tetrads generated by *Sp wtf13/ade6+* diploids, we used propidium iodide (PI) dye. PI is excluded from viable cells but can penetrate dead spores that have lost membrane integrity [7]. 86% of the tetrads contained two (live) spores that excluded PI and two (dead) spores that did not (n = 58, Fig 2B, S4A Fig). In addition, we found that the *Sp wtf13/ade6+* diploids transmitted the *Sp wtf13* allele to 97% of the viable haploid spores (Fig 2A, diploid 1). Together, these phenotypes are consistent with *Sp wtf13* acting as a meiotic driver that kills the two spores that do not inherit the gene from the *Sp wtf13*/*ade6+* hemizygous diploid. In addition, they demonstrate that *Sp wtf13* is not suppressed in *Sk*.

In additional experiments, we found that *Sp wtf13* uses a poison-antidote mechanism analogous to the previously described *Sk wtf4*. The short transcript of *Sp wtf13* encodes a poison and the long transcript encodes an antidote to the poison. By mutating the start codons in each transcript, we were also able to isolate the two functions of *Sp wtf13* in *wtf13*^*antidote*^ and *wtf13*^*poison*^ alleles (Fig 2A, diploids 2–5).

The *Sp wtf13*^*antidote*^/*ade6+ Sk* diploids did, however, have reduced fertility relative to the empty vector control (Fig 2A, compare diploids 3 and 10). In addition, the *Sp wtf13*^*antidote*^ allele only partially rescued the toxicity associated with the *Sp wtf13*^*poison*^ allele (Fig 2A, compare the fertility of diploids 1, 4 and 5). A similar antidote-only allele made by deleting intron 1 (*wtf13*^*intron1*Δ^) showed the same phenotypes as *Sp wtf13*^*antidote*^ (S1 and S2 Tables, diploids 21–23). We tested if the pattern of spore killing caused by the *wtf13*^antidote^ allele was similar to that of *Sp wtf13* via PI-staining of *Sp wtf13*^*antidote*^/*ade6+* heterozygous diploids. Most asci (72%) had 4 unstained spores. Unlike *Sp wtf13/ade6+*, the stained spores were usually found alone in a tetrad (S4C Fig). The cause of this spore viability defect is unknown. This phenotype was not detected with the analogous *Sk wtf4*^*antidote*^ allele [7].

### *S*. *pombe wtf18-2* suppresses meiotic drive of *S*. *pombe wtf13*

We previously predicted that *wtf* genes that contained only the first transcriptional start site could be suppressors of drive. Given the high amino acid identity between the known Wtf^antidote^ proteins and the Wtf^poison^ proteins they suppress, we hypothesized that the specificity between poison and antidote proteins is conferred by shared amino acid sequences [7, 38]. To identify potential suppressors of *Sp wtf13*, we looked for antidote-like *wtf* genes similar in sequence to *Sp wtf13*.

This search identified *Sp wtf18*, which shares 88% amino acid identity with the short (poison) isoform of *Sp wtf13* (Fig 2C). We next analyzed published long-read RNA sequencing data and confirmed that *Sp wtf18* generates only long (antidote-like) transcripts (Fig 1D and S3B Fig) [41].

To test if *Sp wtf18* can suppress *Sp wtf13*, we first cloned the gene using genomic DNA from our lab stocks. Interestingly, despite the generally assumed isogeny of *Sp* lab stocks (which are derived from *strain L972*), we found that the *Sp wtf18* allele found in some of our lab stocks was different from the allele found in the reference genome [44]. We call the alternate allele *Sp wtf18-2*. There are seven amino acid differences and a seven-amino acid insertion encoded in exon 6 of *wtf18-2* compared to the *wtf18* allele (Fig 2C). *Sp wtf18-2* is more similar (92% amino acid identity) to the short isoform of *Sp wtf13* than is *Sp wtf18* (88% identity). In addition, exon 6 of *Sp wtf18-2* is identical to the last exon of *Sp wtf13* (Fig 2C).

To test if *Sp wtf18* is a suppressor of the *Sp wtf13* driver, we first integrated the *Sp wtf18* allele into *Sk* at *ade6*. We then crossed the strain to the *Sk* strain described above with *Sp wtf13* at the same locus. This generated *Sp wtf13/Sp wtf18* heterozygotes (Fig 2A, diploid 6). We found that these diploids had the same phenotype as the *Sp wtf13/ade6+* hemizygotes: reduced fertility and drive of *Sp wtf13* (Fig 2A, compare diploids 1 and 6). These results indicate that the *Sp wtf18* allele is not a suppressor of *Sp wtf13*.

We next tested if the *Sp wtf18-2* allele could suppress *Sp wtf13* using the same approach described above (Fig 2A, diploid 7). We found that *Sp wtf13/Sp wtf18-2* heterozygotes had the same fertility as the empty vector control and showed Mendelian allele transmission (Fig 2A, compare diploids 1, 7 and 10). These results demonstrate that *Sp wtf18-2* acts as a suppressor of the *Sp wtf13* meiotic driver.

We next tested if, like *Spok1* in the filamentous fungus *Podospora anserina*, *Sp wtf18-2* could act not only as a suppressor, but also as a driver in heterozygotes [11]. *Sp wtf18-2* did not bias allele transmission, showing that *wtf18-2* is not an autonomous driver in the *Sk* strain background (Fig 2A, diploid 8). Interestingly, the *Sp wtf18-2*/*ade6+* diploids, similar to the *wtf13*^*antidote*^ allele, had significantly lower fertility than the empty vector control (Fig 2, diploid 8 and S4D Fig). This suggests that this allele imposes a fitness cost in the *Sk* background that we did not observe in the presence of the *Sp wtf13* driver.

We reasoned that if *wtf18-2* was working like the *wtf13*^*antidote*^, it too could rescue the toxicity caused by the *wtf13*^*poison*^. To test this, we generated diploids heterozygous for *wtf18-2* and *wtf13*^*poison*^ at the *ade6* marker locus (*Sp wtf18-2/Sp wtf13*^*poison*^). Consistent with our hypothesis, we observed transmission bias (96%) favoring the *wtf18-2* allele and an increase in fertility (Fig 2A, compare diploids 4 and 9).

### Shared sequence identity of residues encoded in exon 6 promotes the ability of suppressors to neutralize drivers

We next wanted to leverage the differential abilities of *wtf18* alleles to suppress *Sp wtf13* to provide insight into what is required for suppression. The two alleles of *wtf18* found in *Sp* are nearly identical outside of exon 6 (Fig 2C and S5 Fig). Exon 6 is identical between *Sp wtf18-2* and *Sp wtf13*. *Sp wtf18* exon 6 differs in that it has 3 copies of a seven amino acid repeat sequence, whereas the other genes have 4 copies [39]. The C-terminal region is also different in *Sp wtf18*, relative to *Sp wtf13* and *Sp wtf18-2*.

**Fig 3:**
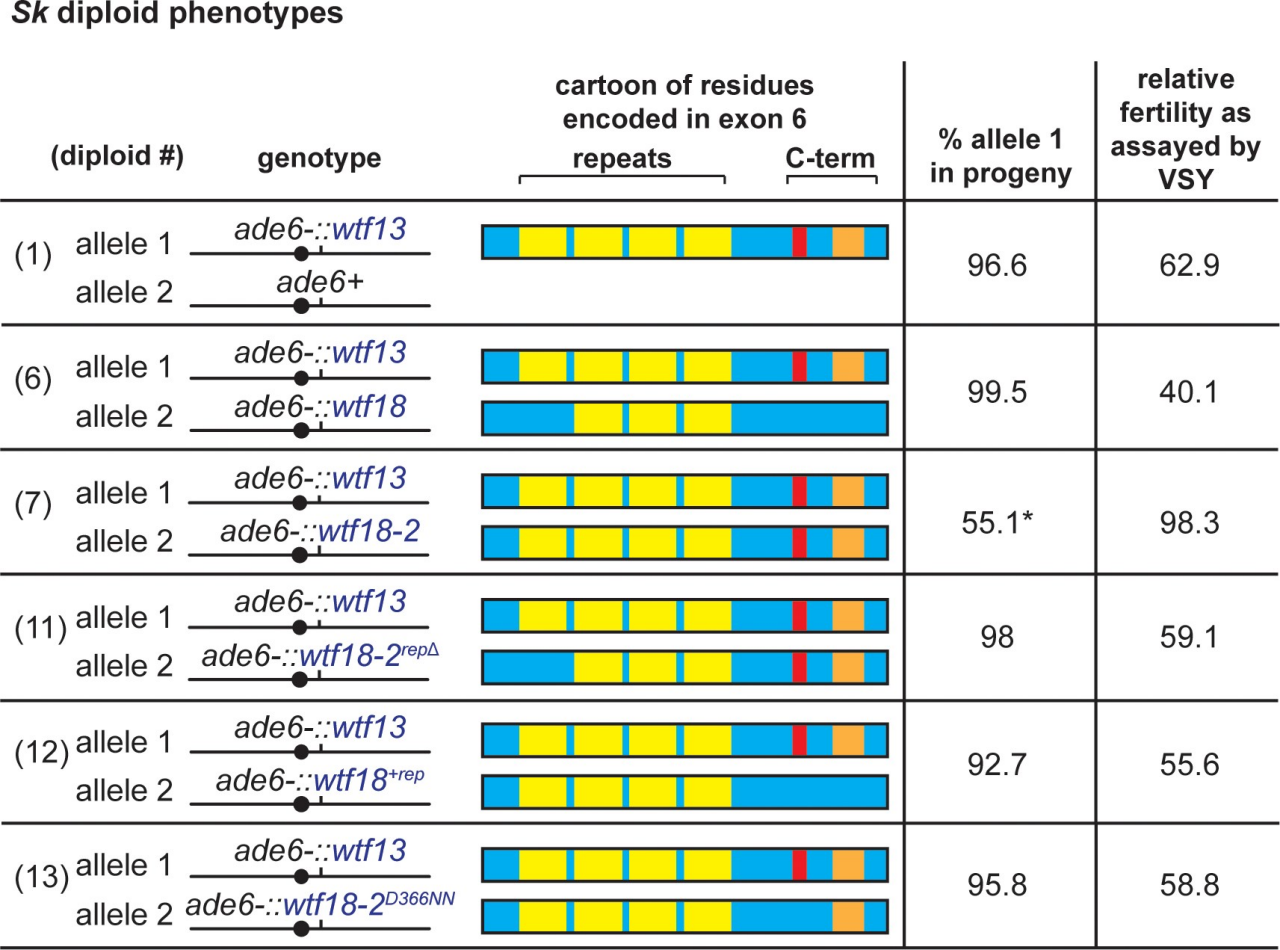
Mutations in exon 6 alter specificity between the *Sp wtf13* driver and the *Sp wtf18-2* suppressor. A cartoon of the C-terminal region, allele transmission and fertility of *Sk* diploids is shown. In the cartoon, the yellow boxes depict the number of repeat units for each allele. The red and orange boxes depict the differences between the residues at the most C-terminal region of the proteins. The diploid numbers carry over between Figures, meaning that the data for diploids 1, 6 and 7 are repeated from Fig 2. To detect if the different alleles generated still had the ability to suppress the drive phenotype, diploids 6, 7, 11–13 were compared to diploid 1. Fertility was normalized to the wild-type control (Fig 2, diploid 10). More than 200 haploid spores were genotyped for each diploid. * indicated p-value < 0.05 (G-test [allele transmission]). None of the fertility values were significantly different compared to the control (diploid 1). All raw data can be found in S1 and S2 Tables.

To probe the relevance of these changes on the ability to suppress *Sp wtf13*, we mutated *Sp wtf18-2* to make it look more like *Sp wtf18*. We found that either deleting one copy of the repeat unit or changing the entire C-terminal region both disrupted the ability of *Sp wtf18-2* to suppress *Sp wtf13* (Fig 3, diploids 11 and 12). Some more subtle changes to the C-terminal region were tolerated (S6 Fig, diploids 24 and 25), whereas the D366NN change in *Sp wtf18-*2 alone was sufficient to prevent suppression of *Sp wtf13* (Fig 3, diploid 13).

Additionally, we found that we could make a novel driver by deleting one unit of the exon 6 repeat in *Sp wtf13* (S6 Fig, diploid 26). We found that this driver could be suppressed by alleles of *wtf18* that had matching repeats (units and sequence, S6 Fig, diploid 29 and 30). Interestingly, this driver could also be suppressed by *Sp wtf18*, even though the residues in the C-terminal region did not match (S6 Fig, diploid 30).

Curiously, the *Sp wtf13*^*rep*Δ^ allele was not suppressed by the endogenous *Sk wtf18* allele present in the strain background used for this analyses (with three repeat units and matching C-terminal region; S6 Fig, diploid 26). We hypothesized the *Sk wtf18* could be silenced at its endogenous locus. To test our hypothesis, we integrated a copy of the *Sk wtf18* allele at *ade6*. Consistent with our hypothesis, *Sk wtf18* suppressed the *Sp wtf13*^*rep*Δ^ driver when both were at *ade6* (S6 Fig, diploid 31). Overall, our experiments demonstrate that sequence identity of residues encoded in exon 6 between drivers and their suppressors is functionally important. Depending on the driver, however, some mismatches between the drivers and suppressor sequences can be tolerated in the C-terminal region.

### At their endogenous loci, *Sp wtf13* acts as a driver and *Sp wtf18-2* acts as a suppressor

**Fig 4:**
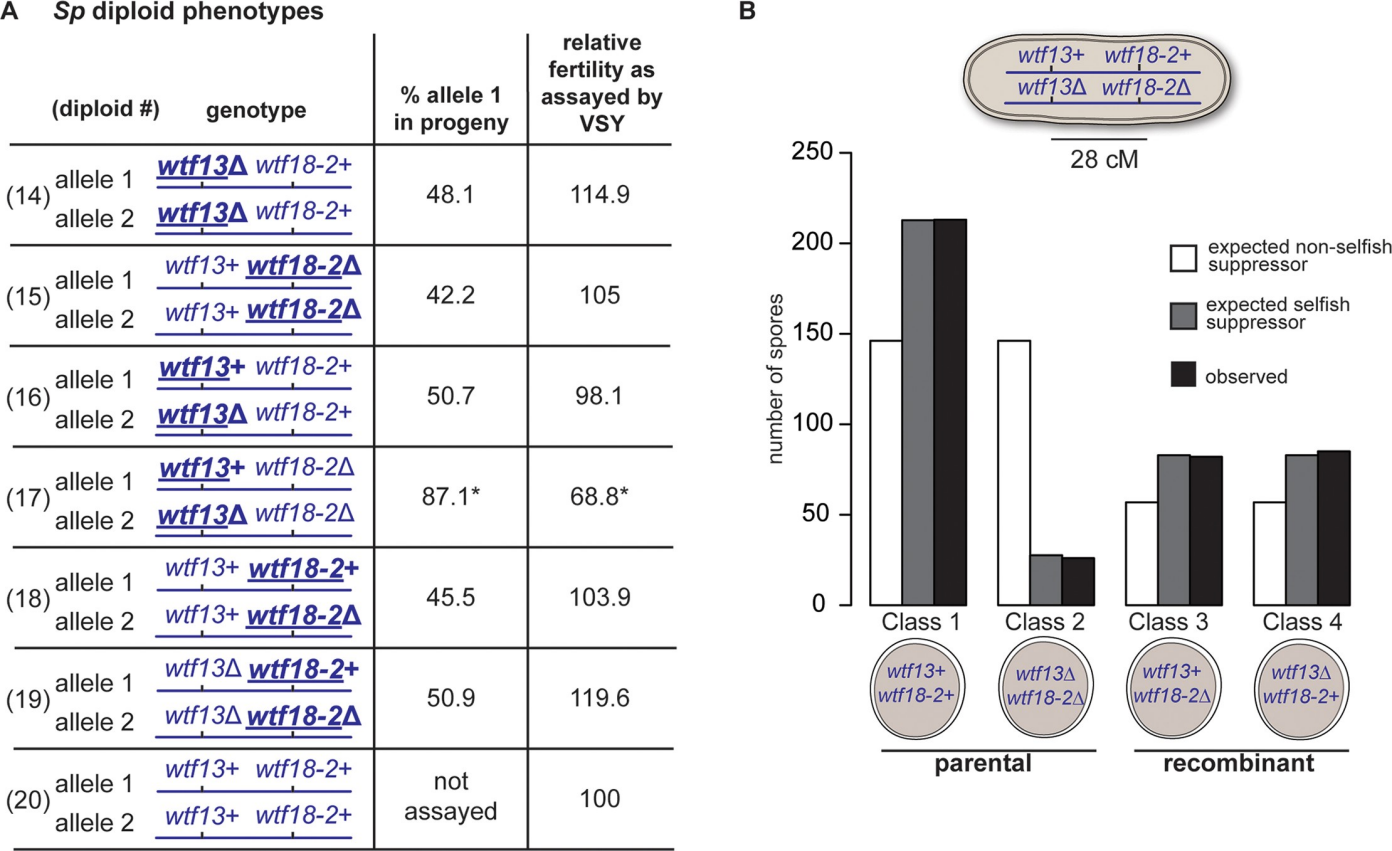
*Sp wtf13* drives only in the absence of the *Sp wtf18-2* suppressor in *Sp*. (A) Allele transmission and fertility of *Sp* diploids is shown. The allele transmission reported here is from the bold underlined locus which was assayed using drug resistance markers (*kanMX4* and/or *hphMX6*). To detect biased allele transmission, diploids 16 and 17 were compared to diploid 14 as a control. Diploids 18 and 19 were compared to diploid 15 as a control. All diploids (14–19) were compared to the wild-type control diploid (20) to detect any reduced fertility. Fertility was normalized to the wild-type control diploid (diploid 20). Allele transmission in diploid 20 was not assayed due to the lack of markers. More than 200 viable spores were genotyped for each diploid. * indicates p-value of < 0.05 (G-test [allele transmission] and Wilcoxon test [VSY]). Raw data can be found in S2 and S3 Tables. (B) The number of spores with the indicated genotypes recovered from *Sp wtf13*+/*Sp wtf13*Δ, *Sp wtf18-2*+/*Sp wtf18-2*Δ double heterozygotes is shown. *wtf13* and *wtf18-2* loci are 28cM (centiMorgans) apart, thus the parental classes are expected to be found at a higher frequency than the recombinant classes. The number of spores expected on the basis of the two models presented in the text is shown. In the first model (white boxes), the suppressor is non-selfish in that it rescues spores that do not inherit the suppressor. In the second model (grey boxes), the suppressor is selfish in that it only rescues spores that inherit the suppressor. Observed values are shown in black (n = 406). Raw data can be found in S4 Table.

We next tested the phenotypes of *Sp wtf13* and *Sp wtf18-2* in their native *Sp* genomes at their endogenous loci by characterizing deletion mutants. We found that neither gene was required for sexual reproduction (Fig 4A, diploids 14 and 15). We also confirmed our observations from the *Sk* background in that *Sp wtf13* acted as a driver and it was suppressed by *Sp wtf18-2*, but not *Sp wtf18* (Fig 4A, compare diploids 16 and 17 and S7 Fig).

In the *Sk* background, we observed a fitness cost of the *Sp wtf18-2* allele in the absence of *Sp wtf13* (Fig 2A, diploid 8). We did not observe any such cost in *Sp*, suggesting that phenotype is specific to the *Sk* strain background or the constructs used in those experiments (Fig 4A, diploids 18 and 19).

### *Sp wtf18-2* rescues only *Sp wtf13*-poisoned spores that inherit *Sp wtf18-2*

The characterized *Sk* Wtf4^antidote^ protein is enriched in the spores that inherit the locus and only rescues those spores from the *Sk* Wtf4^poison^ [7]. Similarly, the *Sp* Wtf13^antidote^ only rescues the spores that inherit the *Sp wtf13* locus (Fig 2). We hypothesized that the *Sp* Wtf18-2 protein would act in a similar way to these antidotes. In other words, we predicted only those spores that inherit *Sp wtf18-2* would be protected from killing by the *Sp wtf13* driver. This is a stark departure from the prevailing hypothesis about how suppressors of killer meiotic drivers work: indiscriminately rescuing all gametes.

To distinguish these possibilities, we assayed *Sp wtf13* and *Sp wtf18-2* double heterozygote mutants (*wtf13*+ *wtf18-2+* /*wtf13*Δ *wtf18-2*Δ). These diploids will generate four different classes of spores: 1) *wtf13*+ *wtf18-2+*, 2) *wtf13*Δ *wtf18-2*Δ, 3) *wtf13+ wtf18-2*Δ and 4) *wtf13*Δ *wtf18-2+*. The two genes are linked (28 cM, see Methods), so not all spore types should be generated in equal frequencies. If the *Sp wtf18-2* suppressor protects only the spores that inherit it, the spores that do not inherit the driver (*Sp wtf13*) or the suppressor will die (Fig 4B, Class 2). In contrast, if *wtf18-2* protects every spore, there should be no spore killing in these diploids.

We observed that the spores that lacked both the driver and the suppressor (Fig 4B, Class 2) were underrepresented (8-fold) compared to the spores that had both the meiotic driver and its suppressor (Fig 4B, Class 1). Importantly, the drive of *Sp wtf13* amongst the parental classes (Fig 4B, Classes 1 and 2) was 89%, which is indistinguishable from the 87% drive of *Sp wtf13* observed in diploids lacking *Sp wtf18-2* (Fig 4A, diploid 17). We observed analogous results using totally unlinked tagged alleles of *Sp wtf13* and *Sp wtf18-2* (S4 Table, diploid 32).

These results demonstrate that the *wtf18-2* suppressor is spore-specific, like the antidote of *Sk wtf4*. In other words, *Sp wtf18-2* acts like a selfish element by protecting only the spores that inherit *Sp wtf18-2*. Interestingly, this tactic allows *Sp wtf18-2* allele to also gain a transmission advantage (73.4% transmission) in this diploid equivalent to that enjoyed by the *Sp wtf13* drive allele (72.7%, Fig 4B). Effectively, *Sp wtf18-2* parasitizes the ability of *Sp wtf13* to drive. In the process, *Sp wtf18-2* dampens the transmission advantage enjoyed by *Sp wtf13*.

### Wtf18-2 mimics the Wtf13^antidote^

To assay the relationship between *Sp* Wtf13^poison^, *Sp* Wtf13^antidote^ and *Sp* Wtf18-2 proteins, we generated fluorescently tagged alleles. The tagged *Sp wtf13* separation-of-function (i.e. antidote-only and poison-only) alleles we made were not functional. The *Sp wtf13*^*antidote*^*-YFP* allele we generated was unable to suppress drive of *Sp wtf13* and the fertility of diploids hemizygous for the *Sp wtf13*^*poison*^*-mTurquoise2* allele we generated was ten-fold higher than we observed for diploids hemizygous for the untagged *Sp wtf13*^*poison*^ allele.

Instead, we generated an allele that tags both proteins at the shared C-terminus with YFP. We introduced this allele at *ade6* in *Sk*, as described abov*e*. The *Sp wtf13-*YFP allele does cause meiotic drive (71% transmission in *Sp wtf13-*YFP/*ade6+* heterozygotes, S1 Table, diploid 33), but the phenotype is weaker than the untagged allele (97% transmission, Fig 2A, diploid 1). We also generated a second *Sp wtf13-*YFP allele integrated at the *lys4* locus in *Sk*. For unknown reasons, this allele had the same phenotype as the untagged allele (96% transmission in *Sp wtf13-YFP/lys4+* diploids; S1 Table, diploid 34). It is not clear why the antidote in *Sp wtf13-*YFP is functional, whereas the *Sp wtf13*^*antidote*^*-YFP* was not.

**Fig 5:**
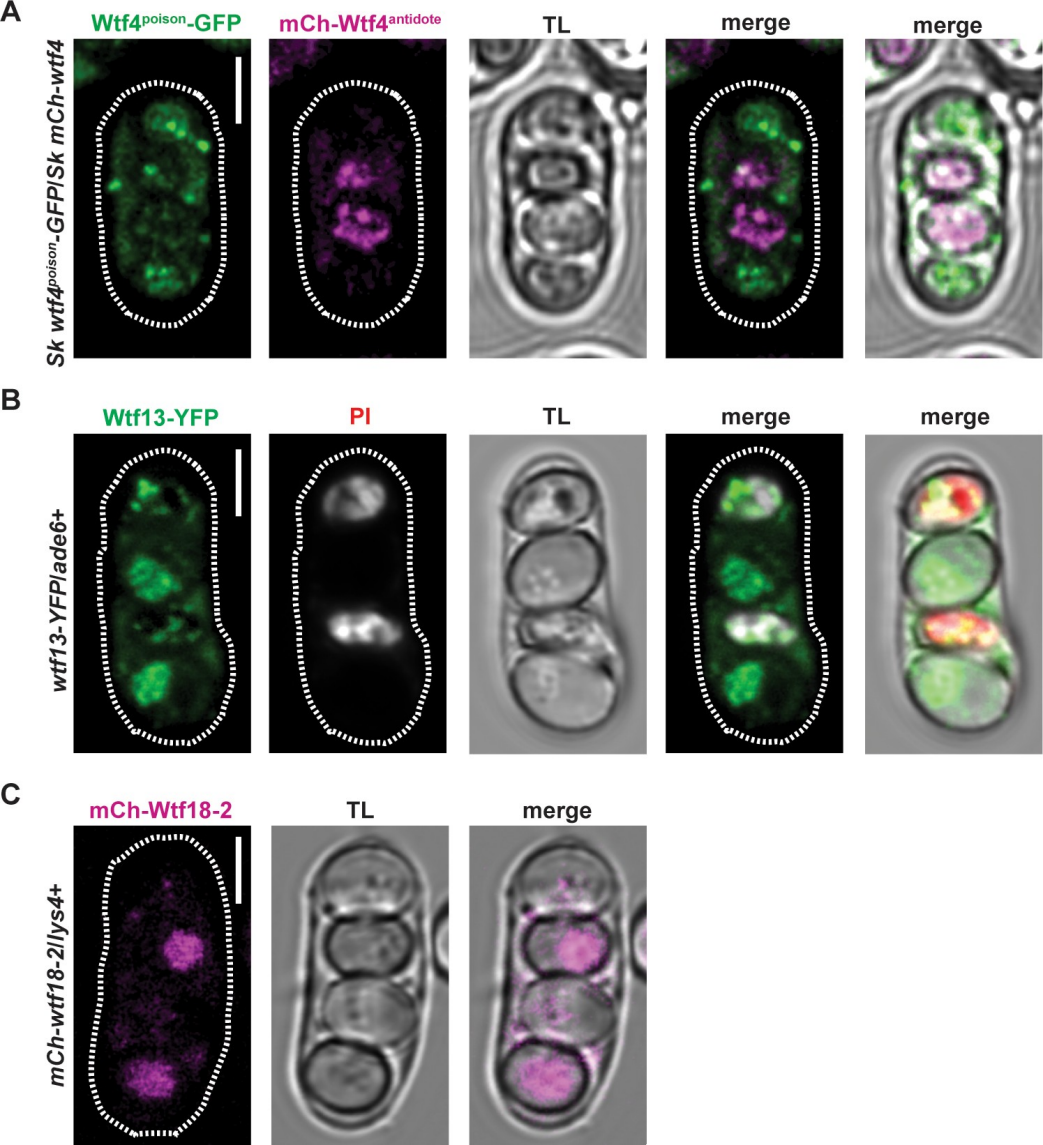
*Sp* Wtf18-2 shows an antidote-only localization pattern. Localization of *Sk* Wtf4, *Sp* Wtf13 and *Sp* Wtf18-2 fluorescently tagged proteins in tetrads. (A) Tetrad showing the localization of *Sk* Wtf4^antidote^ (magenta) and Wtf4^poison^ (green) and transmitted light (TL) using transgenes in *Sp* [7]. (B) Analogous to the Wtf4^antidote^, *Sp* Wtf13-YFP (green) is enriched in two out of the four spores. Similar to the Wtf4^poison^, Wtf13-YFP also contains diffuse cytoplasmic signal and puncta throughout the tetrad. This image is of the weaker *Sp wtf13-YFP* transgene introduced at *ade6* in *Sk*. We observe the same localization in the stronger *Sp wtf13-YFP* allele at *lys4* (S8 Fig). Wtf13-YFP is enriched in the two (living) spores that do not stain with PI. This indicates that the YFP is enriched in the spores that inherit *wtf13-YFP*, as that allele is overrepresented amongst the viable spores. This image was linearly unmixed. (C) mCh-Wtf18-2 (magenta) is enriched in half of the spores generated by *mCh-wtf18-2/lys4*+ heterozygotes. This image was linearly unmixed (see Materials and Methods, S9A Fig). Brightness and contrast were adjusted differently for each image and the images were smoothed using Gaussian blur. The scale bar represents 3μm.

In tetrads generated by diploids heterozygous for either *Sp wtf13-YFP* allele, we observed a cytoplasmic haze of YFP and YFP puncta throughout the spore-sac (Fig 5B, S8 Fig). We infer this localization represents the Wtf13^poison^, based on comparison to the *Sk* Wtf4^poison^ (Fig 5A, green). In addition to the presumed poison localization, we observed *Sp* Wtf13-YFP enriched in two of the four spores (Fig 5B, S8 Fig). This localization is reminiscent of the previously characterized *Sk* Wtf4^antidote^ protein, which is enriched in the spores that inherit the allele (Fig 5A, magenta). Like the *Sk* Wtf4^antidote^, the *Sp* Wtf13-YFP signal is enriched in the two spores that inherit the *Sp wtf13-YFP* allele and survive as the other two spores generally stain with PI (Fig 5B, S8 Fig). We therefore infer the spore-enriched *Sp* Wtf13-YFP population represents the Wtf13^antidote^.

To detect the localization of *Sp* Wtf18-2, we generated an *Sp mCherry-wtf18-2* allele and introduced the construct at the *lys4* locus in *Sk*. The *Sp mCherry*-*wtf18-2* allele is functional as it rescues the drive phenotype of the weaker *wtf13-YFP* allele integrated at *ade6* (S4 Table, diploid 32). mCherry-*wtf18-2* also partially rescues the drive phenotype of the stronger *Sp wtf13-YFP* allele at *lys4* (S1 Table, diploid 35). In tetrads generated by diploids heterozygous for *Sp mCherry-wtf18-2*, we observed *Sp* mCherry-Wtf18-2 enriched in two out of the four spores (Fig 5C, S9A Fig).

**Fig 6:**
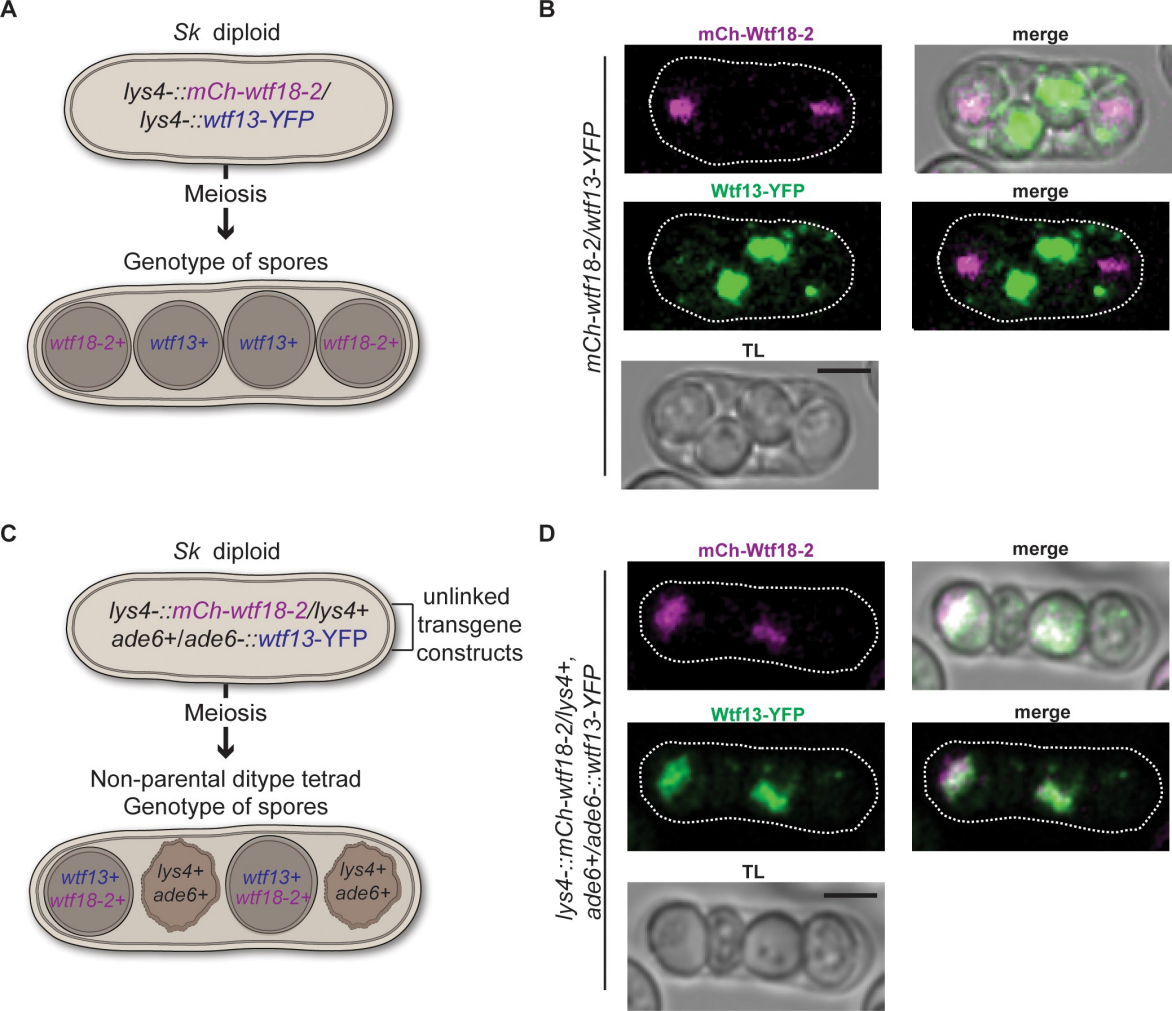
*Sp* mCh-Wtf18-2 and *Sp* Wtf13-YFP colocalize. (A) A cartoon depicting the assayed *Sp wtf13-YFP/ Sp mCh-wtf18-2* heterozygous diploids. Both transgene constructs are integrated at *lys4* in *Sk*. (B) The localization of *Sp* Wtf13-YFP (green) and *Sp* mCh-Wtf18-2 (magenta) is shown in a tetrad. (C) A cartoon of the assayed diploid heterozygous for *Sp mCh-wtf18-2* and *Sp wtf13-YFP* is shown. *mCh-wtf18-2* was integrated at *lys4* and *Sp wtf13-YFP* was integrated at *ade6* in *Sk*. *lys4* and *ade6* are unlinked, so the alleles will segregate randomly into spores yielding three tetrad types (S10 Fig). The genotype of a non-parental ditype tetrad is shown in the cartoon. (D) The localization of *Sp* mCh-Wtf18-2 (magenta) and *Sp* Wtf13-YFP (green) is shown in a non-parental ditype tetrad. The images were linearly unmixed (S9B and S9C Fig) and smoothed using Gaussian blur. The scale bar represents 3μm.

The localization pattern of *Sp* mCherry-Wtf18-2 suggested that, like the Wtf^antidote^ proteins, Wtf18-2 is enriched in the spores that inherit the locus. We tested this in diploids heterozygous for *Sp mCherry-wtf18-2* and *Sp wtf13-YFP* at *lys4* (Fig 6A). Two spores from these diploids will inherit *Sp mCherry-wtf18-2* and the other two will inherit *Sp wtf13-YFP*. We can tell which spores inherited *Sp wtf13-YFP* because the protein is enriched in those spores (Fig 5B and S8 Fig). The spores without YFP enrichment, therefore, represent those that inherited *Sp mCherry-wtf18-2*. We observed that *Sp mCherry-wtf18-2*/*Sp wtf13-YFP* heterozygous diploids generated tetrads in which two spores were enriched with YFP and the others displayed enriched mCherry signal (Fig 6B and S9B Fig). These results support the hypothesis that *Sp* Wtf18-2 is enriched in the spores that inherit the allele.

The strong similarity between the pattern of localization of *Sp* mCherry-Wtf18-2 and what we infer to be the *Sp* Wtf13^antidote^-YFP suggested those proteins may localize to the same region. To test this idea, we generated *Sk* diploids hemizygous for tagged alleles of both *Sp wtf13* and *Sp wtf18-2*. The two tagged alleles (*Sp wtf13-YFP* and *Sp mCh-wtf18-2*) were integrated at unlinked loci (*ade6* and *lys4*, respectively), so they should independently segregate into spores (Fig 6C). In a subset of tetrads, two spores will inherit both tagged alleles (Fig 6C and S10A Fig). In these tetrads, we observed co-localization between the YFP and mCherry signals (Fig 6D, S9C, S10B and S11 Figs). These results indicate that the *Sp* Wtf13^antidote^ and *Sp* Wtf18-2 proteins co-localize.

## Discussion

### *wtf13* is an autonomous poison-antidote meiotic driver

This study identifies the *S*. *pombe wtf13* gene as an autonomous killer meiotic driver (Fig 2). In general, *Sp wtf13* acts very much like the previously characterized *Sk wtf4* driver. *Sp wtf13* joins four other proven *wtf* meiotic drive genes found in wild isolates of the *S*. *pombe* group: *wtf4* and *wtf28* in *Sk*, and *cw9* and *cw27* in CBS5557 [7, 8]. The dual-transcript poison-antidote mechanism used by *Sp wtf13* appears to be homologous to the mechanisms employed by the other *wtf* drivers.

**Fig 7:**
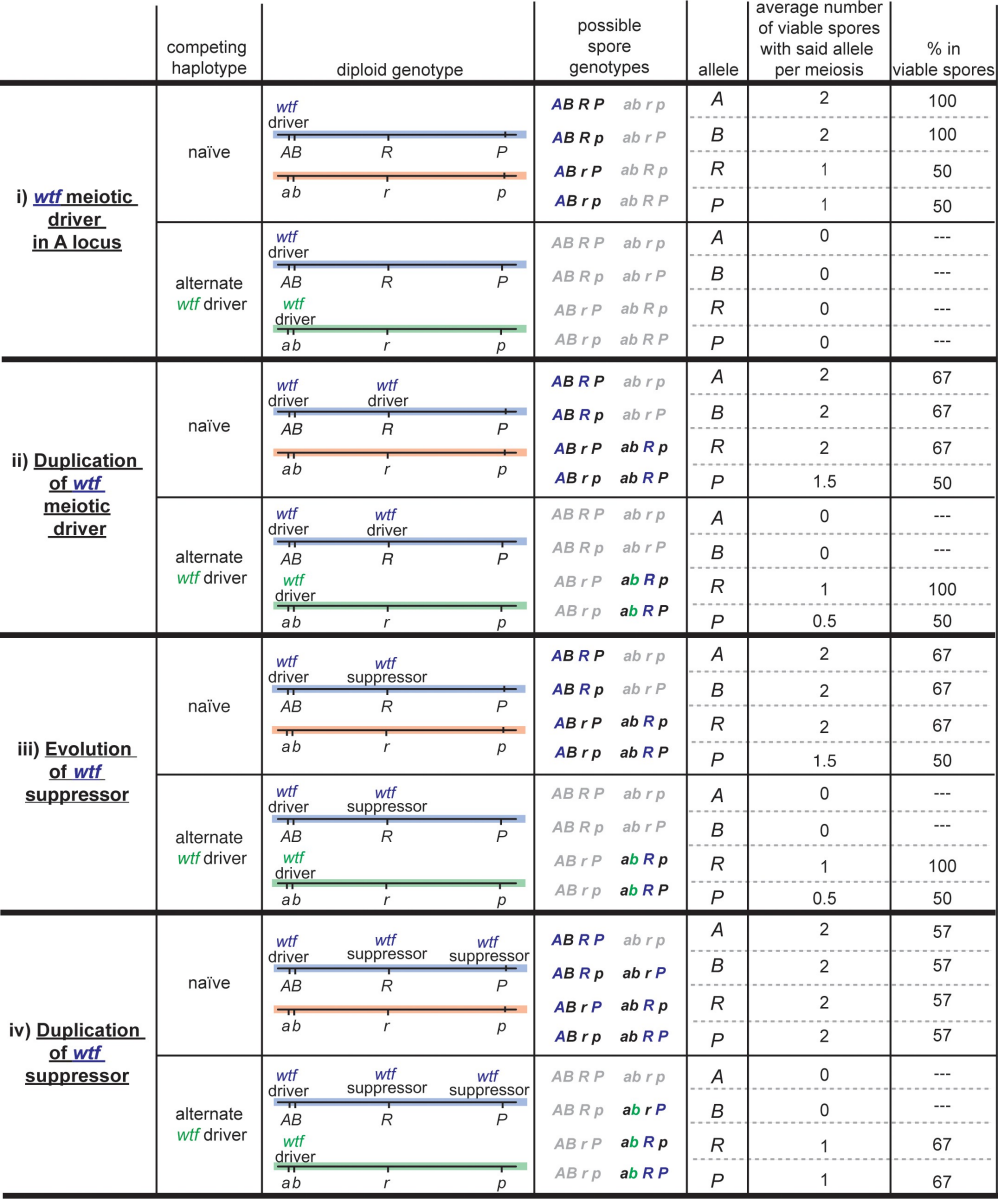
Hypothetical expansion of the *wtf* gene family. The colored lines represent chromosomes with 4 key loci (*A*, *B*, *R* and *P*). The spore genotypes are depicted in gray if the spores are inviable. At each level of the table (i-iv), the blue chromosome is the ‘experimental’ chromosome. (**i**) The blue chromosome has one meiotic driver at the *A* locus. (**ii**) The meiotic driver at the *A* locus generated a duplicate copy at the *R* locus on the blue chromosome. (**iii**) The poison function of the meiotic driver at *R* degenerated leaving an antidote-only suppressor in its place on the blue chromosome. (**iv**) The *wtf* suppressor at the *R* locus generated a duplicate gene at the *P* locus on the blue chromosome. In each scenario (**i-iv**), we consider the evolutionary success of the alleles on the blue chromosome when competed against two alternate chromosomes for transmission into the viable spores: 1) a naïve chromosome with no meiotic drivers (red) and 2) a chromosome (green) with a *wtf* driver that is distinct from the one on the blue chromosome (i.e. the antidote from the driver on the green chromosome does not neutralize the poison generated by the driver on the blue chromosome and *vice versa*). The last three columns of the table list the average number of spores bearing the indicated allele from the blue chromosome per meiosis and the fraction of the viable spores expected to inherit the indicated allele. The *A* and *B* loci are absolutely linked, but all other loci are unlinked. We assumed the *wtf* meiotic drivers kill 100% of the spores that do not inherit a copy of the gene and the suppressors are 100% efficient at rescuing spores that inherit a suppressor.

Our results are consistent with the idea that an ancestral *wtf* gene was a dual-transcript poison-antidote meiotic driver. Novel duplications of this ancestral meiotic driver to unlinked loci (e.g. the ‘*R*’ locus in Fig 7) could have been selected based on their abilities to cause drive and protect spores from the poison of the ancestral driver (e.g. at the ‘*A*’ locus in Fig 7). This advantage would only manifest, however, upon outcrossing to a strain that is sensitive to the driver. It is important to note that population genetic analyses suggest *S*. *pomb*e rarely outcrosses [45, 46]. However, the decreased recombination rates and pervasive meiotic drive observed in outcrossed *S*. *pombe* could both minimize signatures of outcrossing [18]. Our results also support the idea that other intact *wtf* genes which make two transcript classes in *Sp* (*wtf4*, *wtf19* and *wtf23*) are also likely able to cause meiotic drive, in the absence of suppressors.

### *wtf18-2* is an antidote-only suppressor of the *wtf13* driver

**Fig 8:**
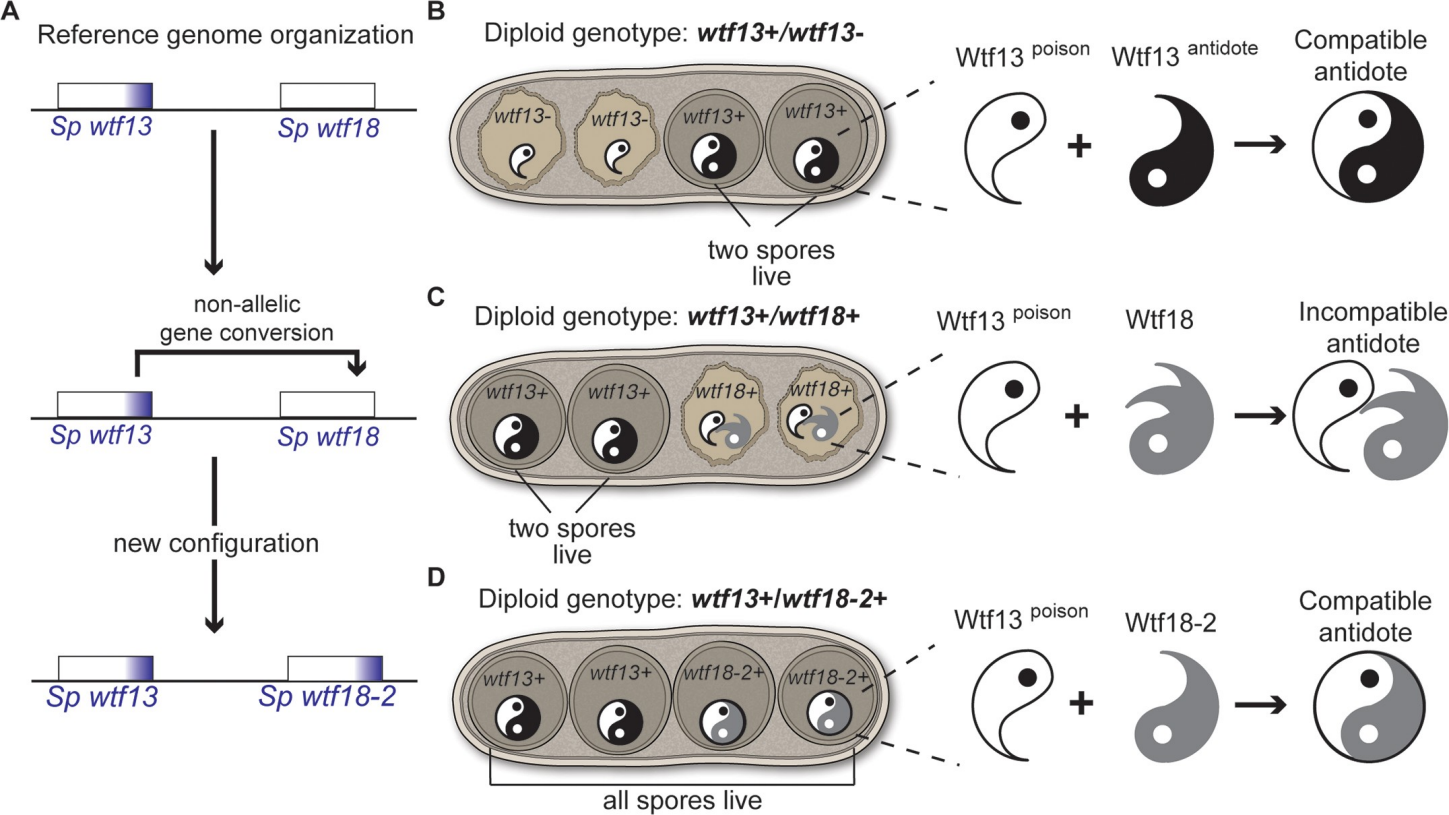
Model for *wtf* drive suppression. (A) *Sp wtf18-2* likely arose from a non-allelic gene conversion event between the *Sp wtf18* allele present in the reference genome and *Sp wtf13*. This gene conversion event provided *Sp wtf18* with the exon 6 present in *Sp wtf13*. The non-allelic conversion event happened from positions 1,580,047–1,580,343 to positions 1,806,929–1,807,206. This track includes part of *wtf13* exon 5, intron 5, exon 6 and 3’ UTR region. (B) In *wtf13* heterozygous diploids (*wtf13+/ wtf13-*), *Sp* Wtf13^poison^ has the ability to kill every spore, but the *Sp* Wtf13^antidote^ recognizes the *Sp* Wtf13^poison^ protecting the spores that inherit the *Sp wtf13+* allele. (C) In *Sp wtf13+/ Sp wtf18+* diploids, the spores that inherit the *wtf13+* allele are protected from the Wtf13^poison^ by the *Sp* Wtf13^antidote^. The two spores that inherit the *Sp wtf18+* allele die because Wtf18 does not neutralize the *Sp* Wtf13^poison^. (D) In *Sp wtf13+/ Sp wtf18-2+* diploids, the spores that inherit the *Sp wtf18-2+* allele are protected from the Wtf13^poison^ because *Sp* Wtf18-2 makes an antidote capable of neutralizing the *Sp* Wtf13^poison^. Both *Sp wtf13* and *Sp wtf18* alleles are depicted at the same locus on opposite haplotypes, like the experiments in Fig 2A.

This study also demonstrates that *Sp wtf13* can be fully suppressed by another gene, *Sp wtf18-2* (Figs 2, 3 and 4). The *Sp wtf18-2* allele arose spontaneously in the lab of Harmit Malik in a routine strain-building cross (without meiotic drive) between two *Sp* isolates. Given the 100% DNA sequence identity between *Sp wtf18-2* and *Sp wtf13* from exon 6 up to 75 bp downstream of the coding sequence, we propose the *Sp wtf18-2* allele likely arose from non-allelic gene conversion with *Sp wtf13* (Figs 2C and 8A). Indeed, non-allelic gene conversion is common within the *wtf* gene family and recombination events between these two loci have been reported [8, 39, 47].

The phenotypes of many drive suppressors have been observed, but these drive systems are generally in non-model organisms with historically limited genetic tools [13, 27, 29, 35, 48, 49]. Many of the suppressors are also found on Y chromosomes, which precludes using traditional recombination mapping to identify the causative alleles. Because of these challenges, *Sp wtf18-2* is only the fourth cloned suppressor of meiotic drive. Two of the known suppressors, the *Tmy* and *Nmy* genes are from *D*. *simulans* and both suppress the *Dox* driver. These suppressors were derived from *Dox* via retrotransposition and suppress it via a small interfering RNA (siRNA) mechanism [34, 36, 37].

The other known drive suppressor, *Spok1* of the filamentous fungus *Podospora anserina*, is also related to the gene it suppresses, *Spok2* [11]. Unlike *Nmy*, however, *Spok1* is a protein coding gene that can itself cause meiotic drive by a poison and antidote mechanism [11]. It is possible that the antidote encoded by *Spok1* can suppress both the *Spok1* and *Spok2* poisons, but the mechanism of suppression is also unknown.

Like *Nmy*, *Tmy* and *Spok1*, *wtf18*-2 shares homology with the driver it suppresses. This result supports our previously proposed model in which single transcript *wtf* genes could act as suppressors of *wtf* drivers [7, 38]. Specifically, we posited that the single transcript *wtf* genes were born initially via the degradation of an intact driver. For example, Fig 7 illustrates a hypothetical scenario in which one copy of a duplicated *wtf* driver (at the ‘*R*’ locus) loses the ability to make a poison and can only act as an antidote against the paralog (at the ‘*A*’ locus), becoming a *wtf* suppressor. In our example, this would be a neutral fitness transition for the ‘*R’* locus: it gains the same transition advantage and produces the same number of viable spores as when the locus encoded an intact duplicate of the driver at the ‘*A*’ locus in the two genetic contexts presented (Fig 7, compare ii to iii). As discussed above, this analysis pertains to outcrossing, which may be rare in *S*. *pombe* [45, 46].

It is interesting to consider if repeated driver decay is responsible for the high number (12) of intact single transcript *wtf* genes relative to intact potential drivers (4) in the reference *Sp* genome. With this question in mind, we carried our hypothetical example further. We reasoned that the suppressor at the ‘*R*’ locus could also generate an unlinked duplicate of itself to an unlinked locus (‘*P*’ in Fig 7). Interestingly, this would increase the fitness of the ‘*P*’ locus in the outcrossing scenarios considered (Fig 7, compare iii to iv). It would on average produce more viable spores per meiosis and be transmitted to a greater fraction of the viable spores (i.e. have fewer competitors). This suggests that duplication of suppressors, in addition to driver decay, could also have contributed to the expansion of the *wtf* gene family and the high number of single transcript *wtf* genes [7, 8, 40].

### Wtf18-2 suppresses drive via molecular mimicry of the Wtf13^antidote^

Our hypothesized origins of the single transcript *wtf* genes as degraded intact drivers point to a possible molecular mechanism. Specifically, if a single transcript *wtf* gene encodes an antidote protein, it could suppress a driver by mimicking the antidote of that driver (Fig 8B and 8D). Consistent with this idea, it was the high degree of shared amino acid identity with the Wtf13^poison^ that led to our discovery of the Wtf18-2 suppressor (Fig 2C).

In addition to sequence similarity, we demonstrated that the Wtf18-2 protein expression pattern is indistinguishable from that of the Wtf13^antidote^. Both proteins are highly enriched within the spores that encode them (Figs 5 and 6). Finally, we demonstrate that the Wtf18-2 and Wtf13^antidote^ proteins co-localize to the same subcellular region within spores that inherit both genes (Fig 6D and S9–S11 Figs). Overall, our results support a model in which Wtf18-2 suppresses drive by mimicking the Wtf13^antidote^ protein (Fig 8B and 8D).

How Wtf^poison^ proteins kill and how Wtf^antidotes^ prevent killing is still unknown. We and others have proposed that the poison proteins kill by oligomerizing to form a pore that could disrupt vital cellular membranes because the proteins have multiple predicted transmembrane domains (S12 Fig) [7, 8, 38]. The poison proteins can assemble into small aggregates (visible as foci in Figs 5 and 6). We speculate that these aggregates could be oligomers capable of disrupting membranes within spores. We have also proposed that the antidotes use their shared amino acid sequences to interact with the poison proteins and somehow disrupt pore formation. Our results are consistent with the idea that homotypic interactions are important, although some sequence differences between the driver and suppressor are tolerated (Fig 3 and S6 Fig).

This emerging theme of shared identity between drivers and their suppressors could guide the discovery of novel suppressors as new drivers are cloned. Additionally, this work and the work of others have shown that one potential way to engineer a suppressor of drive is to use the sequence of the driver itself. This idea will be particularly important to consider when trying to understand, predict, or alter the evolution of synthetic gene drive systems.

### *wtf18-2* selfishly suppresses the selfishness of *wtf13*

Unlinked suppressors of selfish meiotic drivers are often assumed to be beneficial alleles that are selected because they increase the fitness of their hosts [12]. The *Nmy* suppressor of *Dox* fits this paradigm. *Nmy* ensures Mendelian sex chromosome representation in the gametes, even in the 50% of gametes that do not inherit *Nmy* from a *Nmy/nmy* heterozygote [34, 36, 37]. In other words, the benefits of *Nmy* are shared by the gametes that do not inherit the suppressor. The *wtf18-2* suppressor, on the other hand, represents a more ‘selfish’ strategy that is likely a relic of the gene’s evolutionary history. Wtf18-2 only suppresses drive in the spores that inherit the *wtf18-2* allele. This tactic allows *wtf18-2* to exploit the Wtf13^poison^ protein to gain a transmission advantage of its own (Fig 4B). While this selfish suppression strategy would increase host fitness (when the driver and suppressor are not absolutely linked in *cis*), the benefits of suppression are not shared by the gametes that do not inherit the suppressor.

### Meiotic drivers drive infertility

*wtf* genes are known to be rapidly evolving in natural isolates [7, 8, 39]. Such rapid evolution is thought to be a hallmark of genes in conflict, such as meiotic drivers and their suppressors [4, 21, 22]. This work supports the idea that the rapid evolution of non-driving *wtf* genes is due to their role as suppressors of meiotic drive. The rapid evolution of *wtf* genes leads to heterozygosity of these genes in crosses between natural isolates. This *wtf* heterozygosity is known to be a major driver of infertility between different isolates of *S*. *pombe* [7, 8, 18]. Our work suggests the rapid evolution of this gene family is also generating variation *within* lab stocks that are generally considered isogenic. This heterozygosity could uncover cryptic drivers and introduce major artifacts into studies of *S*. *pombe* reproduction and fertility. More broadly, infertility caused by meiotic drivers may be common in eukaryotes [50–52].

Finally, this work demonstrates how even strong meiotic drivers can remain hidden despite intensive genetic investigation. This example underscores the challenges inherent to detecting similarly cryptic meiotic drivers in more complex systems, including humans. Furthermore, it highlights the power of investigating the evolutionary dynamics of drivers in tractable model systems.

## Materials and methods

### Plasmid construction: Integrating vectors

The genotype of the plasmids used can be found in S5 Table. To integrate transgenes linked to *kanMX4* into the *Sk* genome at *ade6+*, we used the *ade6*-targeting plasmid (pSZB188) described in [7]. For this study, we also made a cloning vector (pSZB386) that would allow us to similarly introduce transgenes linked to *hphMX6* at *ade6*. To make pSZB386, we first made a mutant *ade6* allele (with internal, 5’ and 3’ deletions in addition to introducing a central KpnI restriction site). To make the mutant *ade6* allele, we amplified the 5’ end of the *ade6* gene using oligos 588 and 589 (S6 Table). We then stitched this fragment to a second fragment of the gene that was amplified using oligos 590 and 591 using overlap PCR. We then digested the amplified fragment with BamHI and XhoI and cloned it into the BamHI and SalI sites of pAG32 [53].

To similarly integrate constructs linked to drug resistant markers at *lys4*, we generated the *lys4-*targeting vectors pSZB329 (*kanMX4*) and pSZB322 (*hphMX6*). To generate these vectors, we first purchased a mutant allele of *lys4* as a gBlock from IDT (Coralville, IA). This *lys4* allele lacks 240 bp from the beginning and end of the gene. The allele also has 50 bp deleted from the center of the gene that is replaced with a KpnI site. We blunt-cloned the gBlock into the PvuII sites of pFA6 and pAG32 to generate pSZB329 and pSZB322, respectively [53].

### Plasmid construction: Integrating vectors with *Sp wtf13* alleles

To make a construct that would allow us to introduce *Sp wtf13* into *Sk* linked to *hphMX6* at *ade6*, we first amplified the gene from *Sp* genomic DNA using oligos 879 and 880. We then digested the PCR product with SacI, and ligated it into the SacI site of pSZB188 and pSZB386 to generate pSZB368 and pSZB495, respectively.

We made the *Sp wtf13-*YFP allele using overlapping PCR. We first amplified the *Sp wtf13* gene from *Sp* genomic DNA using the forward oligo 1115 and a reverse oligo 1125 which anneals right before the stop codon. We then amplified YFP from pFA6-EYFP-HIS3MX6 [54] using oligos 1123 and 634. We then stitched both fragments using overlapping PCR. We next digested the resulting PCR product with SacI and ligated it into the SacI sites of pSZB188 and pSZB329 to generate pSZB522 and pSZB706, respectively.

To generate an antidote-only allele of *Sp wtf13*, we mutated the start site (ATG→TAC) utilized by the shorter transcript using PCR. First, using pSZB496 (same as pSZB495) as a template, we amplified both a 5’ fragment (using oligos 1115 and 1395) and a 3’ fragment (using oligos 1394 and 879) of the *Sp wtf13* gene. We then stitched the two fragments together using overlap PCR. We then digested the PCR product with SacI and ligated it into the SacI site of pSZB188 to generate pSZB702 and pSZB703.

We took a rather indirect path to generate the poison-only allele of *Sp wtf13*. We first made a tagged *wtf13*-mTurq2 allele. We made this allele by amplifying *Sp wtf13* from *Sp* genomic DNA using the forward primer 1115 and the reverse primer 1126. We also amplified mTurquoise2 from pFA6-mTurq2-URA3MX [55] using primers 1124 and 634. Using overlapping PCR, we next stitched the *wtf13* and mTurq2 PCR fragments together. We digested the resulting PCR product with SacI and ligated it into the SacI site of pSZB386 to generate pSZB526. We then used a gBlock from IDT containing 250 bp of *Sp wtf13* in which both methionine codons in exon 1 were mutated (ATG→TAG). Using the QuickChange II XL Site-Directed Mutagenesis Kit (Agilent Technologies) and the gBlock, we introduced the two mutations in exon 1 of pSZB526 to generate pSZB565. Although this allele was non-functional, we used the mutations in pSZB565 to make the *Sp wtf13* poison-only separation-of-function allele. We amplified the 5’ end of the gene (including the start site mutations) using oligos 1115 and 985 and pSZB565 as a template. We also amplified the 3’ of the gene, using pSZB495 as a template, using oligos 601 and 879. Using overlapping PCR, we stitched these two fragments to generate an *Sp wtf13* allele with both methionine codons mutated in exon 1. We then digested the PCR product with SacI and ligated it into the SacI site of pSZB386, to generate pSZB686.

To make the *wtf13*^*rep*Δ^ allele, we ordered a gBlock from IDT that contained from exon 5 to 1,078 bp downstream of the stop codon of *Sp wtf13*. In this gBlock, we deleted the 21 bp that encode the first repeat unit (LGNAFGGΔ). We amplified the 5’ region of the *Sp wtf13* gene from template pSZB368 using oligos 880 and 1530, which amplified the gene up to exon 5. We then used overlapping PCR to stitch this fragment with the gBlock, which generated an *Sp wtf13* allele with the first repeat unit deleted. We digested this allele with SacI and ligated it into the SacI site of pSZB386 to make pSZB806.

### Plasmid construction: Integrating vectors with *Sp wtf18* alleles

To make the *Sp wtf18 kanMX4 ade6*-targeting vector, we amplified the *wtf18* gene from genomic DNA (strain SZY44) using oligos 1037 and 1039. We then digested this fragment with SacI and cloned it into the SacI site of pSZB188 to generate pSZB483. We also cloned this fragment into the SacI site of pSZB386 to generate pSZB485. We amplified *Sp wtf18-2* from genomic DNA of strain SZY643 using oligos 1039 and 1202. We then digested the PCR product with SacI and ligated it into the SacI site of pSZB188 to make SZB566. We amplified the *Sk wtf18* allele from *Sk* genomic DNA (strain SZY13) using oligos 1037 and 1039. We digested this PCR product using SacI and then ligated it at the SacI site of pSZB188 to generate pSZB470.

We used overlap PCR to generate the *Sp wtf18-2* allele with an mCherry tag on the N-terminus. First, we ordered a gBlock from IDT containing the *Sp wtf18-2* promoter linked to mCherry, five glycine codons, and the first 149 bp of *Sp wtf18-2* exon 1 [56]. We amplified this fragment using oligos 1039 and 1254. We then used pSZB566 as a template to amplify *Sp wtf18-2* using oligos 1255 and 1202. Using overlap PCR, we stitched these fragments together. We then cut the resulting PCR product with SacI and ligated it into the SacI site of pSZB322 to generate pSZB625.

We used the QuickChange II XL Site-Directed Mutagenesis Kit from Agilent Technologies with pSZB566 as a template and different set of oligos to generate the different *Sp wtf18-2* mutants. We made an *Sp wtf18-2* allele that had the first repeat unit deleted (*Sp wtf18-2* ^*rep*Δ^) using oligos 1659 + 1660 to make pSZB673. To make the *wtf18-2*^*D366NN*^ allele, we used oligo 1661 with oligo 1662 to generate pSZB674. To make the *wtf18-2*^*D366N*^ allele (referred to as *Sp wtf18-2*^*mut1*^ in S6 Fig), we used oligos 1663 and 1664 and made pSZB675. Finally, to make the *wtf18-2*^*G370E*, *M372T*, *D373E*, *V374A*^ allele (referred to as *Sp wtf18-2*^*mut2*^ in S6 Fig), we used oligo 1665 + 1666 to make pSZB688.

We generated the *wtf18*^*+repeat1*^ allele using overlapping PCR. We ordered from IDT a 599 bp gBlock containing from exon 5 to the end of the 3’ UTR of *wtf18* where we introduced the 21 bp that encode for the first repeat unit (LGNAFGG) after residue 332. We amplified *wtf18* from pSZB485 using oligos 1039 and 598 and stitched the product to the gBlock we ordered using overlapping PCR. We then digested this PCR product with SacI and cloned it into the SacI site of pSZB188 to generate pSZB807 and pSZB808.

### Strain construction: Introducing alleles into yeast

All strain names and genotypes are described in S7 Table. We digested the integrating plasmids described above with KpnI (which cuts within the mutant *ade6* or *lys4* cassettes) and transformed the linear plasmids into yeast using a standard lithium acetate protocol. We selected drug resistant (geneticin or hygromycin) transformants. For alleles generated with the *ade6* targeting vectors, we picked red (*ade6*-) colonies to ensure proper integration of the transgenes at *ade6*. For alleles generated with *lys4* targeting vectors, we screened for *lys*- colonies to ensure proper integration at *lys4*.

### Deleting *wtf13* and *wtf18-2* loci in *S*. *pombe*

To delete *wtf13* at its endogenous locus, we generated DNA deletion cassettes via PCR. First, we amplified both the sequence (~1,200 bp) upstream of *Sp wtf13* using oligos 1048 and 1049, and the sequence downstream (~1,100 bp) of *Sp wtf13* using oligos 1052 and 1053. We also used oligos 1050 and 1051 to separately amplify the drug resistance genes *kanMX4* and *hphMX6* (from pFA6a and pAG32, respectively [53]). We then used overlap PCR to stitch the three PCR fragments together to make cassettes in which a drug resistance gene was flanked by the sequences up and downstream of *Sp wtf13*. We then used standard lithium acetate transformation to introduce the deletion cassettes into the genome in strain SZY643, selecting for drug resistant colonies (*wtf13*Δ::*kanMX6* or *wtf13*Δ::*hphMX6*). This generated strains SZY1444, SZY1445, SZY1440 and SZY1441. We verified the proper deletion of the gene by amplifying the locus using PCR with oligos external to the targeting cassette (oligos 1058 and 1061).

We used a similar approach to delete *Sp wtf18-2*. We first generated *wtf18-2*Δ::*kanMX6* and *wtf18-2*Δ::*hphMX6* deletion cassette using overlap PCR. We used oligos 1078 and 1079 to amplify the upstream region (~1,000 bp) of *wtf18-2* and oligos 1082 and 1083 to amplify the downstream region (~1,000 bp) of the *wtf18-2* locus. We also amplified the drug resistance genes *kanMX4* and *hphMX6* (from pFA6a and pAG32, respectively [53]) with oligos 1080 and 1081. We then stitched all the fragments together using overlap PCR to make the DNA deletion cassettes with the regions upstream and downstream of *Sp wtf18-2* flanking a drug resistance gene. We then used standard lithium acetate transformation to introduce the deletion fragments into yeast (strains SZY1442 and SZY1446). We selected for drug resistant colonies and verified strains SZY1481 and SZY1483 had the proper deletion by amplifying the locus with oligos (1113 and 1114) external to the targeting cassettes.

### Crosses for assaying fertility and meiotic drive

We generated stable diploids similar to the description in [18]. We first mixed 300 μl of a saturated culture of each parental haploid strain in a microcentrifuge tube. Then, we spun down the cells and spread them onto SPA (1% glucose, 7.3mM KH_2_PO_4_, vitamins and agar) and SPAS (SPA + 45 mg/l adenine, histidine, leucine, uracil and lysine) for ~12 hours at 25°C. We then scraped the cells off the SPA (or SPAS) plates and struck them out onto selective media (for most of the crosses: minimal yeast nitrogen-based plates). The parental strains contained complementary auxotrophies that allowed us to select for heterozygous diploids. We grew diploid cells in YEL (0.5% yeast extract, 3% glucose, 250 mg/l adenine, histidine, leucine, uracil and lysine) at 32°C with shaking overnight. We spread 50 μl of the saturated culture onto SPA to induce sporulation. We also diluted the culture and spread it onto YEA (same as YEL + agar) plates to determine the colony forming units (CFU) and to assess if the culture contained heterozygous diploids which we determined via replica plating colonies after 2–3 days of growth at 32°C. Cultures determined to not be comprised of heterozygous diploids were not assayed further. After three days on SPA, we scraped off all the cells. We then incubated the cells with glusulase (snail gut enzyme) to digest the ascus wall and release the spores. We also treated the cells with 60% ethanol to kill the remaining vegetative cells. We plated diluted spores on YEA and incubated the plates at 32°C for five days. We scored the CFU of the cells plated on YEA and assayed fertility using the viable spore yield assay (VSY = [number of spores/ number of diploids] [43]). To assay allele transmission, we determined the genotype of the progeny at control and test loci via replica plating. We generated at least two independent diploids (but usually more) and genotyped more than 200 spores per cross. We used a G-test to detect biased allele transmission compared to our control sample (S1, S3 and S4 Tables). To determine if there were any changes in the fertility of the different crosses, we assayed at least three diploids using VSY. We then used the Wilcoxon test and compared them to control diploids. All the raw values we utilized for the Wilcoxon test can be found in S2 Table.

### Linkage between *wtf13* and *wtf18* loci

To determine the recombination frequency between *wtf13* and *wtf18-2*, we used the progeny made by the *Sp wtf13 wtf18-2* double heterozygote diploid (*wtf13*+ *wtf18-2+* /*wtf13*Δ *wtf18-2*Δ; S4 Table) made by mating strains SZY643 and SZY1481 [or SZY1482]. Drive of the *Sp wtf13* allele occurs in this diploid, which prevented us from using all the progeny to calculate recombination frequencies. Instead, we only used *wtf13+* progeny which are not killed by the driver. We then divided the number of recombinants (*wtf13+ wtf18-2*Δ) by the total number of *wtf13+* progeny. We had 82 recombinants and 213 parental *wtf13+* progeny (S4 Table). This gave us the 28% recombination frequency.

### *Sp wtf18* alleles

The *Sp wtf18* allele from the reference genome is present in the lab stock strain SZY44. The *Sp wtf18-2* allele was discovered in our lab stock strain SZY643. This strain is a segregant from a cross between SZY631 and SZY629, which are themselves segregants from a cross between GP52 and GP7596. The GP strains were obtained from the collection of G.R. Smith. We sequenced the *wtf18* locus from SZY631, SZY629, GP52, and GP7596. We determined that the *wtf18-2* mutant appeared in SZY629.

### Alignments

To determine the sequence identity between our proteins, we aligned the amino acid sequences using Geneious 10.1.3 (https://www.geneious.com). Using the Geneious alignment tool, we performed global alignments with free end gap with the default parameters. We deposited the sequence of the *Sp wtf18-2*, *Sk wtf18* and *Sk wtf13* alleles to Genbank under accession numbers MH029505, MH029506 and MH029507, respectively.

### Transmembrane domain prediction

We used the topology prediction program TMHMM2.0 [57] to predict the topology of *Sp* Wtf13, Wtf18 and Wtf18-2 proteins and the number of transmembrane helices in each protein.

### Cytology

To detect if spores were being killed, we stained the asci sporulated on SPA plates with propidium iodide (PI, 1mg/ml) [7]. We scraped the cells off the plate and mixed them with 50 μl of diluted PI (1:50 in ddH20). We then incubated the cells at room temperature for 20 minutes. We then plated the cells onto a 35mm glass culture dish (MatTek) pre-coated with lectin to immobilize the cells [58]. We then imaged the cells on an LSM-780 (Zeiss) AxioObserver microscope with a 40X C-Apochromat water-immersion (Zeiss, NA = 1.2). We imaged the PI-stained cells in photon-counting channel mode with 561 nm excitation and collected the PI fluorescence through a 562–642 nm filter.

We also acquired the *Sk wtf4* images on the same LSM-780 microscope, with a 100x alpha Plan-Apochromat (Zeiss, NA = 1.46) objective. We acquired the images in photon-counting channel mode. We excited GFP at 488 nm and collected the fluorescence through a 481–552 nm bandpass filter. We excited mCherry at 561 nm and collected its fluorescence through a 572–735 nm bandpass filter.

For imaging asci, we induced meiosis of the diploid cells in liquid culture using the protocol described in Zanders et al [18]. Briefly, we made diploid cultures in YEL and incubated them at 32°C overnight. We then diluted the saturated culture 1:50 into 5 ml of PM media (8mM NA_2_HPO_4_, 2% glucose, 0.5% NH_4_Cl, EMM2 salts, vitamins and minerals) shaking at 32°C for 16 hours. To induce meiosis, we spun down 500 μl of the PM culture in a microcentrifuge tube and resuspended the cells in 5 ml of PM-N media (8mM NA_2_HPO_4_, 1% glucose, EMM2 salts, vitamins and minerals). We then incubated the samples shaking at 28°C and imaged the cells ~24 hours after the induction of sporulation. For analyses using YFP and PI, we acquired images on the same LSM-780 microscope, also with the same 40x C-Apochromat water-immersion objective. We acquired images sequentially, first in photon counting lambda mode with YFP excitation at 488 nm and with the fluorescence collected over the entire visible range. We then acquired PI images in photon counting channel mode with excitation at 561 nm and with the fluorescence collected over a bandpass of 597–735 nm. To obtain the true YFP signal from confounding autofluorescence, we linearly unmixed the YFP lambda mode data using YFP and yellow autofluorescence spectra. We recombined the unmixed YFP in ImageJ with the channel mode PI image.

To determine if *Sp* Wtf13-YFP co-localized with *Sp* mCh-Wtf18-2 (Fig 6C), we induced meiosis in diploids carrying those tagged constructs using the liquid sporulation protocol described above. Here, we also acquired the images on the LSM-780 microscope, with the objective described above, using photon-counting lambda mode with YFP and mCherry excited off-peak at 488 and 633 nm, respectively. This was done to eliminate, as much as possible, overlapping autofluorescence spectra. We collected the emission from the entire visible spectral region (~400–700 nm). After acquisition, we linearly unmixed the samples using an in-house written plug-in in ImageJ (https://imagej.nih.gov/ij/). To obtain the true fluorescence signal from the samples, we imaged control strains to obtain the YFP and mCherry spectra.

To detect if the mCherry from the *Sp* mCh-Wtf18-2 was enriched in two out of the four spores of the spore sac in a heterozygote (Fig 5C), we manually drew region of interest (ROIs) around the spores based only on transmitted light. Then, we plotted a histogram of the raw average intensities, which was weakly bimodal. We found a threshold between the peaks at an intensity of 3. We counted as positive every spore with an average intensity above 3, and negative every spore with an intensity less than 3. Every positive spore was given a score of 1 and an average value for that ascus was calculated. Enrichment in two out of the four spores will yield an average of 0.5 (2 positive out of 4 spores in the ascus). The analysis of the data can be found at the Stowers Original Data Repository (http://www.stowers.org/research/publications/libpb-1270).

To determine if mCherry and YFP co-localized inside the spores (Fig 6D, S9–S11 Figs) in *Sk* diploids hemizygous for both *Sp wtf13-YFP* and *Sp mCh-wtf18-2*, we manually drew ROIs around each of the spores in the ascus, again based only on transmitted light. We determined positive and negative mCherry and YFP spores as described above. In this case, positive YFP spores had an average intensity greater than 10 and mCherry spores had an average intensity greater than 5. We then determined how many spores were mCherry and YFP positive. The number of YFP and mCherry positive spores was then corrected to account for the double counting of the double positive spores by subtracting the number of double positive spores from both the YFP and mCherry positive spores. For example, in an ascus that has 1 mCherry positive, 1 YFP positive and 1 double positive, there are in total 2 mCherry positive, 2 YFP positive and 1 double positive spores. Subtracting 1 (double positive) from each of the mCherry and YFP positive spores yields the correct number of spores of each type. We then counted all the spores that fell into the parental ditype (PD) (2 mCherry positive, 2 YFP positive), non-parental ditype (NPD) (2 double positive) and tetratype (TT) (1 mCherry positive, 1 YFP positive, 1 double positive) tetrads. For each, we then calculated the percent of asci that were classified as each. Our unbiased approach identified PD, NPD and TT at the expected (1:1:4) frequencies. The analysis of the data can be found at the Stowers Original Data Repository (http://www.stowers.org/research/publications/libpb-1270).

## Supporting information

S1 Fig Drive phenotypes of *Sp*/*Sk* mosaic chromosomes suggest the existence of a driver in *Sp*. Cartoons of the driving-mosaic chromosomes. % transmission refers to transmission of the mosaic chromosome into spores of mosaic/*Sk* heterozygotes homozygous for *rec12*Δ. *rec12*Δ mutants fail to initiate meiotic recombination, so chromosomes get transmitted whole through meiosis. *Sp* DNA is depicted in blue, *Sk* DNA in red. The numbers under the chromosomes are the breakpoints between the *Sp* and *Sk* regions. These data were published in [7]. All driving chromosomes show the *Sp* DNA region between 1,397,380 and 1,810,659 which includes *Sp wtf13*. All driving chromosomes also share *Sk* DNA between 252,650–763,460 and after 1,810,859. The second region includes the *wtf18* locus, so all the driving chromosomes have *Sk wtf18*.
(TIF)

S2 Fig Comparison of Wtf proteins. *Sk* allele names are depicted in red. *Sp* allele names are depicted in blue. (A) Alignment of the long isoforms of *Sk* Wtf4 and *Sp* Wtf13. *Sk* Wtf4 and *Sp* Wtf13 share 78% amino acid identity (left). Both genes have two transcriptional and translational start sites (right). (B) Alignment of the long isoform of *Sp* Wtf13 and *Sk* Wtf13 (83% amino acid identity) (left). *Sk wtf13* has only one transcriptional and translational start site (right). (C) Alignment of the proteins encoded by the *Sp wtf18* allele present in the reference genome and the *Sk wtf18* allele (97% identity) (left). Both *wtf18* genes have one transcriptional and translational start site (right). (D) Alignment of the Wtf18 protein from *Sk* and the Wtf18-2 protein from *Sp*. *Sk* Wtf18 and *Sp* Wtf18-2 share 98% amino acid identity. M’s represent the translational start sites.
(TIF)

S3 Fig Correcting gene annotations with long-read RNA sequencing data. Alignment of long-read RNA sequencing data from Kuang et al to *Sp wtf13* (A) and *Sp wtf18* (B) [41]. Our annotation of the genes is shown on top (grey) and the PomBase annotation is shown below (blue). M’s represent the translational start sites. In red and orange, are the meiotic transcripts sequenced by [41]. Only transcripts with 11 reads or more are depicted. If the transcript had more than 50 reads, the transcript is shown in red. The data were visualized using IGV (http://www.broadinstitute.org/igv). Exons are depicted as thick boxes with white arrows, introns as thin lines with blue arrows, and the UTRs as thin lines. No transcripts with more than 11 reads were reported at six hours for *Sp wtf18*. The PomBase gene model for *Sp wtf13* and *wtf18* is different than the one suggested by the RNA sequencing data [59]. Our annotation predictions are consistent with those of [8] which were made computationally. We use the gene models suggested by the data (grey).
(TIF)

S4 Fig Propidium iodide staining to detect loss of membrane integrity. Representative image (top) and quantification (bottom) of PI stained tetrads from the indicated diploids. (A) *Sp wtf13*/*ade6+* (n = 58 4-spore asci). (B) empty vector/*ade6+* (n = 79 4-spore asci). (C) *Sp wtf13*^*antidote*^/*ade6+* (n = 123 4-spore asci). (D) *Sp wtf18-2*/*ade6+* (n = 116 4-spore asci). The images were smoothed using Gaussian blur. The scale bar represents 3μm. The pattern of spore death produced by *Sp wtf13/ade6+*, *Sp wtf13*^*antidote*^*/ade6+* and *Sp wtf18-2/ade6+* diploids were all significantly different from that of the empty vector/*ade6+* control diploids (G-test, p-value< 0.0001).
(TIF)

S5 Fig Alignment of Wtf proteins. *Sk* allele names are depicted in red. *Sp* allele names are depicted in blue. Alignment of *Sp* Wtf13 (long isoform), *Sp* Wtf18, *Sp* Wtf18-2 and *Sk* Wtf18.
(TIF)

S6 Fig Residues encoded in exon 6 are important for determining specificity between drivers and suppressors. Allele transmission and fertility of *Sk* diploids with the indicated *Sp wtf13 and Sp wtf18* mutant alleles integrated at the *ade6* marker locus. Cartoons depicting the mutations made at the C-termini of the proteins are shown. Alleles were followed using drug resistance markers (*kanMX4* or *hphMX6*). Diploids 1, 6 and 7 are repeated from Fig 2. Diploids 6, 7, 24 and 25 were compared to diploid 1 to detect suppression of the drive phenotype of *Sp wtf13* and differences in fertility as measured by viable spore yield. Diploids 27–31 were compared to diploid 26 as control to detect if these alleles could suppress the drive phenotype of *Sp wtf13*^*rep*Δ^. * indicates p-value of < 0.05 (G-test [allele transmission] and Wilcoxon test [fertility]). Fertility was normalized to the empty-vector control (Fig 2, diploid 10) and reported as percent. More than 200 viable haploid spores were scored for each cross. Spores that inherited both markers and were thus hygromycin^R^ Ade+ or hygromycin^R^ geneticin^R^ were excluded from the analyses. Raw data can be found in S1 and S2 Tables. The yellow boxes are the repeat units found at the C-terminus. The *wtf18-2*^*mut1*^ allele is *wtf18-2* (D366N). The *Sp wtf18-2*^*mut2*^ allele is *Sp wtf18-2* (G370E, M372T, D373E, V374A). For the *Sp wtf18*^*+rep*^ allele, the first repeat unit (LGNAFGG) was inserted between residues 332 and 333. In both *Sp wtf13*^*rep*Δ^ and *wtf18-2*^*rep*Δ^, the first repeat unit was deleted. The *wtf18* allele from *Sk* is shown in red (contains three repeats units; the first yellow box is depicted with a brown line to indicate a different residue in the first unit compared to *wtf13*^*rep*Δ^, see S5 Fig).
(TIF)

S7 Fig *Sp wtf18-2*, but not *Sp wtf18*, is a suppressor of the *Sp wtf13* meiotic driver. Using two different double heterozygotes, we showed that *Sp wtf18* from the reference genome is unable to suppress drive from *Sp wtf13*. The first double heterozygotes (top) included *wtf13+ wtf18+* on one haplotype and *wtf13*Δ *wtf18-2+* on the other. In these diploids, the *wtf13*Δ spores are expected to also inherit the *wtf18-2* suppressor 72% of the time due to linkage (28 cM) between the loci. The second diploids (bottom) we tested had *wtf13+ wtf18-2+* on one haplotype and *wtf13*Δ *wtf18+* on the other. In these diploids, the *wtf13*Δ spores are expected to inherit the *wtf18* allele 72% of the time. The table shows the expected results if both alleles of *wtf18* are suppressors and if only *wtf18-2* is a suppressor. The expected results are based on the assumption that the suppressor only rescues the spores which inherit the suppressor (demonstrated in Fig 4B). The observed results fit the model in which only *wtf18-2* is a suppressor of *wtf13*. The *wtf13*Δ allele was followed using the *hphMX6* drug resistance cassette.
(TIF)

S8 Fig *Sp* Wtf13-YFP is enriched in the two surviving spores of a *Sp wtf13-YFP/lys4+* heterozygote. Example of the localization of Wtf13-YFP (green). Propidium iodide (PI) was used to detect spores that have lost membrane integrity. *Sp wtf13-YFP* transgene was integrated at *lys4* in *Sk*. The drive phenotype of this allele is indistinguishable from the untagged *Sp wtf13* allele (Data in S1 Table, compare diploid 34 to diploid 1). This allele exhibit the same localization as the weaker *Sp wtf13-YFP* allele shown in Fig 5B. The images were smoothed using Gaussian blur. The scale bar represents 3μm.
(TIF)

S9 Fig Linear unmixing of *Sp* mCh-Wtf18-2 and *Sp* Wtf13-YFP in the spore sac. (A) Linear unmixing (see Materials and Methods) of the representative image presented in Fig 5C is shown. *mCh-wtf18-2* was integrated at *lys4* in *Sk*. (B) Linear unmixing of the representative image in Fig 6B is shown. The transgene constructs were both integrated at *lys4* in opposite haplotypes. (C) Linear unmixing of the representative image in Fig 6D is shown. *wtf13-YFP* was integrated at *ade6* in *Sk*. *mCh-wtf18-2* was integrated at *lys4* in *Sk*. The autofluorescence images are shown with the same intensity as their respective channels. The brightness and contrast were adjusted differently for images A, B and C and the images were smoothed using Gaussian blur. Scale bar represents 3μm.
(TIF)

S10 Fig *Sp* mCh-Wtf18-2 and *Sp* Wtf13-YFP co-localize. (A) The cartooned diploid was generated by integrating *Sp wtf13-YFP* at *ade6* and *Sp mCh-wtf18-2* at *lys4* in *Sk*. The *ade6* and *lys4* loci are unlinked, thus the alleles will segregate randomly. This diploid will generate: parental ditype (PD), non-parental ditype (NPD) and tetratype (TT) tetrads. Due to random assortment, we expect PD, NPD and TT at a 1:1:4 ratio. Consistent with this, we determined that there were 15% PD, 17% NPD and 68% TT tetrads (See Material and Methods). (B) Examples of the localization of mCh-Wtf18-2 (magenta) and Wtf13-YFP (green) in representatives of the three classes of tetrads are shown. The brightness and contrast were adjusted differently for each image and images were smoothed using Gaussian blur. The scale bar represents 3μm.
(TIF)

S11 Fig *Sp* mCh-Wtf18-2 and *Sp* Wtf13-YFP co-localize. Examples of the localization of mCh-Wtf18-2 (magenta) and Wtf13-YFP (green) in non-parental ditype tetrads from diploids heterozygous for *Sp mCh-wtf18-2* and *Sp wtf13-YFP*. The images were processed to remove autofluorescence using linear unmixing, scaled to the same size, and smoothed using Gaussian blur. The brightness and contrast were adjusted differently for each image. The NPD tetrads from Fig 6D and S9C Fig as well as S10B Fig are also represented in this Supplemental Figure for easy comparison. The scale bar represents 2μm.
(TIF)

S12 Fig Predicted transmembrane domains of Wtf proteins. Plots show the probabilities of inside, outside and transmembrane helices in (A) Wtf13^antidote^, (B) Wtf18-2, (C) Wtf18, (D) Wtf13^repΔ^, (E) Wtf18-2^repΔ^ and (F) Wtf18^+rep^. Outside is depicted in cyan, inside in magenta and the transmembrane helices in black. At the top of every plot there is the prediction of the transmembrane protein topology.
(TIF)

S1 Table Raw data of allele transmission from Figs 2 and 3. Each of the horizontal lines represents the relevant genotype and allele transmission of the indicated diploid. The first column represents the diploid number, which matches the numbers in Fig 2 and Fig 3. In columns 2–5, the strain number (SZY) and relevant genotype of the haploid parent strains used to determine the allele transmission at the drive locus (*ade6* or *lys4*). *Sp* alleles are labeled in blue. *Sk* alleles are labeled in red. Columns 6 and 7 indicate which phenotypes were followed at the control locus (*ura4*) and the number of progeny that showed the indicated phenotype. Columns 9 and 10 indicate the phenotypes that were followed at the drive loci (*ade6* or *lys4*) and the number of haploid progeny that exhibited the indicated phenotype. Some of the progeny inherited both markers from the parent strains. The number of those progeny (geneticin^R^ Ade+, hygromycin^R^ Ade+ or hygromycin^R^ geneticin^R^) is presented in column 11 and the percent of the progeny with this phenotype is shown in column 12. Column 13 shows the fraction of the haploid progeny that inherited the genotype of allele 1. Column 14 shows the fraction of the haploid progeny that inherited the genotype of allele 2. Column 15 shows the total progeny assayed excluding progeny that inherited both markers. Column 16 shows the total progeny including progeny that inherited both markers. Column 17 shows the total number of diploids assayed. Last column shows the p-value calculated by comparing diploids 1–9, 21–23 to control diploid 10 using a G-test. Diploids 11–13, 24 and 25 were compared to diploid 1 using a G-test. Diploids 27–31 were compared to control diploid 26.
(PDF)

S2 Table Raw data for fertility from Figs 2, 3 and 4. Each of the rows represents the diploid assayed, which matches the diploid number in Figs 2, 3 and 4. The numbers underneath the diploid number are SZY numbers of the haploid parent strains. All the viable spore yield values are shown for each diploid. Diploids 1–9, 11, 12, 13, 21, 22–31 were normalized to control diploid 10. Diploids 14–19 were normalized to diploid 20. To determine any fertility defect in Fig 2, we compared diploids 1–9, 21, 22 and 23 to control diploid 10. To test if the *wtf18* alleles rescued the fertility phenotype caused by the *wtf13* driver, we compared diploid 6, 7, 11, 12 and 13 from Fig 3, and diploids 24 and 25 from S6 Fig to diploid 1. In S6 Fig, diploids 27–31 were compared to diploid 26 as control. Diploids in Fig 4 were all compared to diploid 20. We calculated the p-value for each diploid using the Wilcoxon test.
(PDF)

S3 Table Raw data of allele transmission from Fig 4. Each of the horizontal lines represents the relevant genotype and allele transmission of the indicated diploid. The first column represents the diploid number, which matches the numbers in Fig 4. In columns 2–5, the strain number (SZY) and relevant genotype of the haploid parent strains used to determine the allele transmission at the *Sp wtf13* or *Sp wtf18-2* loci. Columns 6 and 7 indicate which phenotypes were followed at the control locus (*ura4*) and the number of progeny that showed the indicated phenotype. Columns 9 and 10 indicate the phenotypes that were followed at the *Sp wtf13* or *Sp wtf18-2* loci (bolded) and the number of haploid progeny that exhibited the indicated phenotype. Column 11 shows the fraction of the haploid progeny that inherited the genotype of allele 1. Column 12 shows the fraction of the haploid progeny that inherited the genotype of allele 2. Column 13 shows the total progeny assayed. Column 14 shows the total number of diploids assayed. Last column shows the p-value calculated by comparing diploids 16 and 17 to control diploid 14, and diploids 18 and 19 to control diploid 15. P-values were calculated using a G-test.
(PDF)

S4 Table Raw data for Fig 4B and *mCh-wtf18-2* allele. Each of the horizontal lines represents the relevant genotype and allele transmission of the indicated diploid. The first column shows the Fig where these data were represented, or the diploid number referred to in the text. In columns 2–5, the strain number (SZY) and relevant genotype of the haploid parent strains used to determine the allele transmission are shown. Diploids with the indicated genotype will generate four different types of spores (column 6–9). Columns 6 and 7 are the parental classes and columns 8 and 9 are the recombinant classes. Column 10 shows the total progeny assayed. Column 11 shows the total number of diploids assayed.
(PDF)

S5 Table Plasmids used in this study. (PDF)

S6 Table Oligos used in this study. (PDF)

S7 Table Yeast Strains used in this study. Notes: The *ura4-X* allele in SZY562 and SZY581 listed in the table is either *ura4-D18* or *ura4-294*. *Sp wtf13* (M1X, M12X) is the *Sp wtf13* poison allele. *Sp wtf18-2* (D366N) is the *Sp wtf18-2*^*mut1*^ allele used in S6 Fig. The *Sp wtf18-2* (G370E, M372T, D373E, V374A) allele is *Sp wtf18-2*^*mut2*^ in S6 Fig. The *Sp wtf18-2* (p.333-339Δ) allele contains the first repeat unit deleted. *Sp wtf18* (G332_I333insLGNAFGG) is the *wtf18*^*+rep*^ allele in which we inserted the first repeat unit between residues 332 and 333. *Sp wtf18-2* (D366N, D366_I367insN) is the *Sp wtf18-2*^*D366NN*^ allele used in Fig 3. *Sp wtf13* (p.343-349Δ) is an allele containing the deletion of the first repeat unit. We are listing the mutations in the protein. *Sp wtf13* (358A>T, 359T>A, 360G>C) is the *Sp wtf13* antidote allele. We are listing the mutations in the nucleotide sequence.
(PDF)
