# Supplementary figures and images for: A suppressor of a wtf poison-antidote meiotic driver acts via mimicry of the driver’s antidote
Source: PLoS Genet. 2018 Nov 26;14(11):e1007836. doi: 10.1371/journal.pgen.1007836 (PMC6283613; doi:10.1371/journal.pgen.1007836)

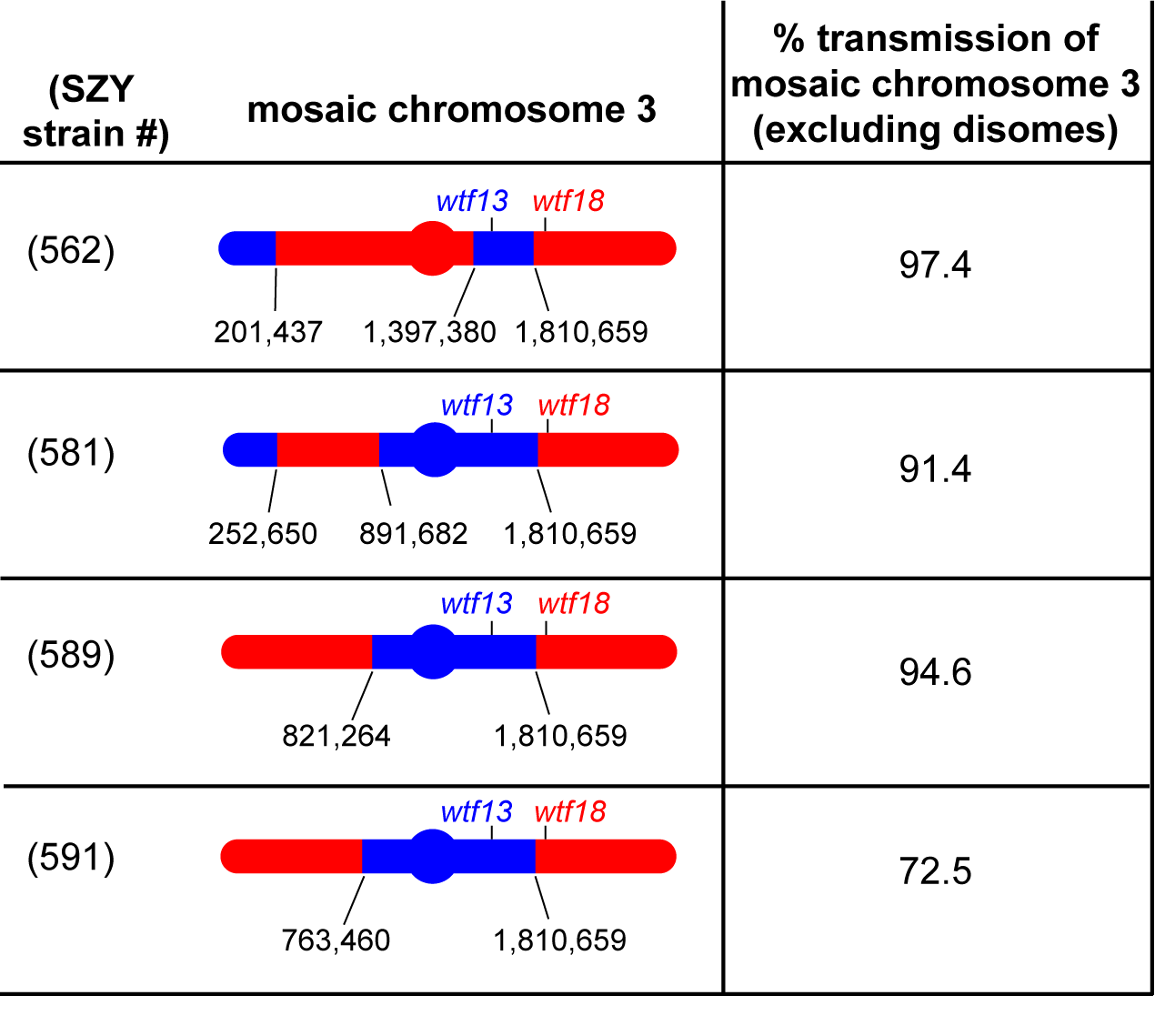

Supplement: S1 Fig — Cartoons of the driving-mosaic chromosomes. % transmission refers to transmission of the mosaic chromosome into spores of mosaic/Sk heterozygotes homozygous for rec12Δ. rec12Δ mutants fail to initiate meiotic recombination, so chromosomes get transmitted whole through meiosis. Sp DNA is depicted in blue, Sk DNA in red. The numbers under the chromosomes are the breakpoints between the Sp and Sk regions. These data were published in [7]. All driving chromosomes show the Sp DNA region between 1,397,380 and 1,810,659 which includes Sp wtf13. All driving chromosomes also share Sk DNA between 252,650–763,460 and after 1,810,859. The second region includes the wtf18 locus, so all the driving chromosomes have Sk wtf18. (TIF) [file pgen.1007836.s001.tif]

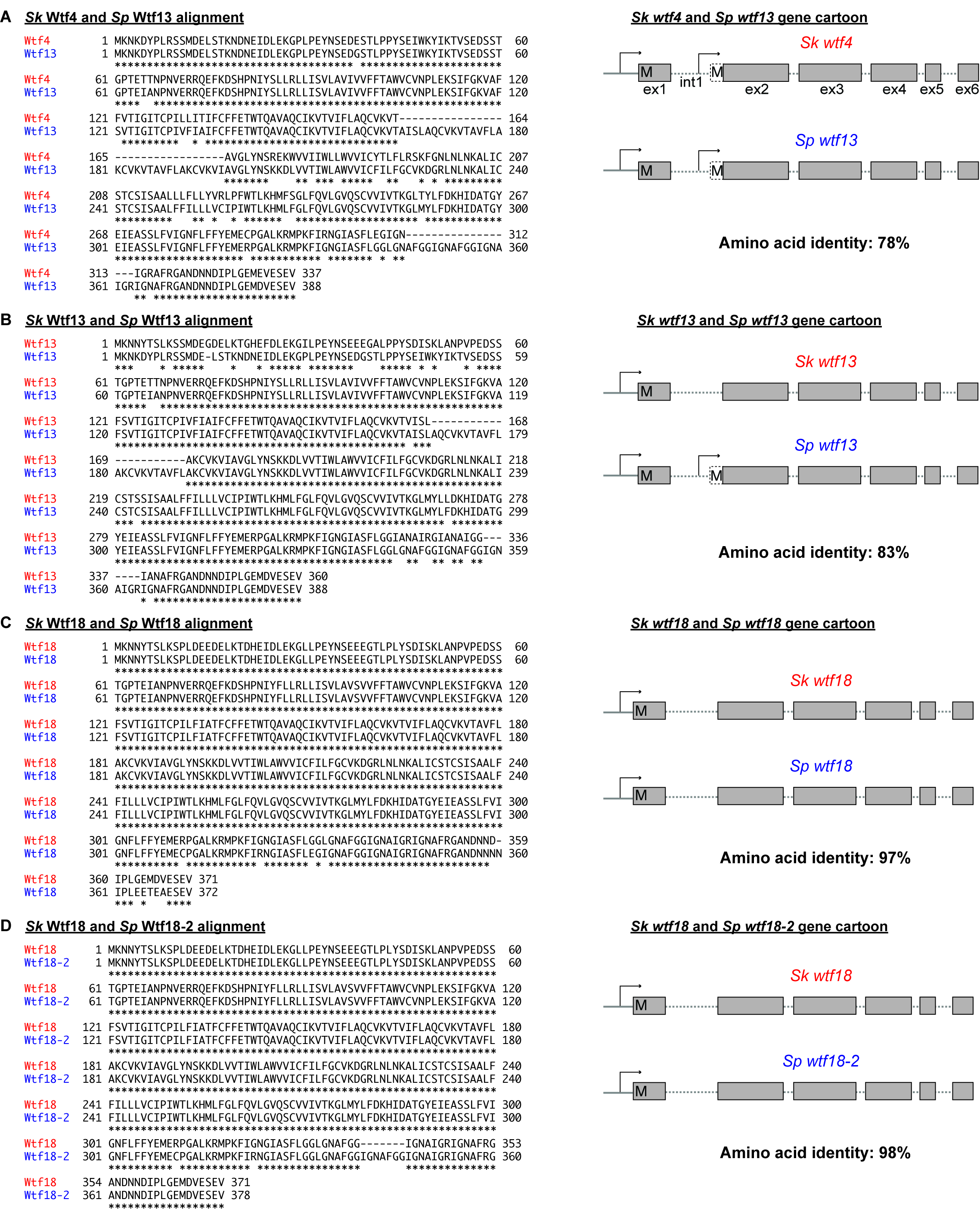

Supplement: S2 Fig — Sk allele names are depicted in red. Sp allele names are depicted in blue. (A) Alignment of the long isoforms of Sk Wtf4 and Sp Wtf13. Sk Wtf4 and Sp Wtf13 share 78% amino acid identity (left). Both genes have two transcriptional and translational start sites (right). (B) Alignment of the long isoform of Sp Wtf13 and Sk Wtf13 (83% amino acid identity) (left). Sk wtf13 has only one transcriptional and translational start site (right). (C) Alignment of the proteins encoded by the Sp wtf18 allele present in the reference genome and the Sk wtf18 allele (97% identity) (left). Both wtf18 genes have one transcriptional and translational start site (right). (D) Alignment of the Wtf18 protein from Sk and the Wtf18-2 protein from Sp. Sk Wtf18 and Sp Wtf18-2 share 98% amino acid identity. M’s represent the translational start sites. (TIF) [file pgen.1007836.s002.tif]

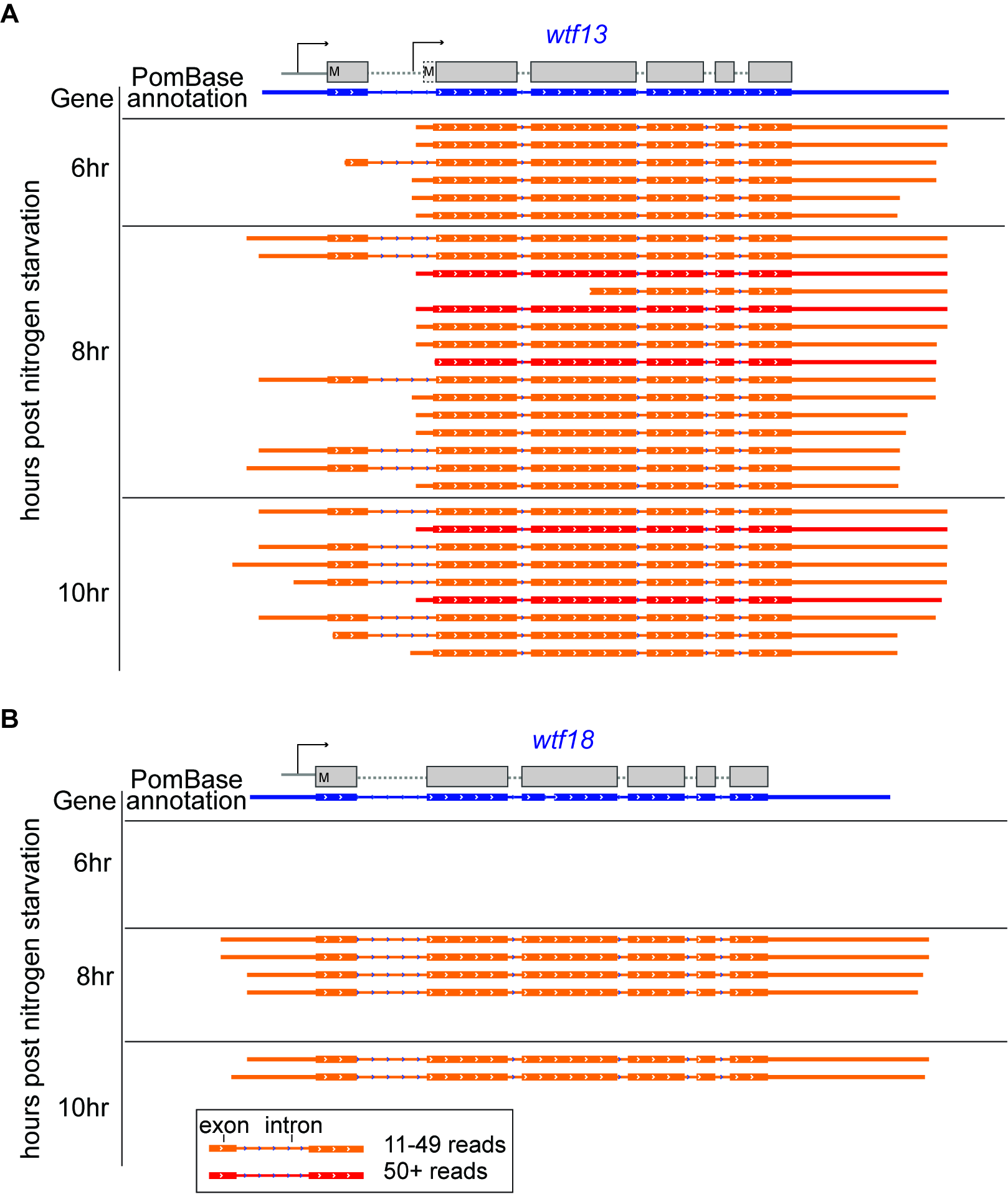

Supplement: S3 Fig — Alignment of long-read RNA sequencing data from Kuang et al to Sp wtf13 (A) and Sp wtf18 (B) [41]. Our annotation of the genes is shown on top (grey) and the PomBase annotation is shown below (blue). M’s represent the translational start sites. In red and orange, are the meiotic transcripts sequenced by [41]. Only transcripts with 11 reads or more are depicted. If the transcript had more than 50 reads, the transcript is shown in red. The data were visualized using IGV (http://www.broadinstitute.org/igv). Exons are depicted as thick boxes with white arrows, introns as thin lines with blue arrows, and the UTRs as thin lines. No transcripts with more than 11 reads were reported at six hours for Sp wtf18. The PomBase gene model for Sp wtf13 and wtf18 is different than the one suggested by the RNA sequencing data [59]. Our annotation predictions are consistent with those of [8] which were made computationally. We use the gene models suggested by the data (grey). (TIF) [file pgen.1007836.s003.tif]

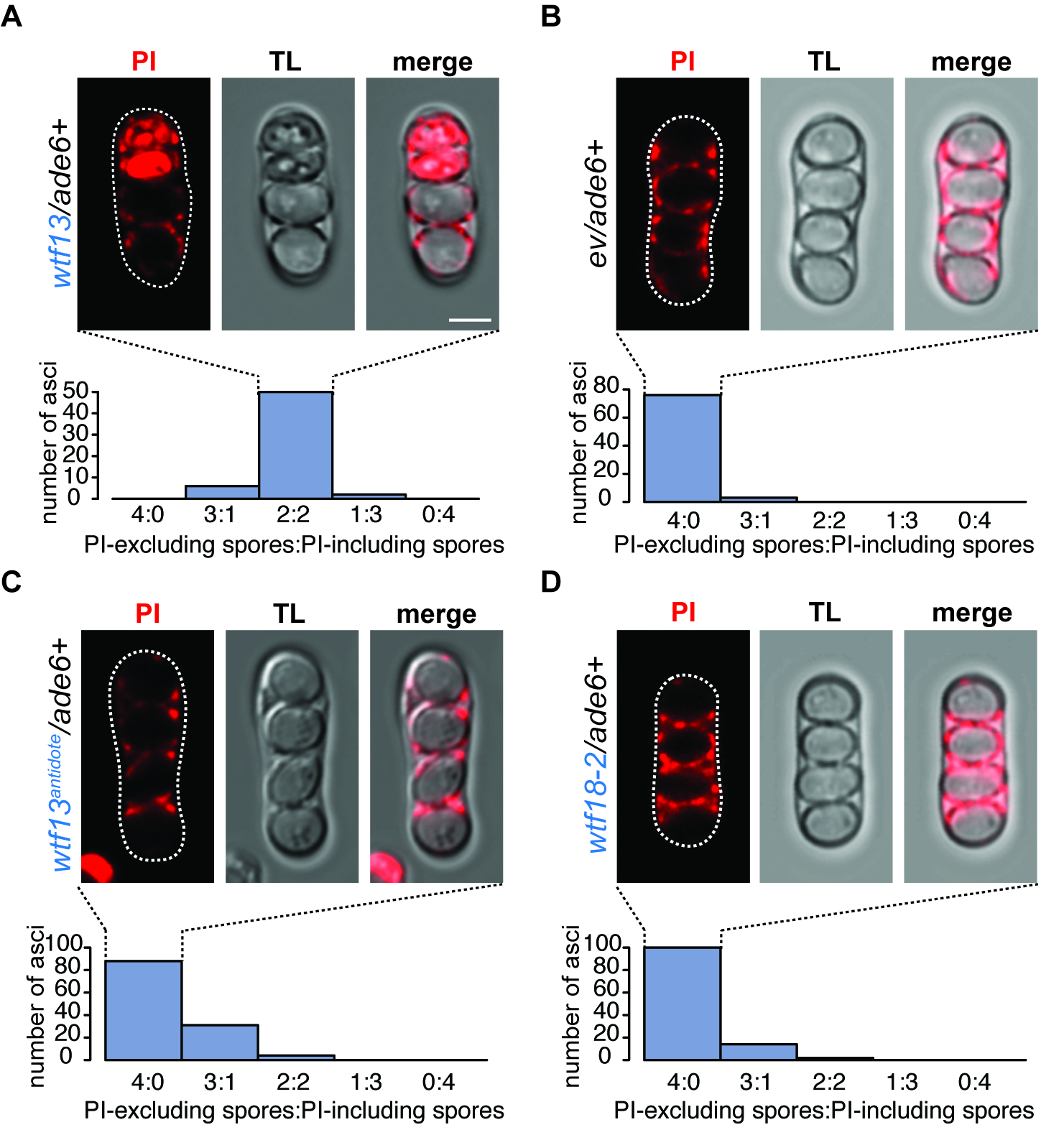

Supplement: S4 Fig — Representative image (top) and quantification (bottom) of PI stained tetrads from the indicated diploids. (A) Sp wtf13/ade6+ (n = 58 4-spore asci). (B) empty vector/ade6+ (n = 79 4-spore asci). (C) Sp wtf13antidote/ade6+ (n = 123 4-spore asci). (D) Sp wtf18-2/ade6+ (n = 116 4-spore asci). The images were smoothed using Gaussian blur. The scale bar represents 3μm. The pattern of spore death produced by Sp wtf13/ade6+, Sp wtf13antidote/ade6+ and Sp wtf18-2/ade6+ diploids were all significantly different from that of the empty vector/ade6+ control diploids (G-test, p-value< 0.0001). (TIF) [file pgen.1007836.s004.tif]

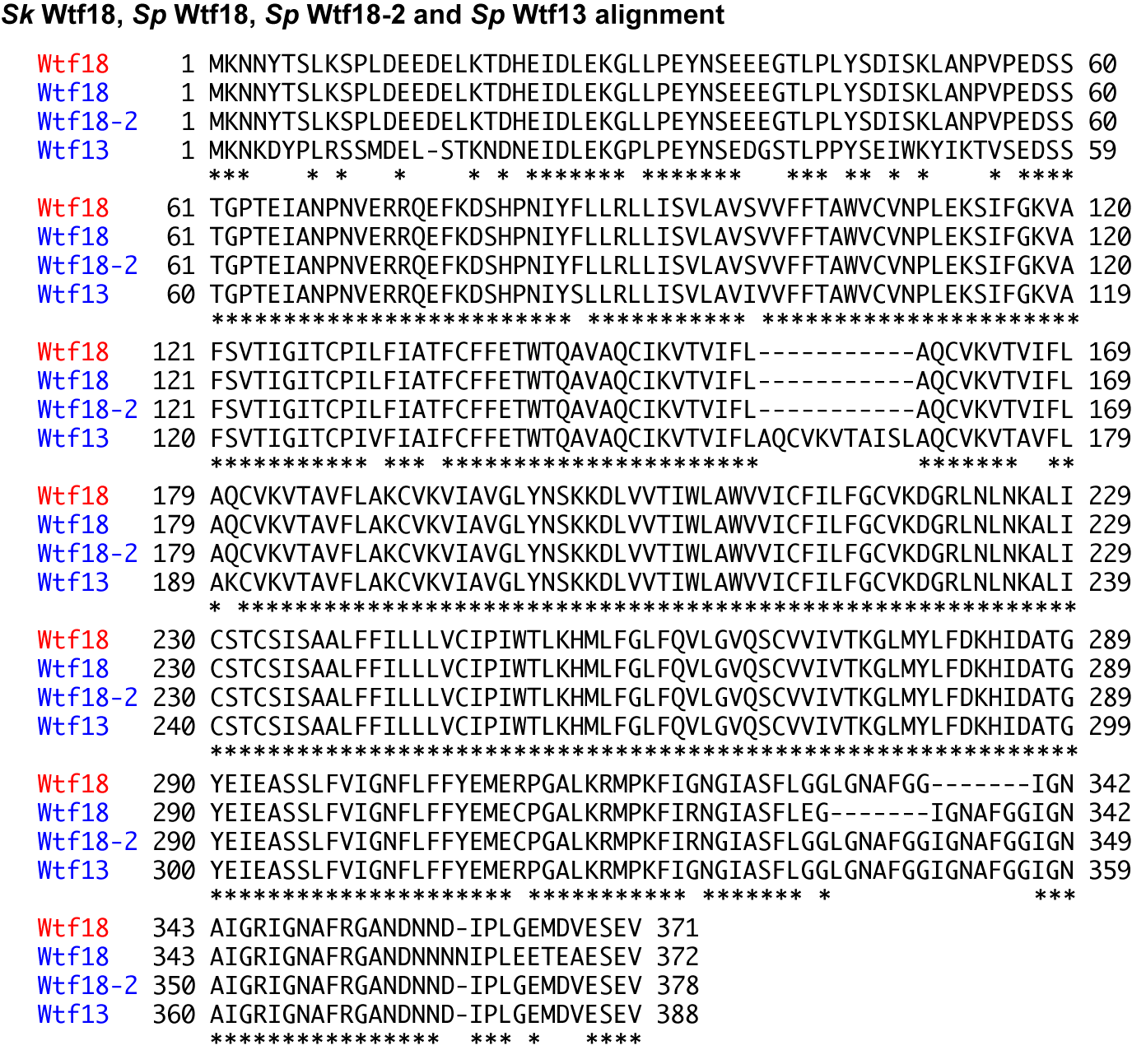

Supplement: S5 Fig — Sk allele names are depicted in red. Sp allele names are depicted in blue. Alignment of Sp Wtf13 (long isoform), Sp Wtf18, Sp Wtf18-2 and Sk Wtf18. (TIF) [file pgen.1007836.s005.tif]

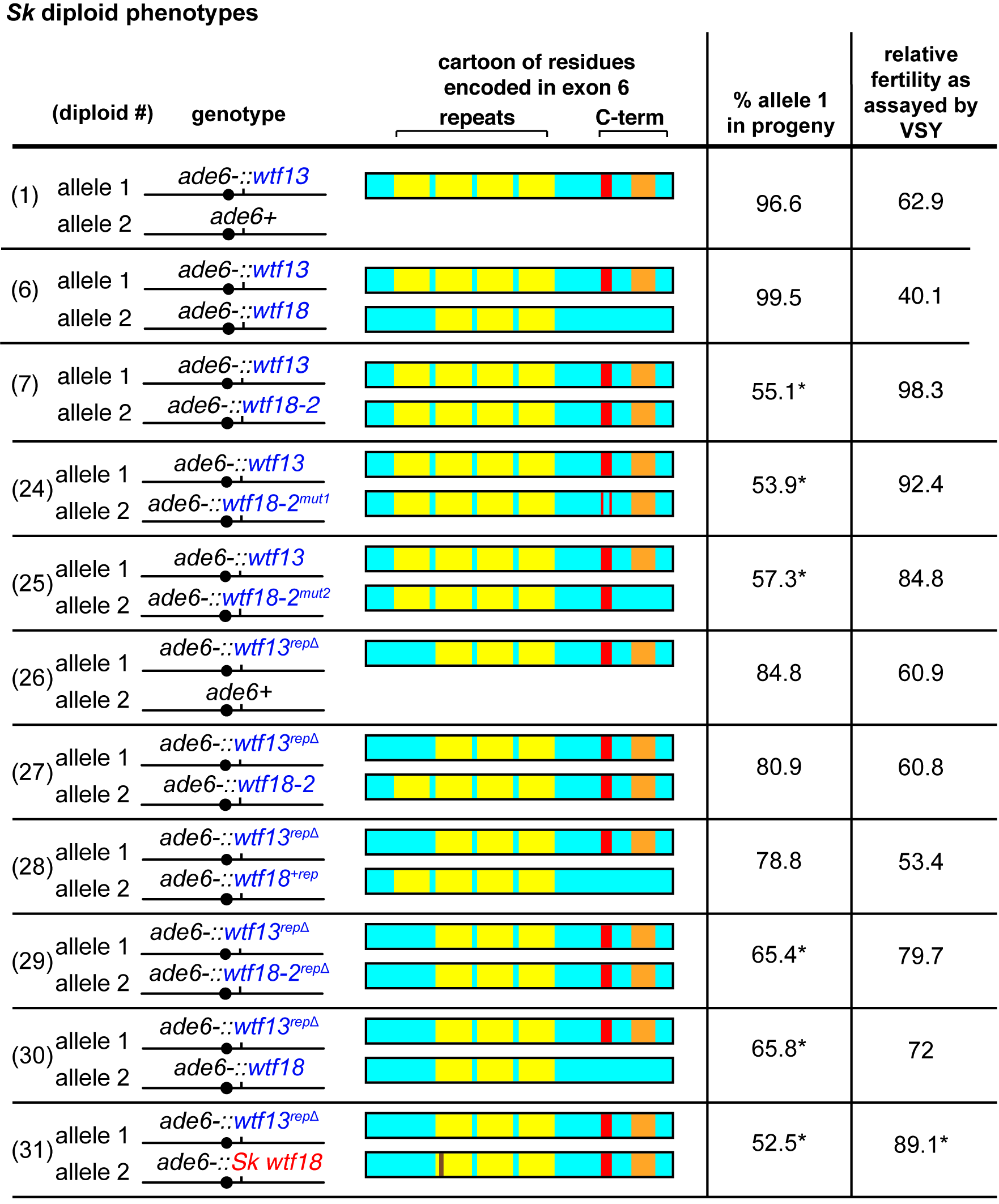

Supplement: S6 Fig — Allele transmission and fertility of Sk diploids with the indicated Sp wtf13 and Sp wtf18 mutant alleles integrated at the ade6 marker locus. Cartoons depicting the mutations made at the C-termini of the proteins are shown. Alleles were followed using drug resistance markers (kanMX4 or hphMX6). Diploids 1, 6 and 7 are repeated from Fig 2. Diploids 6, 7, 24 and 25 were compared to diploid 1 to detect suppression of the drive phenotype of Sp wtf13 and differences in fertility as measured by viable spore yield. Diploids 27–31 were compared to diploid 26 as control to detect if these alleles could suppress the drive phenotype of Sp wtf13repΔ. * indicates p-value of < 0.05 (G-test [allele transmission] and Wilcoxon test [fertility]). Fertility was normalized to the empty-vector control (Fig 2, diploid 10) and reported as percent. More than 200 viable haploid spores were scored for each cross. Spores that inherited both markers and were thus hygromycinR Ade+ or hygromycinR geneticinR were excluded from the analyses. Raw data can be found in S1 and S2 Tables. The yellow boxes are the repeat units found at the C-terminus. The wtf18-2mut1 allele is wtf18-2 (D366N). The Sp wtf18-2mut2 allele is Sp wtf18-2 (G370E, M372T, D373E, V374A). For the Sp wtf18+rep allele, the first repeat unit (LGNAFGG) was inserted between residues 332 and 333. In both Sp wtf13repΔ and wtf18-2repΔ, the first repeat unit was deleted. The wtf18 allele from Sk is shown in red (contains three repeats units; the first yellow box is depicted with a brown line to indicate a different residue in the first unit compared to wtf13repΔ, see S5 Fig). (TIF) [file pgen.1007836.s006.tif]

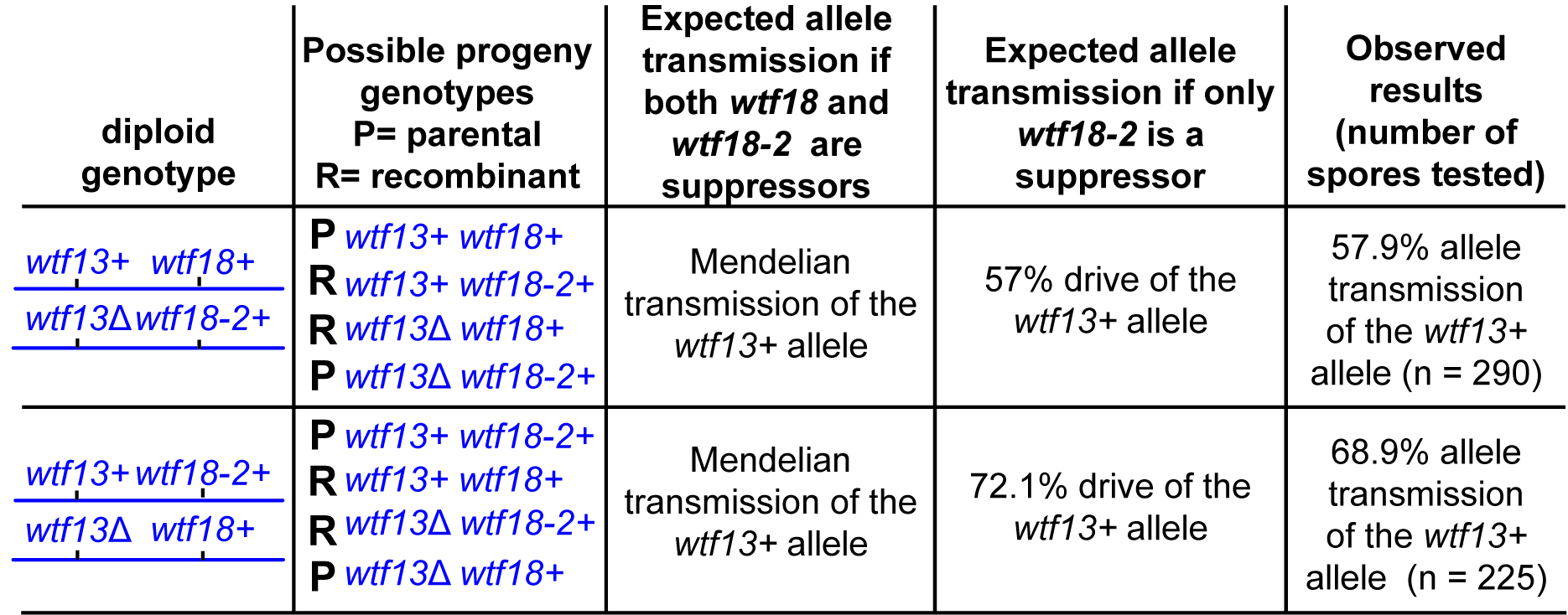

Supplement: S7 Fig — Using two different double heterozygotes, we showed that Sp wtf18 from the reference genome is unable to suppress drive from Sp wtf13. The first double heterozygotes (top) included wtf13+ wtf18+ on one haplotype and wtf13Δ wtf18-2+ on the other. In these diploids, the wtf13Δ spores are expected to also inherit the wtf18-2 suppressor 72% of the time due to linkage (28 cM) between the loci. The second diploids (bottom) we tested had wtf13+ wtf18-2+ on one haplotype and wtf13Δ wtf18+ on the other. In these diploids, the wtf13Δ spores are expected to inherit the wtf18 allele 72% of the time. The table shows the expected results if both alleles of wtf18 are suppressors and if only wtf18-2 is a suppressor. The expected results are based on the assumption that the suppressor only rescues the spores which inherit the suppressor (demonstrated in Fig 4B). The observed results fit the model in which only wtf18-2 is a suppressor of wtf13. The wtf13Δ allele was followed using the hphMX6 drug resistance cassette. (TIF) [file pgen.1007836.s007.tif]

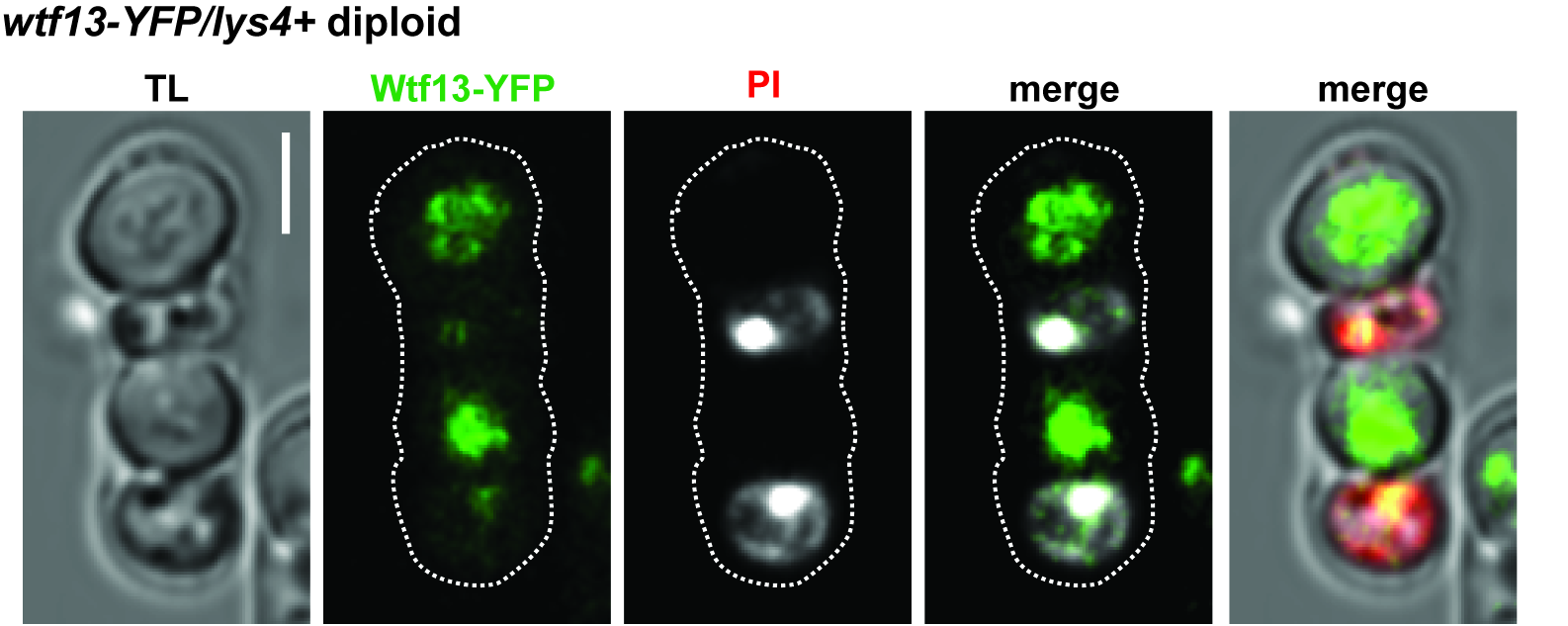

Supplement: S8 Fig — Example of the localization of Wtf13-YFP (green). Propidium iodide (PI) was used to detect spores that have lost membrane integrity. Sp wtf13-YFP transgene was integrated at lys4 in Sk. The drive phenotype of this allele is indistinguishable from the untagged Sp wtf13 allele (Data in S1 Table, compare diploid 34 to diploid 1). This allele exhibit the same localization as the weaker Sp wtf13-YFP allele shown in Fig 5B. The images were smoothed using Gaussian blur. The scale bar represents 3μm. (TIF) [file pgen.1007836.s008.tif]

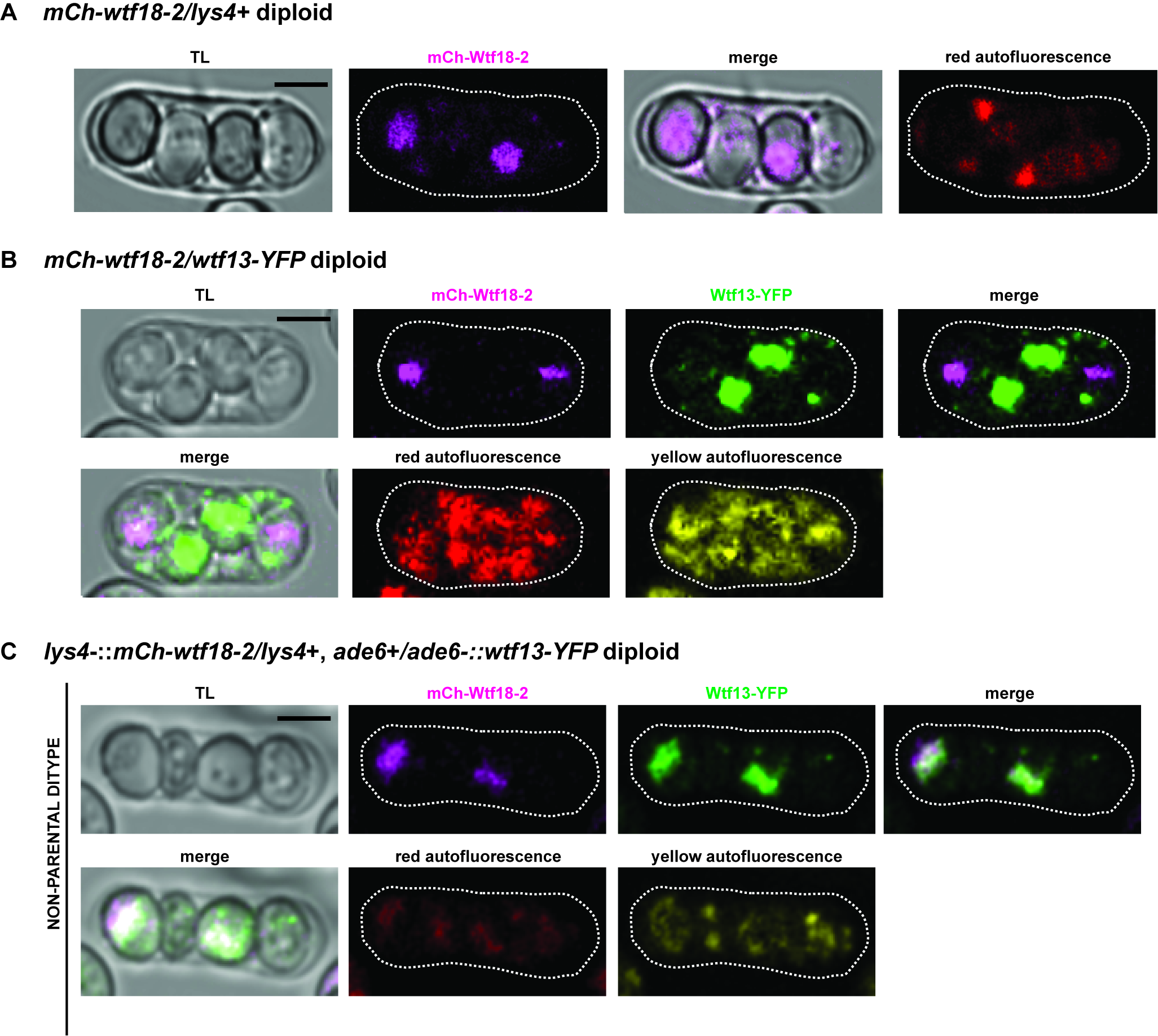

Supplement: S9 Fig — (A) Linear unmixing (see Materials and Methods) of the representative image presented in Fig 5C is shown. mCh-wtf18-2 was integrated at lys4 in Sk. (B) Linear unmixing of the representative image in Fig 6B is shown. The transgene constructs were both integrated at lys4 in opposite haplotypes. (C) Linear unmixing of the representative image in Fig 6D is shown. wtf13-YFP was integrated at ade6 in Sk. mCh-wtf18-2 was integrated at lys4 in Sk. The autofluorescence images are shown with the same intensity as their respective channels. The brightness and contrast were adjusted differently for images A, B and C and the images were smoothed using Gaussian blur. Scale bar represents 3μm. (TIF) [file pgen.1007836.s009.tif]

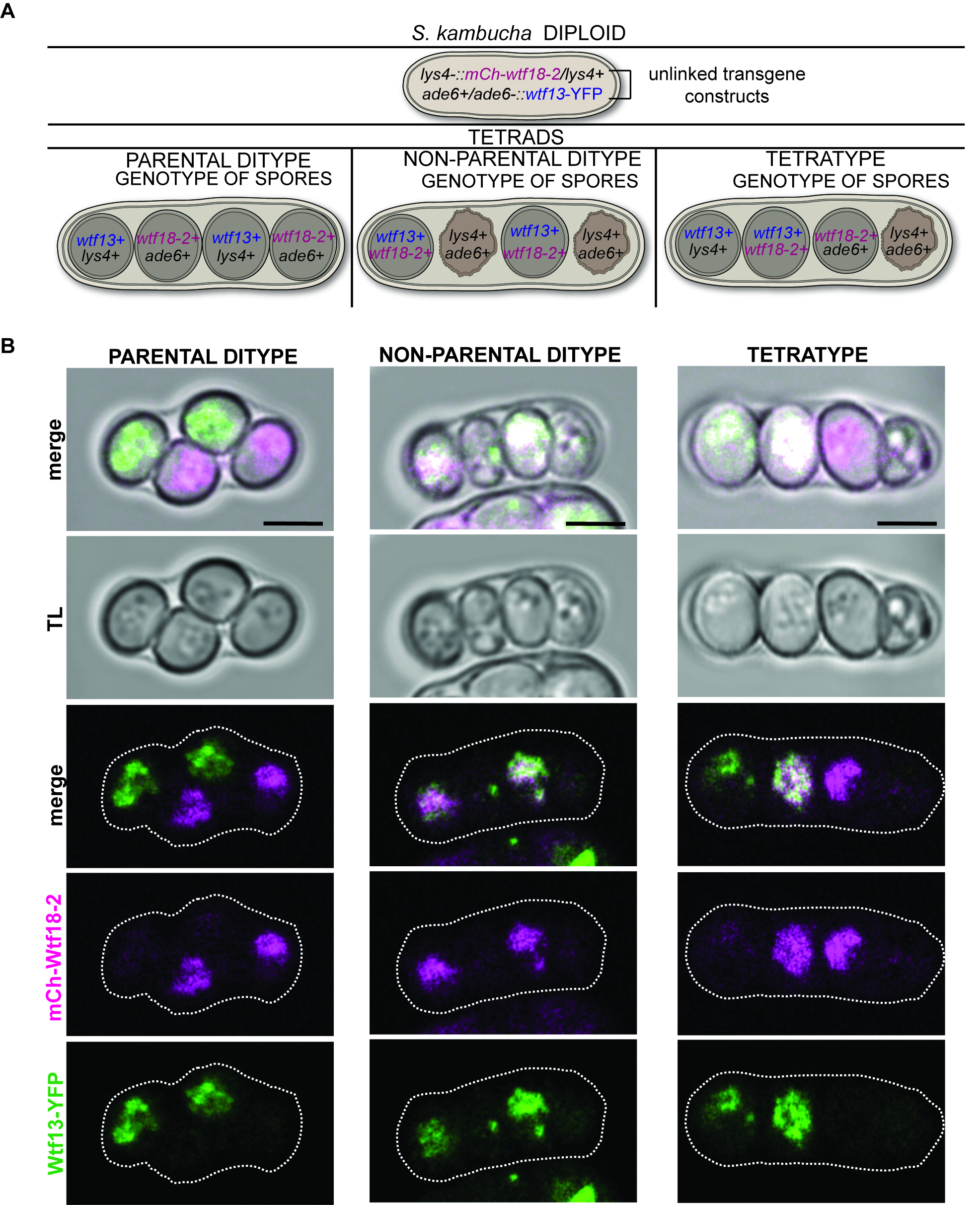

Supplement: S10 Fig — (A) The cartooned diploid was generated by integrating Sp wtf13-YFP at ade6 and Sp mCh-wtf18-2 at lys4 in Sk. The ade6 and lys4 loci are unlinked, thus the alleles will segregate randomly. This diploid will generate: parental ditype (PD), non-parental ditype (NPD) and tetratype (TT) tetrads. Due to random assortment, we expect PD, NPD and TT at a 1:1:4 ratio. Consistent with this, we determined that there were 15% PD, 17% NPD and 68% TT tetrads (See Material and Methods). (B) Examples of the localization of mCh-Wtf18-2 (magenta) and Wtf13-YFP (green) in representatives of the three classes of tetrads are shown. The brightness and contrast were adjusted differently for each image and images were smoothed using Gaussian blur. The scale bar represents 3μm. (TIF) [file pgen.1007836.s010.tif]

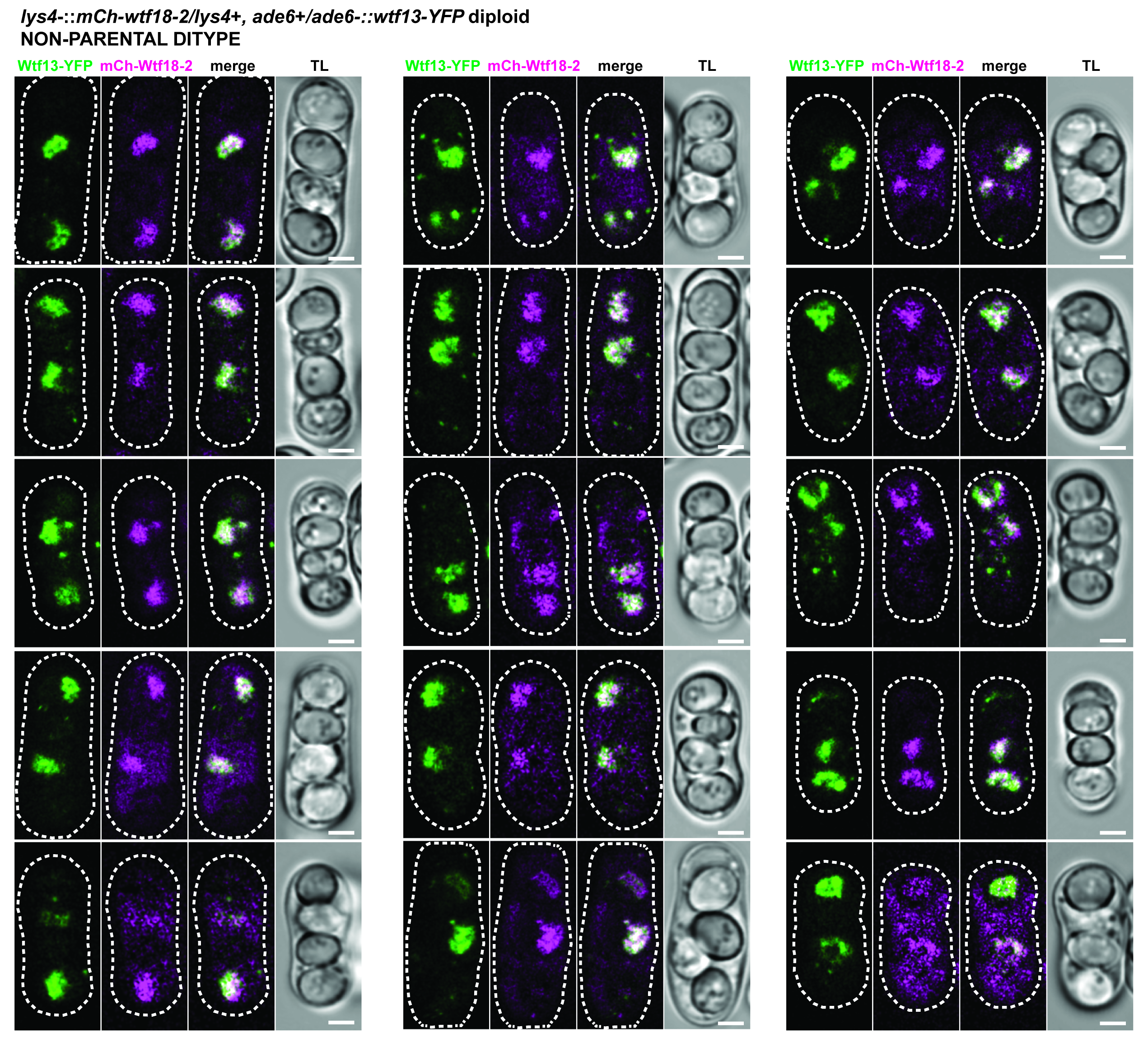

Supplement: S11 Fig — Examples of the localization of mCh-Wtf18-2 (magenta) and Wtf13-YFP (green) in non-parental ditype tetrads from diploids heterozygous for Sp mCh-wtf18-2 and Sp wtf13-YFP. The images were processed to remove autofluorescence using linear unmixing, scaled to the same size, and smoothed using Gaussian blur. The brightness and contrast were adjusted differently for each image. The NPD tetrads from Fig 6D and S9C Fig as well as S10B Fig are also represented in this Supplemental Figure for easy comparison. The scale bar represents 2μm. (TIF) [file pgen.1007836.s011.tif]

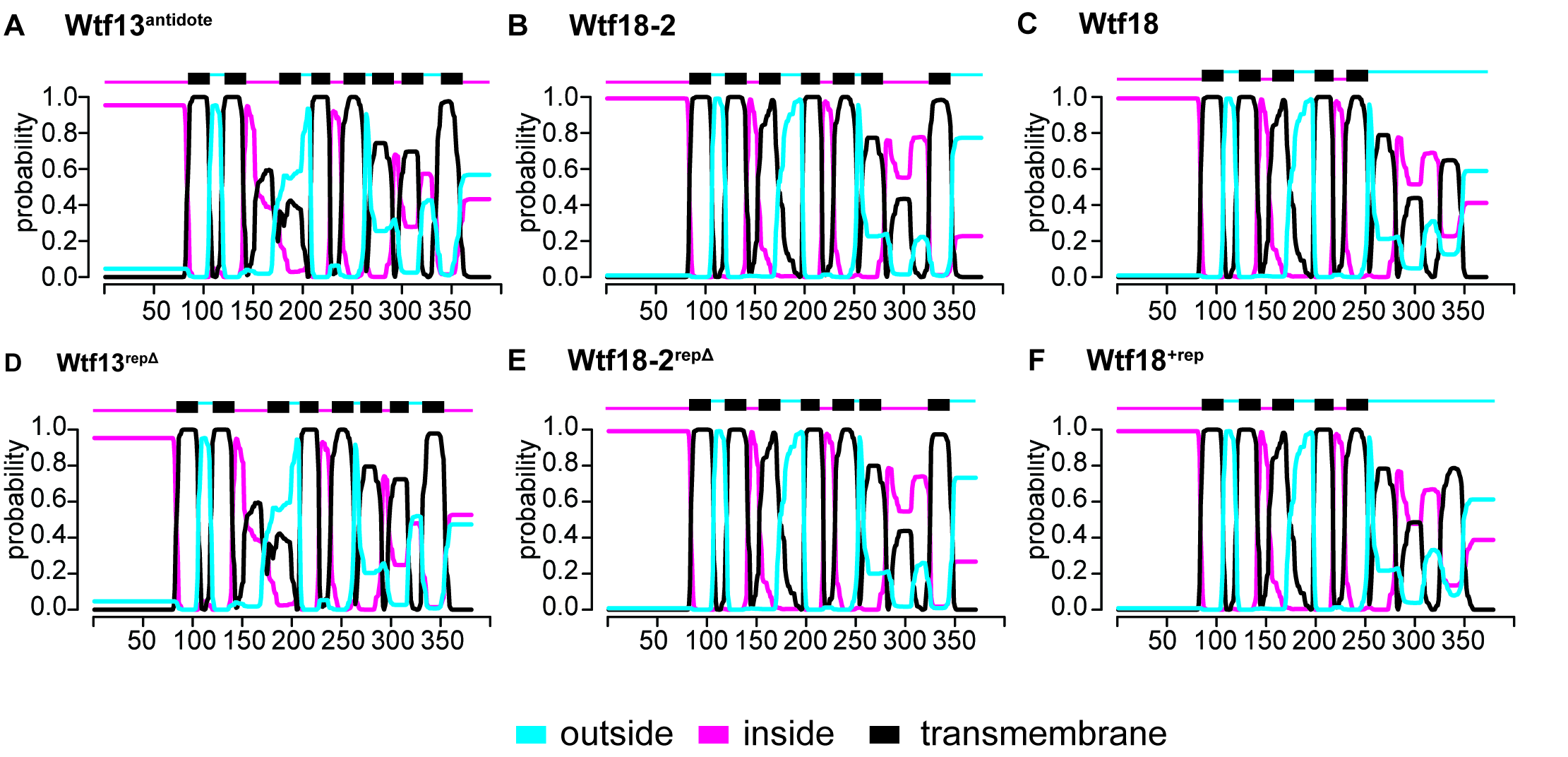

Supplement: S12 Fig — Plots show the probabilities of inside, outside and transmembrane helices in (A) Wtf13antidote, (B) Wtf18-2, (C) Wtf18, (D) Wtf13repΔ, (E) Wtf18-2repΔ and (F) Wtf18+rep. Outside is depicted in cyan, inside in magenta and the transmembrane helices in black. At the top of every plot there is the prediction of the transmembrane protein topology. (TIF) [file pgen.1007836.s012.tif]
