# Supplementary material for: A suppressor of a wtf poison-antidote meiotic driver acts via mimicry of the driver’s antidote
Source: PLoS Genet. 2018 Nov 26;14(11):e1007836. doi: 10.1371/journal.pgen.1007836 (PMC6283613; doi:10.1371/journal.pgen.1007836)
Supplement: S1 Table — Each of the horizontal lines represents the relevant genotype and allele transmission of the indicated diploid. The first column represents the diploid number, which matches the numbers in Fig 2 and Fig 3. In columns 2–5, the strain number (SZY) and relevant genotype of the haploid parent strains used to determine the allele transmission at the drive locus (ade6 or lys4). Sp alleles are labeled in blue. Sk alleles are labeled in red. Columns 6 and 7 indicate which phenotypes were followed at the control locus (ura4) and the number of progeny that showed the indicated phenotype. Columns 9 and 10 indicate the phenotypes that were followed at the drive loci (ade6 or lys4) and the number of haploid progeny that exhibited the indicated phenotype. Some of the progeny inherited both markers from the parent strains. The number of those progeny (geneticinR Ade+, hygromycinR Ade+ or hygromycinR geneticinR) is presented in column 11 and the percent of the progeny with this phenotype is shown in column 12. Column 13 shows the fraction of the haploid progeny that inherited the genotype of allele 1. Column 14 shows the fraction of the haploid progeny that inherited the genotype of allele 2. Column 15 shows the total progeny assayed excluding progeny that inherited both markers. Column 16 shows the total progeny including progeny that inherited both markers. Column 17 shows the total number of diploids assayed. Last column shows the p-value calculated by comparing diploids 1–9, 21–23 to control diploid 10 using a G-test. Diploids 11–13, 24 and 25 were compared to diploid 1 using a G-test. Diploids 27–31 were compared to control diploid 26. (PDF) [file pgen.1007836.s013.pdf]

Diploid #	allele 1 SZY#	GENOTYPE	allele 2 SZY#	GENOTYPE	ura+	ura-	% ura+ (control)	ade+	ade-	ade+ DRUG^R	% ade+ DRUG^R	% allele 1 (excluding ade+ DRUG^R)	% allele 2 (excluding ade+ DRUG^R)	# progeny assayed (w/o ade+ DRUG^R)	# progeny assayed	# diploids assayed	p-value
	1404	ade6-:: Sp wtf13::hphMX6	180	ade6+	173	154	52.9%	13	314	11	3.3	96.02%	3.98	327	338	7	
	1403	ade6-:: Sp wtf13::hphMX6	174	ade6+	89	134	39.9%	6	217	9	3.9	97.31%	2.69	223	232	4	
1					262	288	47.6%	19	531	20	3.5	**96.55%**	3.45	550	570	11	7.95E-09
	**allele 1 SZY#**	**GENOTYPE**	**allele 2 SZY#**	**GENOTYPE**	**ura+**	**ura-**	**% ura+ (control)**	**HYG^R**	**HYG^S**	**HYG^R GEN^R**	**% HYG^R GEN^R**	**% allele 1 (excluding HYG^R GEN^R)**	**% allele 2 (excluding HYG^R GEN^R)**	**# progeny assayed (w/o HYG^R GEN^R)**	**# progeny assayed**	**# diploids assayed**	**p-value**
2	1404	ade6-:: Sp wtf13::hphMX6	1920	ade6-:: Sp wtf13^{antidote}::kanMX4	181	152	54.4%	208	125	15	4.3	**62.46%**	37.54	333	348	5	0.08
	**allele 1 SZY#**	**GENOTYPE**	**allele 2 SZY#**	**GENOTYPE**	**ura+**	**ura-**	**% ura+ (control)**	**ade+**	**ade-**	**ade+ DRUG^R**	**% ade+ DRUG^R**	**% allele 1 (excluding ade+ DRUG^R)**	**% allele 2 (excluding ade+ DRUG^R)**	**# progeny assayed (w/o ade+ DRUG^R)**	**# progeny assayed**	**# diploids assayed**	**p-value**
	1920	ade6-:: Sp wtf13^{antidote}::kanMX4	320	ade6+	180	128	58.4%	173	135	9	2.8	43.83%	56.17	308	317	6	
	1922	ade6-:: Sp wtf13^{antidote}::kanMX4	320	ade6+	127	98	56.4%	109	116	7	3.0	51.56%	48.44	225	232	4	
3					307	226	57.6%	282	251	16	2.9	**47.09%**	52.91	533	549	10	0.81
4	1892	ade6-:: Sp wtf13^{poison}::hphMX6	174	ade6+	124	101	55.1%	125	100	7	3.0	**44.44%**	55.56	225	232	3	0.81
	**allele 1 SZY#**	**GENOTYPE**	**allele 2 SZY#**	**GENOTYPE**	**ura+**	**ura-**	**% ura+ (control)**	**GEN^R**	**GEN^S**	**HYG^R GEN^R**	**% HYG^R GEN^R**	**% allele 1 (excluding HYG^R GEN^R)**	**% allele 2 (excluding HYG^R GEN^R)**	**# progeny assayed (w/o HYG^R GEN^R)**	**# progeny assayed**	**# diploids assayed**	**p-value**
5	1922	ade6-:: Sp wtf13^{antidote}::kanMX4	1892	ade6-:: Sp wtf13^{poison}::hphMX6	143	83	63.3%	218	8	6	2.6	**96.46%**	3.54	226	232	4	2.37E-06
	**allele 1 SZY#**	**GENOTYPE**	**allele 2 SZY#**	**GENOTYPE**	**ura+**	**ura-**	**% ura+ (control)**	**HYG^R**	**HYG^S**	**HYG^R GEN^R**	**% HYG^R GEN^R**	**% allele 1 (excluding HYG^R GEN^R)**	**% allele 2 (excluding HYG^R GEN^R)**	**# progeny assayed (w/o HYG^R GEN^R)**	**# progeny assayed**	**# diploids assayed**	**p-value**
6	1404	ade6-:: Sp wtf13::hphMX6	1498	ade6-:: Sp wtf18::kanMX4	96	109	46.8%	204	1	18	8.1	**99.51%**	0.49	205	223	4	5.17E-07
7	1404	ade6-:: Sp wtf13::hphMX6	1667	ade6-:: Sp wtf18-2::kanMX4	142	92	60.7%	129	105	22	8.6	**55.13%**	44.87	234	256	4	0.4033
	**allele 1 SZY#**	**GENOTYPE**	**allele 2 SZY#**	**GENOTYPE**	**ura+**	**ura-**	**% ura+ (control)**	**ade+**	**ade-**	**ade+ DRUG^R**	**% ade+ DRUG^R**	**% allele 1 (excluding ade+ DRUG^R)**	**% allele 2 (excluding ade+ DRUG^R)**	**# progeny assayed (w/o ade+ DRUG^R)**	**# progeny assayed**	**# diploids assayed**	**p-value**
8	1667	ade6-:: Sp wtf18-2::kanMX4	320	ade6+	217	180	54.7%	180	217	9	2.2	**54.66%**	45.34	397	406	7	0.3789
	**allele 1 SZY#**	**GENOTYPE**	**allele 2 SZY#**	**GENOTYPE**	**ura+**	**ura-**	**% ura+ (control)**	**GEN^R**	**GEN^S**	**HYG^R GEN^R**	**% HYG^R GEN^R**	**% allele 1 (excluding HYG^R GEN^R)**	**% allele 2 (excluding HYG^R GEN^R)**	**# progeny assayed (w/o HYG^R GEN^R)**	**# progeny assayed**	**# diploids assayed**	**p-value**
9	1667	ade6-:: Sp wtf18-2::kanMX4	1892	ade6-:: Sp wtf13^{poison}::hphMX6	116	97	54.5%	204	9	19	8.2	**95.77%**	4.23	213	232	2	2.62E-05
	**allele 1 SZY#**	**GENOTYPE**	**allele 2 SZY#**	**GENOTYPE**	**ura+**	**ura-**	**% ura+ (control)**	**ade+**	**ade-**	**ade+ DRUG^R**	**% ade+ DRUG^R**	**% allele 1 (excluding ade+ DRUG^R)**	**% allele 2 (excluding ade+ DRUG^R)**	**# progeny assayed (w/o ade+ DRUG^R)**	**# progeny assayed**	**# diploids assayed**	**p-value**
10	1518	ade6-::hphMX6	320	ade6+	152	126	54.7%	143	135	11	3.8	**48.56%**	51.44	278	289	5	control
	**allele 1 SZY#**	**GENOTYPE**	**allele 2 SZY#**	**GENOTYPE**	**ura+**	**ura-**	**% ura+ (control)**	**HYG^R**	**HYG^S**	**HYG^R GEN^R**	**% HYG^R GEN^R**	**% allele 1 (excluding HYG^R GEN^R)**	**% allele 2 (excluding HYG^R GEN^R)**	**# progeny assayed (w/o HYG^R GEN^R)**	**# progeny assayed**	**# diploids assayed**	**p-value**
11	1404	ade6-:: Sp wtf13::hphMX6	1880	ade6-:: Sp wtf18-2$^{LGNAFGG\Delta}$::kanMX4	192	202	48.7%	386	8	36	8.4	**97.97%**	2.03	394	430	6	0.80
	1404	ade6-:: Sp wtf13::hphMX6	2247	ade6-:: Sp wtf18$^{+LGNAFGG}$::kanMX4	108	164	39.7%	250	22	18	6.2	91.91%	8.09	272	290	5	
	1404	ade6-:: Sp wtf13::hphMX6	2248	ade6-:: Sp wtf18$^{+LGNAFGG}$::kanMX4	93	126	42.5%	205	14	13	5.6	93.61%	6.39	219	232	4	
12					201	290	40.9%	455	36	31	5.9	**92.67%**	7.33	491	522	9	0.72
13	1404	ade6-:: Sp wtf13::hphMX6	2388	ade6-:: Sp wtf18-2^{D366NN}::kanMX4	74	141	34.4%	206	9	17	7.3	**95.81%**	4.19	215	232	4	0.95
	**allele 1 SZY#**	**GENOTYPE**	**allele 2 SZY#**	**GENOTYPE**	**ura+**	**ura-**	**% ura+ (control)**	**ade+**	**ade-**	**ade+ DRUG^R**	**% ade+ DRUG^R**	**% allele 1 (excluding ade+ DRUG^R)**	**% allele 2 (excluding ade+ DRUG^R)**	**# progeny assayed (w/o ade+ DRUG^R)**	**# progeny assayed**	**# diploids assayed**	**p-value**
21	1924	ade6-::Sp wtf13$^{intron1 \Delta}$::kanMX4	320	ade6+	233	146	61.5%	194	185	17	4.3	**48.81%**	51.19	379	396	8	0.9701
	**allele 1 SZY#**	**GENOTYPE**	**allele 2 SZY#**	**GENOTYPE**	**ura+**	**ura-**	**% ura+ (control)**	**GEN^R**	**GEN^S**	**HYG^R GEN^R**	**% HYG^R GEN^R**	**% allele 1 (excluding HYG^R GEN^R)**	**% allele 2 (excluding HYG^R GEN^R)**	**# progeny assayed (w/o HYG^R GEN^R)**	**# progeny assayed**	**# diploids assayed**	**p-value**
22	1404	ade6-:: Sp wtf13::hphMX6	1924	ade6-::Sp wtf13$^{intron1 \Delta}$::kanMX4	192	126	60.4%	175	143	25	7.3	**44.97%**	55.03	318	343	7	0.5971
23	1924	ade6-::Sp wtf13$^{intron1 \Delta}$::kanMX4	1892	ade6-:: Sp wtf13^{poison}::hphMX6	200	167	54.5%	339	28	30	7.6	**92.37%**	7.63	367	397	8	4.59E-07
24	1404	ade6-:: Sp wtf13::hphMX6	1878	ade6-:: Sp wtf18-2^{mut1}::kanMX4	176	141	55.5%	171	146	28	8.1	**53.94%**	46.06	317	345	6	1.74E-07
25	1404	ade6-:: Sp wtf13::hphMX6	1894	ade6-:: Sp wtf18-2^{mut2}::kanMX4	138	82	62.7%	126	94	12	5.1	**57.27%**	42.73	220	232	4	3.30E-05
	**allele 1 SZY#**	**GENOTYPE**	**allele 2 SZY#**	**GENOTYPE**	**ura+**	**ura-**	**% ura+ (control)**	**ade+**	**ade-**	**ade+ DRUG^R**	**% ade+ DRUG^R**	**% allele 1 (excluding ade+ DRUG^R)**	**% allele 2 (excluding ade+ DRUG^R)**	**# progeny assayed (w/o ade+ DRUG^R)**	**# progeny assayed**	**# diploids assayed**	**p-value**
26	2264	ade6-:: Sp wtf13$^{LGNAFGG \Delta}$::hphMX6	174	ade6+	119	151	44.1%	41	229	20	6.9	**84.82%**	15.18	270	290	5	control
	**allele 1 SZY#**	**GENOTYPE**	**allele 2 SZY#**	**GENOTYPE**	**ura+**	**ura-**	**% ura+ (control)**	**HYG^R**	**HYG^S**	**HYG^R GEN^R**	**% HYG^R GEN^R**	**% allele 1 (excluding HYG^R GEN^R)**	**% allele 2 (excluding HYG^R GEN^R)**	**# progeny assayed (w/o HYG^R GEN^R)**	**# progeny assayed**	**# diploids assayed**	**p-value**
27	2264	ade6-:: Sp wtf13$^{LGNAFGG \Delta}$::hphMX6	1667	ade6-:: Sp wtf18-2::kanMX4	98	111	46.9%	169	40	22	9.5	**80.86%**	19.14	209	231	4	0.73
28	2264	ade6-:: Sp wtf13$^{LGNAFGG \Delta}$::hphMX6	2247	ade6-:: Sp wtf18$^{+LGNAFGG}$::kanMX4	120	97	55.3%	171	46	15	6.5	**78.80%**	21.20	217	232	4	0.59
29	2264	ade6-:: Sp wtf13$^{LGNAFGG \Delta}$::hphMX6	1880	ade6-:: Sp wtf18-2$^{LGNAFGG\Delta}$::kanMX4	204	177	53.5%	249	132	25	6.2	**65.35%**	34.65	381	406	7	0.032
30	2264	ade6-:: Sp wtf13$^{LGNAFGG \Delta}$::hphMX6	1498	ade6-:: Sp wtf18::kanMX4	250	224	52.7%	312	162	48	9.2	**65.82%**	34.18	474	522	9	0.028
31	2264	ade6-:: Sp wtf13$^{LGNAFGG \Delta}$::hphMX6	2402	ade6-:: Sk wtf18::kanMX4	89	132	40.3%	116	105	10	4.3	**52.49%**	47.51	221	231	4	0.001
	**allele 1 SZY#**	**GENOTYPE**	**allele 2 SZY#**	**GENOTYPE**	**ura+**	**ura-**	**% ura+ (control)**	**ade+**	**ade-**	**ade+ DRUG^R**	**% ade+ DRUG^R**	**% allele 1 (excluding ade+ DRUG^R)**	**% allele 2 (excluding ade+ DRUG^R)**	**# progeny assayed (w/o ade+ DRUG^R)**	**# progeny assayed**	**# diploids assayed**	**p-value**
33	1554	ade6-:: Sp wtf13-YFP::kanMX4	320	ade6+	254	196	56.4%	129	321	13	2.8	**71.33%**	28.67	450	463	8	0.00243
	**allele 1 SZY#**	**GENOTYPE**	**allele 2 SZY#**	**GENOTYPE**	**ura+**	**ura-**	**% ura+ (control)**	**lys+**	**lys-**	**lys+ DRUG^R**	**% lys+ DRUG^R**	**% allele 1 (excluding lys+ DRUG^R)**	**% allele 2 (excluding lys+ DRUG^R)**	**# progeny assayed (w/o lys+ DRUG^R)**	**# progeny assayed**	**# diploids assayed**	**p-value**
34	1946	lys4-:: Sp wtf13-YFP::kanMX4	320	lys4+	179	124	59.1%	11	292	8	2.6	**96.37%**	3.63	303	311	3	------
	**allele 1 SZY#**	**GENOTYPE**	**allele 2 SZY#**	**GENOTYPE**	**ura+**	**ura-**	**% ura+ (control)**	**GEN^R**	**GEN^S**	**HYG^R GEN^R**	**% HYG^R GEN^R**	**% allele 1 (excluding HYG^R GEN^R)**	**% allele 2 (excluding HYG^R GEN^R)**	**# progeny assayed (w/o HYG^R GEN^R)**	**# progeny assayed**	**# diploids assayed**	**p-value**
35	1946	lys4-:: Sp wtf13-YFP::kanMX4	1830	lys4-:: Sp mCh-wtf18-2::hphMX6	158	124	56.03%	211	71	5	1.7	**74.82%**	25.18	282	287	5	------
