# Supplementary material for: A suppressor of a wtf poison-antidote meiotic driver acts via mimicry of the driver’s antidote
Source: PLoS Genet. 2018 Nov 26;14(11):e1007836. doi: 10.1371/journal.pgen.1007836 (PMC6283613; doi:10.1371/journal.pgen.1007836)
Supplement: S2 Table — Each of the rows represents the diploid assayed, which matches the diploid number in Figs 2, 3 and 4. The numbers underneath the diploid number are SZY numbers of the haploid parent strains. All the viable spore yield values are shown for each diploid. Diploids 1–9, 11, 12, 13, 21, 22–31 were normalized to control diploid 10. Diploids 14–19 were normalized to diploid 20. To determine any fertility defect in Fig 2, we compared diploids 1–9, 21, 22 and 23 to control diploid 10. To test if the wtf18 alleles rescued the fertility phenotype caused by the wtf13 driver, we compared diploid 6, 7, 11, 12 and 13 from Fig 3, and diploids 24 and 25 from S6 Fig to diploid 1. In S6 Fig, diploids 27–31 were compared to diploid 26 as control. Diploids in Fig 4 were all compared to diploid 20. We calculated the p-value for each diploid using the Wilcoxon test. (PDF) [file pgen.1007836.s014.pdf]

| | Figure 2 (VSY values) | | | | | | | | | | | | | | |
|---|---|---|---|---|---|---|---|---|---|---|---|---|---|---|---|
| | **Diploid 1** | | **Diploid 2** | **Diploid 3** | | **Diploid 4** | **Diploid 5** | **Diploid 6** | **Diploid 7** | **Diploid 8** | **Diploid 9** | **Diploid 10** | **Diploid 21** | **Diploid 22** | **Diploid 23** |
| | 1404x180 | 1403x174 | 1920x1404 | 1920x320 | 1922x320 | 1892x174 | 1922x1892 | 1498x1404 | 1404x1667 | 1667x320 | 1667x1892 | 1518x320 | 1924x320 | 1404x1924 | 1924x1892 |
| | 1.229 | 2.378 | 2.868 | 2.477 | 2.510 | 0.062 | 0.453 | 0.759 | 3.265 | 2.778 | 2.102 | 3.143 | 1.662 | 1.644 | 0.621 |
| | 1.050 | 1.796 | 1.962 | 1.952 | 2.588 | 0.044 | 0.373 | 1.389 | 4.316 | 1.412 | 1.449 | 4.106 | 2.170 | 3.529 | 1.153 |
| | 3.000 | 1.625 | 3.222 | 2.510 | 1.964 | 0.043 | 0.569 | 0.871 | 2.062 | 2.520 | 1.538 | 3.220 | 2.644 | 2.389 | 1.321 |
| | 2.667 | 1.850 | 2.962 | 2.018 | 1.821 | | 1.079 | 1.956 | 2.550 | 2.368 | 1.661 | 3.063 | 2.186 | 3.102 | 1.246 |
| | | | 2.089 | 1.979 | | | | | | 3.111 | 1.226 | 2.596 | 2.326 | 1.838 | 0.787 |
| | | | | 2.846 | | | | | | 2.155 | 1.308 | 2.649 | 3.028 | 2.060 | 0.703 |
| | | | | | | | | | | 1.067 | 1.323 | 2.920 | 1.690 | 2.581 | 0.612 |
| | | | | | | | | | | | 1.430 | | 2.348 | | 0.632 |
| average | 1.949 | | 2.621 | 2.267 | | 0.050 | 0.619 | 1.244 | 3.048 | 2.202 | 1.505 | 3.100 | 2.257 | 2.449 | 0.884 |
| stdev | 0.683 | | 0.560 | 0.355 | | 0.011 | 0.317 | 0.548 | 0.979 | 0.730 | 0.278 | 0.503 | 0.455 | 0.682 | 0.303 |
| p-value | 0.005905 | | 0.3434 | 0.001271 | | 0.01667 | 0.006061 | 0.006061 | 0.9273 | 0.01748 | 0.0003108 | control | 0.00373 | 0.07284 | 0.0003108 |
| relative fertility | **62.89%** | | **84.56%** | **73.13%** | | **1.61%** | **19.96%** | **40.12%** | **98.34%** | **71.03%** | **48.55%** | **100%** | **72.81%** | **79.01%** | **28.53%** |

| | Figure 3 (VSY values) | | | | | | | | Supplemental Figure 6 (VSY values) | | | | | | | |
|---|---|---|---|---|---|---|---|---|---|---|---|---|---|---|---|---|
| | **Diploid 1** | | **Diploid 6** | **Diploid 7** | **Diploid 11** | **Diploid 12** | | **Diploid 13** | **Diploid 24** | **Diploid 25** | **Diploid 26** | **Diploid 27** | **Diploid 28** | **Diploid 29** | **Diploid 30** | **Diploid 31** |
| | 1404x180 | 1403x174 | 1498x1404 | 1404x1667 | 1404x1880 | 1404x2447 | 1404x2448 | 1404x2388 | 1404x1878 | 1404x1894 | 2264x174 | 2264x1667 | 2264x2247 | 2264x1880 | 2264x1498 | 2264x2402 |
| | 1.229 | 2.378 | 0.759 | 3.265 | 1.596 | 1.733 | 1.609 | 2.23 | 2.92 | 2.23 | 1.789 | 2.071 | 2.042 | 3.170 | 1.667 | 2.148 |
| | 1.050 | 1.796 | 1.389 | 4.316 | 1.981 | 1.633 | 1.795 | 1.70 | 2.42 | 1.61 | 1.945 | 1.864 | 1.898 | 2.981 | 2.563 | 2.731 |
| | 3.000 | 1.625 | 0.871 | 2.062 | 1.364 | 1.857 | 2.150 | 1.67 | 2.62 | 3.19 | 1.522 | 1.966 | 1.241 | 2.583 | 1.881 | 3.340 |
| | 2.667 | 1.850 | 1.956 | 2.550 | 1.433 | 0.925 | 2.477 | 1.69 | 2.37 | 3.48 | 1.882 | 1.635 | 1.434 | 2.628 | 2.442 | 2.829 |
| | | | | | 3.000 | 1.340 | | | 2.79 | | 2.298 | | | 1.726 | 2.213 | |
| | | | | | 1.618 | | | | 4.06 | | | | | 2.128 | 2.324 | |
| | | | | | | | | | | | | | | 2.071 | 2.368 | |
| | | | | | | | | | | | | | | | 2.033 | |
| | | | | | | | | | | | | | | | 2.595 | |
| average | 1.949 | | 1.244 | 3.048 | 1.832 | 1.724 | | 1.823 | 2.863 | 2.628 | 1.887 | 1.884 | 1.654 | 2.470 | 2.232 | 2.762 |
| stdev | 0.683 | | 0.548 | 0.979 | 0.611 | 0.444 | | 0.271 | 0.623 | 0.865 | 0.281 | 0.187 | 0.378 | 0.519 | 0.315 | 0.489 |
| p-value | control | | 0.1535 | 0.07273 | 0.651 | 0.5414 | | 0.8081 | 0.05927 | 0.2828 | control | 0.9048 | 0.5556 | 0.07323 | 0.08292 | 0.03175 |
| relative fertility | **62.89%** | | **40.12%** | **98.34%** | **59.10%** | **55.63%** | | **58.82%** | **92.36%** | **84.79%** | **60.89%** | **60.78%** | **53.35%** | **79.68%** | **72.00%** | **89.12%** |

| | Figure 4 (VSY values) | | | | | | | | | | |
|---|---|---|---|---|---|---|---|---|---|---|---|
| | **Diploid 14** | | **Diploid 15** | **Diploid 16** | **Diploid 17** | | **Diploid 18** | | **Diploid 19** | **Diploid 20** | |
| | 1701x1444 | 1701x1445 | 1932x1541 | 1701x643 | 1541x1546 | 1545x1542 | 1541x643 | 1544x643 | 1481x1440 | 643x1919 | 643x1918 |
| | 2.213 | 2.409 | 1.557 | 1.667 | 1.167 | 1.239 | 3.203 | 1.035 | 3.161 | 2.277 | 2.267 |
| | 2.150 | 2.135 | 2.061 | 1.198 | 1.247 | 1.276 | 2.205 | 1.595 | 3.644 | 1.641 | |
| | 2.485 | 2.151 | 2.226 | 1.800 | 1.548 | 1.377 | | 2.398 | 1.836 | 1.836 | |
| | 3.182 | 1.750 | 2.592 | 1.783 | 1.096 | 2.111 | | | 1.983 | 1.962 | |
| | | | | 1.452 | | | | | 2.074 | 1.862 | |
| | | | | 1.841 | | | | | 1.720 | 2.532 | |
| | | | | 4.052 | | | | | | 1.697 | |
| average | 2.309 | | 2.109 | 1.970 | 1.383 | | 2.087 | | 2.403 | 2.009 | |
| stdev | 0.415 | | 0.429 | 0.946 | 0.324 | | 0.822 | | 0.798 | 0.313 | |
| p-value | 0.235 | | 0.808 | 0.189 | 0.003 | | 1.000 | | 0.401 | control | |
| relative fertility | **114.93%** | | **104.96%** | **98.06%** | **68.81%** | | **103.88%** | | **119.60%** | **100%** | |
