# Supplementary material for: A suppressor of a wtf poison-antidote meiotic driver acts via mimicry of the driver’s antidote
Source: PLoS Genet. 2018 Nov 26;14(11):e1007836. doi: 10.1371/journal.pgen.1007836 (PMC6283613; doi:10.1371/journal.pgen.1007836)
Supplement: S3 Table — Each of the horizontal lines represents the relevant genotype and allele transmission of the indicated diploid. The first column represents the diploid number, which matches the numbers in Fig 4. In columns 2–5, the strain number (SZY) and relevant genotype of the haploid parent strains used to determine the allele transmission at the Sp wtf13 or Sp wtf18-2 loci. Columns 6 and 7 indicate which phenotypes were followed at the control locus (ura4) and the number of progeny that showed the indicated phenotype. Columns 9 and 10 indicate the phenotypes that were followed at the Sp wtf13 or Sp wtf18-2 loci (bolded) and the number of haploid progeny that exhibited the indicated phenotype. Column 11 shows the fraction of the haploid progeny that inherited the genotype of allele 1. Column 12 shows the fraction of the haploid progeny that inherited the genotype of allele 2. Column 13 shows the total progeny assayed. Column 14 shows the total number of diploids assayed. Last column shows the p-value calculated by comparing diploids 16 and 17 to control diploid 14, and diploids 18 and 19 to control diploid 15. P-values were calculated using a G-test. (PDF) [file pgen.1007836.s015.pdf]

| Diploid # | allele 1 SZY# | GENOTYPE | allele 2 SZY# | GENOTYPE | ura+ | ura- | % ura+ (control) | HYG$^R$ | HYG$^S$ | % allele 1 | % allele 2 | # progeny assayed | # diploids assayed | p-value |
|---|---|---|---|---|---|---|---|---|---|---|---|---|---|---|
| 14 | 1444 | ***Sp wtf13* Δ::kanMX** *Sp wtf18-2+* | 1701 | ***Sp wtf13* Δ::hphMX** *Sp wtf18-2+* | 120 | 112 | 51.7% | 126 | 106 | 45.7% | 54.3% | 232 | 4 | |
| | 1445 | ***Sp wtf13* Δ::kanMX** *Sp wtf18-2+* | 1701 | ***Sp wtf13* Δ::hphMX** *Sp wtf18-2+* | 132 | 100 | 56.9% | 115 | 117 | 50.4% | 49.6% | 232 | 4 | |
| | | | | | 252 | 212 | 54.3% | 241 | 223 | **48.1%** | 51.9% | 464 | 8 | control |
| | **allele 1 SZY#** | **GENOTYPE** | **allele 2 SZY#** | **GENOTYPE** | **ura+** | **ura-** | **% ura+ (control)** | **GEN$^R$** | **GEN$^S$** | **% allele 1** | **% allele 2** | **# progeny assayed** | **# diploids assayed** | **p-value** |
| 15 | 1932 | *Sp wtf13+* ***Sp wtf18-2* Δ::hphMX** | 1541 | *Sp wtf13+* ***Sp wtf18-2* Δ::kanMX** | 132 | 100 | 56.9% | 134 | 98 | **42.2%** | 57.8% | 232 | 4 | control |
| **Diploid #** | **allele 1 SZY#** | **GENOTYPE** | **allele 2 SZY#** | **GENOTYPE** | **ura+** | **ura-** | **% ura+ (control)** | **HYG$^R$** | **HYG$^S$** | **% allele 1** | **% allele 2** | **# progeny assayed** | **# diploids assayed** | **p-value** |
| 16 | 643 | ***Sp wtf13+*** *Sp wtf18-2+* | 1701 | ***Sp wtf13* Δ::hphMX** *Sp wtf18-2+* | 244 | 162 | 60.1% | 200 | 206 | **50.7%** | 49.3% | 406 | 7 | 0.6462 |
| 17 | 1541 | ***Sp wtf13+*** *Sp wtf18-2* Δ::kanMX | 1546 | ***Sp wtf13* Δ::hphMX** *Sp wtf18-2* Δ::kanMX | 148 | 142 | 51.0% | 22 | 268 | 92.4% | 7.6% | 290 | 4 | |
| | 1542 | ***Sp wtf13+*** *Sp wtf18-2* Δ::kanMX | 1545 | ***Sp wtf13* Δ::hphMX** *Sp wtf18-2* Δ::kanMX | 169 | 121 | 58.3% | 53 | 237 | 81.7% | 18.3% | 290 | 4 | |
| | | | | | 317 | 263 | 54.7% | 75 | 505 | **87.1%** | 12.9% | 580 | 8 | 3.47E-09 |
| | **allele 1 SZY#** | **GENOTYPE** | **allele 2 SZY#** | **GENOTYPE** | **ura+** | **ura-** | **% ura+ (control)** | **GEN$^R$** | **GEN$^S$** | **% allele 1** | **% allele 2** | **# progeny assayed** | **# diploids assayed** | **p-value** |
| 18 | 643 | *Sp wtf13+* ***Sp wtf18-2+*** | 1541 | *Sp wtf13+* ***Sp wtf18-2* Δ::kanMX** | 42 | 74 | 36.2% | 59 | 57 | 49.1% | 50.9% | 116 | 2 | |
| | 643 | *Sp wtf13+* ***Sp wtf18-2+*** | 1544 | *Sp wtf13+* ***Sp wtf18-2* Δ::kanMX** | 100 | 74 | 57.5% | 99 | 75 | 43.1% | 56.9% | 174 | 3 | |
| | | | | | 142 | 148 | 49.0% | 158 | 132 | **45.5%** | 54.5% | 290 | 5 | 0.64 |
| 19 | 1440 | *Sp wtf13* Δ:: hphMX, ***Sp wtf18-2+*** | 1481 | *Sp wtf13* Δ::hphMX, ***Sp wtf18* Δ::kanMX** | 137 | 95 | 59.1% | 114 | 118 | **50.9%** | 49.1% | 232 | 2 | 0.2605 |
| 20 | 643 | *Sp wtf13+ Sp wtf18-2+* | 1919 | *Sp wtf13+ Sp wtf18-2+* | 130 | 102 | 56.0% | ---- | ---- | ---- | ---- | 232 | 2 | ---- |
