# Supplementary material for: A suppressor of a wtf poison-antidote meiotic driver acts via mimicry of the driver’s antidote
Source: PLoS Genet. 2018 Nov 26;14(11):e1007836. doi: 10.1371/journal.pgen.1007836 (PMC6283613; doi:10.1371/journal.pgen.1007836)
Supplement: S4 Table — Each of the horizontal lines represents the relevant genotype and allele transmission of the indicated diploid. The first column shows the Fig where these data were represented, or the diploid number referred to in the text. In columns 2–5, the strain number (SZY) and relevant genotype of the haploid parent strains used to determine the allele transmission are shown. Diploids with the indicated genotype will generate four different types of spores (column 6–9). Columns 6 and 7 are the parental classes and columns 8 and 9 are the recombinant classes. Column 10 shows the total progeny assayed. Column 11 shows the total number of diploids assayed. (PDF) [file pgen.1007836.s016.pdf]

					PARENTAL		RECOMBINANT			
	**allele 1 SZY#**	**GENOTYPE**	**allele 2 SZY#**	**GENOTYPE**	**$Gen^S Hyg^S$**	**$Gen^R Hyg^R$**	**$Gen^S Hyg^R$**	**$Gen^R Hyg^S$**	**# progeny assayed**	**# diploids assayed**
**Figure 4B**	1481	*Sp wtf13* Δ*::hphMX wtf18-2* Δ*::kanMX*	643	*Sp wtf13+ wtf18-2+*	91	10	36	37	174	3
	1482	*Sp wtf13* Δ*::hphMX wtf18-2* Δ*::kanMX*	643	*Sp wtf13+ wtf18-2+*	122	16	49	45	232	4
					213	26	85	82	406	7
	**allele 1 SZY#**	**GENOTYPE**	**allele 2 SZY#**	**GENOTYPE**	**lys-ade+**	**lys+ade-**	**lys+ade+**	**lys-ade-**	**# progeny assayed**	**# diploids assayed**
**diploid 32**	1830	*lys4-:: Sp mCh-wtf18-2::hphMX, ade6+*	1554	*lys4+, ade6-:: Sp wtf13-YFP::kanMX*	122	127	49	138	436	8
