# Supplementary material for: A suppressor of a wtf poison-antidote meiotic driver acts via mimicry of the driver’s antidote
Source: PLoS Genet. 2018 Nov 26;14(11):e1007836. doi: 10.1371/journal.pgen.1007836 (PMC6283613; doi:10.1371/journal.pgen.1007836)
Supplement: S5 Table — (PDF) [file pgen.1007836.s017.pdf]

**Plasmids**	**short description**	**reference**
pFA6	contains kanMX4	53
pAG32	contains hphMX6	53
pFA6-EYFP-HIS3MX	contains eYFP	54
pFA6-mTurq2-URA3MX	contains mTurq2	55
pSZB188	derivative of pFA6 that integrates at *ade6* , yielding *ade6* -	7
pSZB322	derivative of pAG32 that integrates at *lys4* , yielding *lys4-*	this work
pSZB329	derivative of pFA6 that integrates at *lys4* , yielding *lys4-*	this work
pSZB368	pSZB188 with *Sp wtf13* cloned into SacI site	this work
pSZB386	derivative of pAG32 that integrates at *ade6* , yielding *ade6* -	this work
pSZB470	pSZB188 with *Sk wtf18* cloned into SacI site	this work
pSZB483	pSZB188 with *Sp wtf18* cloned into SacI site	this work
pSZB485	pSZB386 with *Sp wtf18* cloned into SacI site	this work
pSZB495	pSZB386 with *Sp wtf13* cloned into SacI site	this work
pSZB496	pSZB386 with *Sp wtf13* cloned into SacI site	this work
pSZB522	pSZB188 with *Sp wtf13-YFP* cloned into SacI site	this work
pSZB526	pSZB386 with *Sp wtf13-mTq* cloned into SacI site (non-functional)	this work
pSZB565	pSZB386 with *Sp wtf13(M1X, M12X)-mTq* cloned into SacI site (non-functional)	this work
pSZB566	pSZB188 with *Sp wtf18-2* cloned into SacI site	this work
pSZB625	pSZB322 with *Sp mCh-wtf18-2* cloned into SacI site	this work
pSZB673	pSZB188 with *Sp wtf18-2* (p. 333-339Δ*) cloned into SacI site*	this work
pSZB674	pSZB188 with *Sp wtf18-2* (D366N, D366_I367insN) cloned into SacI site	this work
pSZB675	pSZB188 with *Sp wtf18-2* (D366N) cloned into SacI site	this work
pSZB686	pSZB386 with *Sp wtf13(M1X, M12X)* cloned into SacI site	this work
pSZB688	pSZB188 with *Sp wtf18-2* (G370E, M372T, D373E, V374A) cloned into SacI site	this work
pSZB702	pSZB188 with *Sp wtf13(358A>T, 359T>A, 360G>C)* cloned into SacI site	this work
pSZB703	pSZB188 with *Sp wtf13(358A>T, 359T>A, 360G>C)* cloned into SacI site	this work
pSZB706	pSZB329 with *Sp wtf13-YFP* cloned into SacI site	this work
pSZB806	pSZB386 with *Sp wtf13* (p.343-349Δ)cloned into SacI site	this work
pSZB807	pSZB188 with *Sp wtf18* (G332_I333insLGNAFGG) cloned into SacI site	this work
pSZB808	pSZB188 with *Sp wtf18* (G332_I333insLGNAFGG) cloned into SacI site	this work
