# Supplementary material for: A suppressor of a wtf poison-antidote meiotic driver acts via mimicry of the driver’s antidote
Source: PLoS Genet. 2018 Nov 26;14(11):e1007836. doi: 10.1371/journal.pgen.1007836 (PMC6283613; doi:10.1371/journal.pgen.1007836)
Supplement: S6 Table — (PDF) [file pgen.1007836.s018.pdf]

oligo	sequence (5' --> 3')
588	ATGAGCGAAAAACAGGTTGTAGGGATC
589	GGTACCTGACCTGAATTGTGAGGCCGAGG
590	CCATAGCAGCCAAAAGGGAGGGTTG
591	CACAATTCAGGTCAGGTACCCAACACCCAACTCTCGACTTCCAC
598	CGGGTAAGTAAAGAATCATTCATACAGTTGG
601	CCC TTC TGA GAC TAC TAA TAT CAG TTC TTG
634	AATATAGAGCTCCGGGGACGAGGCAAGCTAAAC
879	ATATATGAGCTCTCATTTGGCTCCTTCTTGTTGT
880	ATATATGAGCTCTGTGCCTCTAATGATCTAGCGT
985	TACCCATGCGAGCCAGATTGTAAC
1037	ATTATT GAGCTC CAAGTTAAACCATTATTGTTTGTGTCTTGGGTTCC
1039	ATTATT GAGCTC ATCAACACGATAGCATGAGACCTTCTTACCAT
1048	CAGCTTCGGTAGAAACTTTGCGTCAAAATC
1049	GTGTCACCTAAATCGTATGTG GCGACAAACGAAACAGCGAAATCAAGAATA
1050	TATTCTTGATTTCGCTGTTTCGTTTGTCGC CACATACGATTTAGGTGACAC
1051	ACTGGAGCGACAACCACATTAATAAAGCAA AATACGACTCACTATAGGGAG
1052	CTCCCTATAGTGAGTCGTATT TTGCTTTATTAATGTGGTTGTCGCTCCAGT
1053	TTCGCTGTTGACTTAGAAATCCCTGCCTAA
1058	AGTTGCATACATTACCAAAGAAGCGTTGAATC
1061	CATGCACGTTACCACATCAAAAGTAGTAGGAA
1078	GTATACGAACCTGACGTTGTCAGACTCCAC
1079	GTGTCACCTAAATCGTATGTG CACATGCGCGAATTAATTGCTGTGATTGC
1080	GCAATCACAGCAATTAATTCGCGCATGTG CACATACGATTTAGGTGACAC
1081	CCAACTGGAGCGACAACTACAATAATAAAGCAA AATACGACTCACTATAGGGAG
1082	CTCCCTATAGTGAGTCGTATT TTGCTTTATTATTGTAGTTGTCGCTCCAGTTGG
1083	TGGCCGACTGTAAACAATGACCAAAGC
1113	CTGCAGCGTGATAAGACTCCATGGC
1114	CAGGGTTCATCTCATGCAATGGAATAGCT
1115	AATATAGAGCTC GTGCCTCTAATGATCTAGCGTCAGAACATTAG
1123	TGACATTCCCTTGGGAGAAATGGATGTTGAAAGCGAAGTCGGTCGACGGATCCCCGGGTT
1124	TGACATTCCCTTGGGAGAAATGGATGTTGAAAGCGAAGTCGGTGACGGTGCTGGTTTA
1125	AACCCGGGGATCCGTCGACCGACTTCGCTTTCAACATCCATTTCTCCCAAGGGAATGTCA
1126	TAAACCAGCACCGTCACCGACTTCGCTTTCAACATCCATTTCTCCCAAGGGAATGTCA
1202	ATTATT GAGCTC CGTTCTGAGTCACAATATTTTATTATGCTATTC
1254	CCTGAATATAGAGGCAGTGTGCCCTCTT
1255	ATGAAGAATAATTACACTTCCTTGAAAAGTCCTCTAG
1394	CCAAATTTCAAAAGTTATTTATTTTATTATACCTTTCAGAAATTTGGAAATATATTAAAACTGTATCTGAGG
1395	CCTCAGATACAGTTTTAATATATTTCCAAATTTCTGAAAGGTATAATAAAATAAATAACTTTTGAAATTTGG
1530	GCTATACCATTCCCGATAAATTTGGGC
1659	GTATGAATGATTTTTTACTTACCCGACTAGGAGGGATAGGAAATGCATTTGGAGGGATAG
1660	CTATCCCTCCAAATGCATTTCCTATCCCTCCTAGTCGGGTAAGTAAAAAATCATTCATAC
1661	GTTTAGAGGTGCCAACGATAATAATAATAATATTCCCTTGGGAGAAATGGATGTTGAAAG
1662	CTTTCAACATCCATTTCTCCCAAGGGAATATTATTATTATTATCGTTGGCACCTCTAAAC
1663	GTTTAGAGGTGCCAACGATAATAATAATATTCCCTTGGGAGAAATGGATGTTG
1664	CAACATCCATTTCTCCCAAGGGAATATTATTATTATCGTTGGCACCTCTAAAC
1665	CCAACGATAATAATGACATTCCCTTGGAAGAAACGGAGGCCGAAAGCGAAGTCTAATTGC
1666	GCAATTAGACTTCGCTTTCGGCCTCCGTTTCTTCCAAGGGAATGTCATTATTATCGTTGG
