# Supplementary material for: A suppressor of a wtf poison-antidote meiotic driver acts via mimicry of the driver’s antidote
Source: PLoS Genet. 2018 Nov 26;14(11):e1007836. doi: 10.1371/journal.pgen.1007836 (PMC6283613; doi:10.1371/journal.pgen.1007836)
Supplement: S7 Table — Notes: The ura4-X allele in SZY562 and SZY581 listed in the table is either ura4-D18 or ura4-294. Sp wtf13 (M1X, M12X) is the Sp wtf13 poison allele. Sp wtf18-2 (D366N) is the Sp wtf18-2mut1 allele used in S6 Fig. The Sp wtf18-2 (G370E, M372T, D373E, V374A) allele is Sp wtf18-2mut2 in S6 Fig. The Sp wtf18-2 (p.333-339Δ) allele contains the first repeat unit deleted. Sp wtf18 (G332_I333insLGNAFGG) is the wtf18+rep allele in which we inserted the first repeat unit between residues 332 and 333. Sp wtf18-2 (D366N, D366_I367insN) is the Sp wtf18-2D366NN allele used in Fig 3. Sp wtf13 (p.343-349Δ) is an allele containing the deletion of the first repeat unit. We are listing the mutations in the protein. Sp wtf13 (358A>T, 359T>A, 360G>C) is the Sp wtf13 antidote allele. We are listing the mutations in the nucleotide sequence. (PDF) [file pgen.1007836.s019.pdf]

Strain	Other name	Species	Genotype
GP52		*Sp*	*h90, ade6-M375*
GP745	SZY44	*Sp*	*h-, lys4-95*
GP7596		*Sp*	*h-, ura4-D18, his3-D1, leu1-32, arg3-D1*
SZY13		*Sk*	*wild-type*
SZY174		*Sk*	*h90, his5Δ::natMX4*
SZY180		*Sk*	*h90, lys1Δ::kanMX4*
SZY320		*Sk*	*h90, ura4Δ::natMX4*
SZY562		hybrid with *Sk* karyotype	*hybrid chr3, Sk chr1 and chr2. h90, rec12Δ::ura4+, his5Δ::natMX4, ura4-x, ade6Δ::hphMX6*
SZY581		hybrid with *Sk* karyotype	*hybrid chr3, Sk chr1 and chr2. h90, rec12Δ::ura4+, his5Δ::natMX4, ura4-x*
SZY589		hybrid with *Sk* karyotype	*hybrid chr3, Sk chr1 and chr2. h90, rec12Δ::ura4+, his5Δ::natMX4, ura4Δ::kanMX4*
SZY591		hybrid with *Sk* karyotype	*hybrid chr3, Sk chr1 and chr2. h90, rec12Δ::ura4+, his5Δ::natMX4, ura4Δ::kanMX4*
SZY629		*Sp*	*h90, arg3-D1, his3-D1, wtf18-2*
SZY631		*Sp*	*h-, ade6-M375, leu1-32, ura4-D18*
SZY643		*Sp*	*h90, leu1-32, ura4-D18 wtf18-2*
SZY1049		*Sp*	*h90, leu1-32, ura4-D18, ade6-::Sk wtf4(M1X, M12X)-GFP::kanMX4::ade6-*
SZY1142		*Sp*	*h90, ura4-D18, his5Δ::ade6+, lys4-95, ade6-::Sk mCherry-wtf4::kanMX4::ade6-*
SZY1403		*Sk*	*h90, ura4Δ::natMX4, ade6-::Sp wtf13::hphMX6::ade6-*
SZY1404		*Sk*	*h90, ura4Δ::natMX4, ade6-::Sp wtf13::hphMX6::ade6-*
SZY1440		*Sp*	*h90, leu1-32, ura4-D18, wtf13Δ::hphMX6,* wtf18-2
SZY1441		*Sp*	*h90, leu1-32, ura4-D18, wtf13Δ::hphMX6,* wtf18-2
SZY1442		*Sp*	*h-, lys4-95, wtf13Δ::hphMX6*
SZY1444		*Sp*	*h90, leu1-32, ura4-D18, wtf13Δ::kanMX4, wtf18-2*
SZY1445		*Sp*	*h90, leu1-32, ura4-D18, wtf13Δ::kanMX4, wtf18-2*
SZY1446		*Sp*	*h-, lys4-95, wtf13Δ::kanMX4*
SZY1481		*Sp*	*h-, lys4-95, wtf13Δ::hphMX6, wtf18Δ::kanMX4*
SZY1482		*Sp*	*h-, lys4-95, wtf13Δ::hphMX6, wtf18Δ::kanMX4*
SZY1483		*Sp*	*h-, lys4-95, wtf13Δ::kanMX4, wtf18Δ::hphMX6*
SZY1496		*Sk*	*h90, his5Δ::natMX4, ade6-::Sp wtf13::kanMX4::ade6-*
SZY1498		*Sk*	*h90, his5Δ::natMX4, ade6-::Sp wtf18::kanMX4::ade6-*
SZY1518		*Sk*	*h90, his5Δ::natMX4, ade6-::hphMX6::ade6-*
SZY1541		*Sp*	*h90, lys4-95, wtf18Δ::kanMX4*
SZY1542		*Sp*	*h?, leu1-32, lys4-95, wtf18Δ::kanMX4*
SZY1544		*Sp*	*h?, lys4-95, wtf18Δ::kanMX4*
SZY1545		*Sp*	*h90, ura4-D18, wtf13Δ::hphMX6, wtf18Δ::kanMX4*
SZY1546		*Sp*	*h?, leu1-32, ura4-D18, wtf13Δ::hphMX6, wtf18Δ::kanMX4*
SZY1554		*Sk*	*h90, his5Δ::natMX4, ade6-::Sp wtf13-YFP::kanMX4::ade6-*
SZY1555		*Sk*	*h90, his5Δ::natMX4, ade6-::Sp wtf13-YFP::kanMX4::ade6-*
SZY1667		*Sk*	*h90, his5Δ::natMX4, ade6-::Sp wtf18-2::kanMX4::ade6-*
SZY1701		*Sp*	*h?, lys4-95, wtf13Δ::hphMX6 wtf18-2*
SZY1830		*Sk*	*h90, ura4Δ::natMX4, lys4-::Sp mCherry-wtf18-2::hphMX6::lys4-*
SZY1878		*Sk*	*h90, his5Δ::natMX4, ade6-::Sp wtf18-2 (p.D366N)::kanMX4::ade6-*
SZY1880		*Sk*	*h90, his5Δ::natMX4, ade6-::Sp wtf18-2 (p. 333-339Δ)::kanMX4::ade6-*
SZY1892		*Sk*	*h90, uraΔ::natMX4, ade6-::Sp wtf13* (M1X, M12X)*::hphMX6::ade6-*
SZY1894		*Sk*	*h90, his5Δ::natMX4, ade6-::Sp wtf18-2 (G370E, M372T, D373E, V374A)::kanMX4::ade6-*
SZY1918		*Sp*	*h90, lys4-95, wtf18-2*
SZY1919		*Sp*	*h?, lys4-95, wtf18-2*
SZY1920		*Sk*	*h90, his5Δ::natMX4, ade6-::Sp wtf13* (358A>T, 359T>A, 360G>C)*::kanMX4::ade6-*
SZY1922		*Sk*	*h90, his5Δ::natMX4, ade6-::Sp wtf13* (358A>T, 359T>A, 360G>C)*::kanMX4::ade6-*
SZY1924		*Sk*	*h90, his5Δ::natMX4, ade6-::Sp* $wtf13^{intron1\Delta}$*::kanMX4::ade6-*
SZY1932		*Sp*	*h?, leu1-32, ura4-D18, wtf18Δ::hphMX6*
SZY1946		*Sk*	*h90, his5Δ::natMX4, lys4-::Sp wtf13-YFP::kanmX4::lys4-*
SZY2247		*Sk*	*h90, his5Δ::natMX4, ade6-::Sp wtf18 (G332_I333insLGNAFGG)::kanMX4::ade6-*
SZY2248		*Sk*	*h90, his5Δ::natMX4, ade6-::Sp wtf18 (G332_I333insLGNAFGG)::kanMX4::ade6-*
SZY2264		*Sk*	*h90, ura4Δ::natMX4, ade6-::Sp wtf13 (p.343-349Δ)::hphMX6::ade6-*
SZY2388		*Sk*	*h90, his5Δ::natMX4, ade6-::Sp wtf18-2 (D366N, D366_I367insN)::kanMX4::ade6-*
SZY2402		*Sk*	*h90, his5Δ::natMX4, ade6-::Sk wtf18::kanMX4::ade6-*
